# Exon architecture controls mRNA m^6^A suppression and gene expression

**DOI:** 10.1126/science.abj9090

**Published:** 2023-01-27

**Authors:** P. Cody He, Jiangbo Wei, Xiaoyang Dou, Bryan T. Harada, Zijie Zhang, Ruiqi Ge, Chang Liu, Li-Sheng Zhang, Xianbin Yu, Shuai Wang, Ruitu Lyu, Zhongyu Zou, Mengjie Chen, Chuan He

**Affiliations:** 1 Department of Chemistry, Department of Biochemistry and Molecular Biology, Institute for Biophysical Dynamics, The University of Chicago, Chicago, IL 60637, USA.; 2 Committee on Immunology, The University of Chicago, Chicago, IL 60637, USA.; 3 Howard Hughes Medical Institute, The University of Chicago, Chicago, IL 60637, USA.; 4 State Key Laboratory for Conservation and Utilization of Bio-Resources, School of Life Sciences, Yunnan University, Kunming, Yunnan 650091, China.; 5 Department of Neurobiology, The University of Chicago, Chicago, IL 60637, USA.; 6 Department of Human Genetics, The University of Chicago, Chicago, IL 60637, USA.; 7 Section of Genetic Medicine, Department of Medicine, The University of Chicago, Chicago, IL 60637, USA.

## Abstract

*N*^6^–methyladenosine (m^6^A) is the most abundant mRNA modification and plays crucial roles in diverse physiological processes. Utilizing a Massively Parallel Assay for m^6^A (MPm^6^A), we discover that m^6^A specificity is globally regulated by “suppressors” that prevent m^6^A deposition in unmethylated transcriptome regions. We identify Exon Junction Complexes (EJCs) as m^6^A suppressors that protect exon junction-proximal RNA within coding sequences from methylation and regulate mRNA stability through m^6^A suppression. EJC suppression of m^6^A underlies multiple global characteristics of mRNA m^6^A specificity, with the local range of EJC protection sufficient to suppress m^6^A deposition in average-length internal exons, but not in long internal and terminal exons. EJC-suppressed methylation sites co-localize with EJC-suppressed splice sites, suggesting that exon architecture broadly determines local mRNA accessibility to regulatory complexes.

*N*^6^–methyladenosine (m^6^A), the most prevalent mRNA modification in mammals, influences wide-ranging aspects of gene expression in diverse physiological and pathophysiological processes (1–3). The METTL3-METTL14 methyltransferase complex installs m^6^A methylation on mRNA in a common DRACH (D = A, G, or U; R= A or G; H= A, C, or U) sequence motif, but only a fraction of DRACH sequences (~5%) in a subset of cellular transcripts are selected for methylation (4). Additionally, m^6^A exhibits a marked regional bias in its transcriptomic distribution, being strongly enriched in unusually long internal exons and near stop codons (5, 6). Despite the central importance of specific m^6^A deposition in m^6^A-mediated gene regulation, the mechanistic basis for m^6^A specificity has remained poorly understood.

In this study, we discover the existence of prevalent regulatory mechanisms that restrict m^6^A methylation to specific transcript regions through targeted suppression of m^6^A in unmethylated regions. We find that pre-mRNA splicing selectively suppresses m^6^A deposition in average-length internal exons, but not in longer exons. We identify Exon Junction Complexes (EJC) as major m^6^A suppressors that mediate this effect and control several key characteristics of global m^6^A specificity. EJC depletion results in pervasive aberrant methylation of mRNAs and m^6^A-mediated transcript destabilization. EJCs, together with interacting proteins, package and protect long stretches of proximal RNA from cellular methylation deposition, which may represent a general mechanism by which exon architecture and EJC positioning determine local mRNA accessibility to regulatory machineries.

## Massively Parallel Assay for m^6^A

The extent to which global m^6^A specificity is controlled has important implications for m^6^A regulation but is poorly understood (4). We approached this problem by asking: is the local sequence surrounding an m^6^A methylated site, when uncoupled from its endogenous context, sufficient to specify methylation at that site? Conversely, is the local sequence surrounding an unmethylated DRACH site, when uncoupled from its endogenous context, sufficient to prevent methylation at that site?

**Fig. 1.**
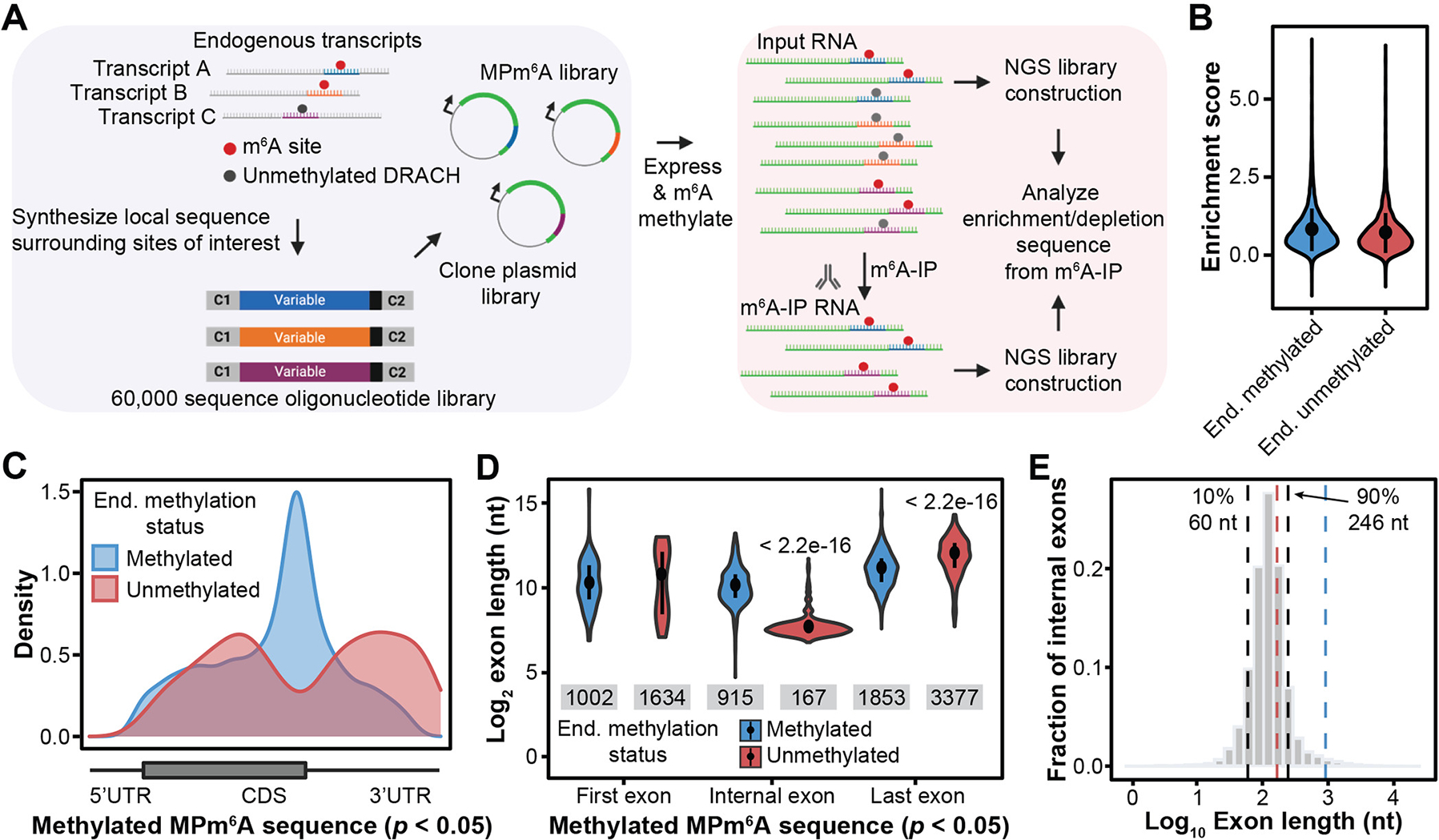
MPm^6^A reveals suppression of thousands of m^6^A sites in unmethylated transcriptome regions. (**A**) Schematic of the MPm^6^A workflow. (**B**) MPm^6^A enrichment scores (experimental IP/input – negative control IP/input) for endogenously methylated (*n* = 6,095) and unmethylated (*n* = 2,716) sequences, mean ± SD, four biological replicates. (**C**) Metagenes of endogenously methylated and unmethylated sequences that are significantly methylated in MPm^6^A. (**D**) Exon lengths of endogenously methylated and unmethylated sequences that are significantly methylated in MPm^6^A. Median, and IQR, Wilcoxon rank sum test. Sample size for each violin plot from left to right is: *n* = 175, *n* = 22, *n* = 696, *n* = 519, *n* = 3,539, and *n* = 1,328. (**E**) Distribution of internal exon lengths in the human genome. Black lines indicate 10^th^ percentile (left, 60 nt) and 90^th^ percentile (right, 246 nt). Blue and red lines indicate median internal exon length for MPm^6^A endogenously methylated (915 nt) and unmethylated (167 nt) sequences, respectively.

To assess this systematically on an epitranscriptome-wide scale, we developed a Massively Parallel Reporter Assay (MPRA) that enables high-throughput assessment of the m^6^A methylation status of thousands of designed sequences, which we termed Massively Parallel assay for m^6^A (MPm^6^A) (Fig. 1A and fig. S1A). In the MPm^6^A workflow, thousands of endogenously methylated m^6^A sites and endogenously unmethylated DRACH sites, with 102 nucleotides of sequence surrounding each site, were synthesized and cloned into the 3′UTR of a plasmid-based intronless GFP transgene. For each sequence, we also designed a corresponding negative control sequence in which all DRACH motifs were mutated to prevent methylation. The sequences were expressed and then m^6^A methylated through transfection into cells, or, when specified, through in vitro transcription and in vitro m^6^A methylation. The methylation status of each individual sequence was assessed by its enrichment following m^6^A-immunoprecipitation (IP) of mRNA. We selected 6,897 HeLa m^6^A sites and 3,058 unmethylated DRACH sites to assay in HeLa cells and validated the assay’s precision and accuracy (fig. S1, B to D).

## Widespread mRNA m^6^A suppression controls m^6^A epitranscriptome specificity

When we compared the methylation levels of the endogenously methylated sequences to their negative control sequences, we found that 92.8% of the sequences exhibited significant methylation in this reporter assay (Fig. 1B and fig. S1E), indicating that most endogenously methylated sites do not strictly require their larger surrounding native context for methylation. Unexpectedly, 90.2% of endogenously unmethylated sequences also exhibited significant methylation (Fig. 1B and fig. S1E). The MPm^6^A enrichment scores of the endogenously unmethylated group were similar to the endogenously methylated group, despite their diverging endogenous methylation states (Fig. 1B). We observed similar results when the sequences were in vitro transcribed and methylated with recombinant METTL3-METTL14 (fig. S2). Thus, thousands of endogenously unmethylated DRACH sites became methylated when they were uncoupled from their endogenous contexts and expressed in an artificial reporter context. We term these identified sites “suppressed m^6^A sites”. We validated these results for three selected sequences (fig. S1F), and confirmed that methylation was not notably influenced by the CMV promoter of the MPm^6^A plasmid (fig. S3). We observed similar results when sequences were expressed within CDS or 5′UTR, though m^6^A enrichment was significantly lower for many sequences when placed in the CDS or 5′UTR versus in the 3′UTR (fig. S4, A to D). This suggests that 5′ regions are generally less conducive for m^6^A methylation than 3′ regions (fig. S4E). Collectively, these results reveal the existence of thousands of suppressed m^6^A sites that are silenced by unknown mechanisms.

We noted that suppressed m^6^A sites were enriched in the CDS and 3′UTR and were depleted near the stop codon, which is the reverse of endogenous m^6^A site enrichment (Fig. 1C and fig. S5A). Further, suppressed m^6^A sites in internal exons reside within much shorter exons (median = 167 nt) than endogenous m^6^A sites (median = 915 nt) (Fig. 1D). These observations suggest that endogenous m^6^A enrichment in long internal exons may be a consequence of suppression of m^6^A deposition in shorter internal exons, which comprise most exons (90% of internal exons are < 246 nt) (Fig. 1E). 942 genes containing suppressed m^6^A sites did not contain any endogenous m^6^A peaks on their transcripts (fig. S5B). Suppression of these sites appears to involve suppression of m^6^A deposition rather than active demethylation, as binding sites for RBM15, a METTL3-METTL14 methyltransferase complex accessory subunit, were highly enriched near endogenous m^6^A sites compared to suppressed m^6^A sites (fig. S6A), while FTO and ALKBH5 binding sites were not significantly enriched near suppressed sites and exhibited little binding near suppressed sites overall (fig. S6B). These results were unexpected as previous reports on m^6^A specificity had mainly focused on activating mechanisms (7–12). Our MPm^6^A assay suggests the existence of unknown m^6^A “suppressors” that govern global m^6^A specificity by suppressing m^6^A deposition.

## Pre-mRNA splicing suppresses m^6^A methylation proximal to splice sites

**Fig. 2.**
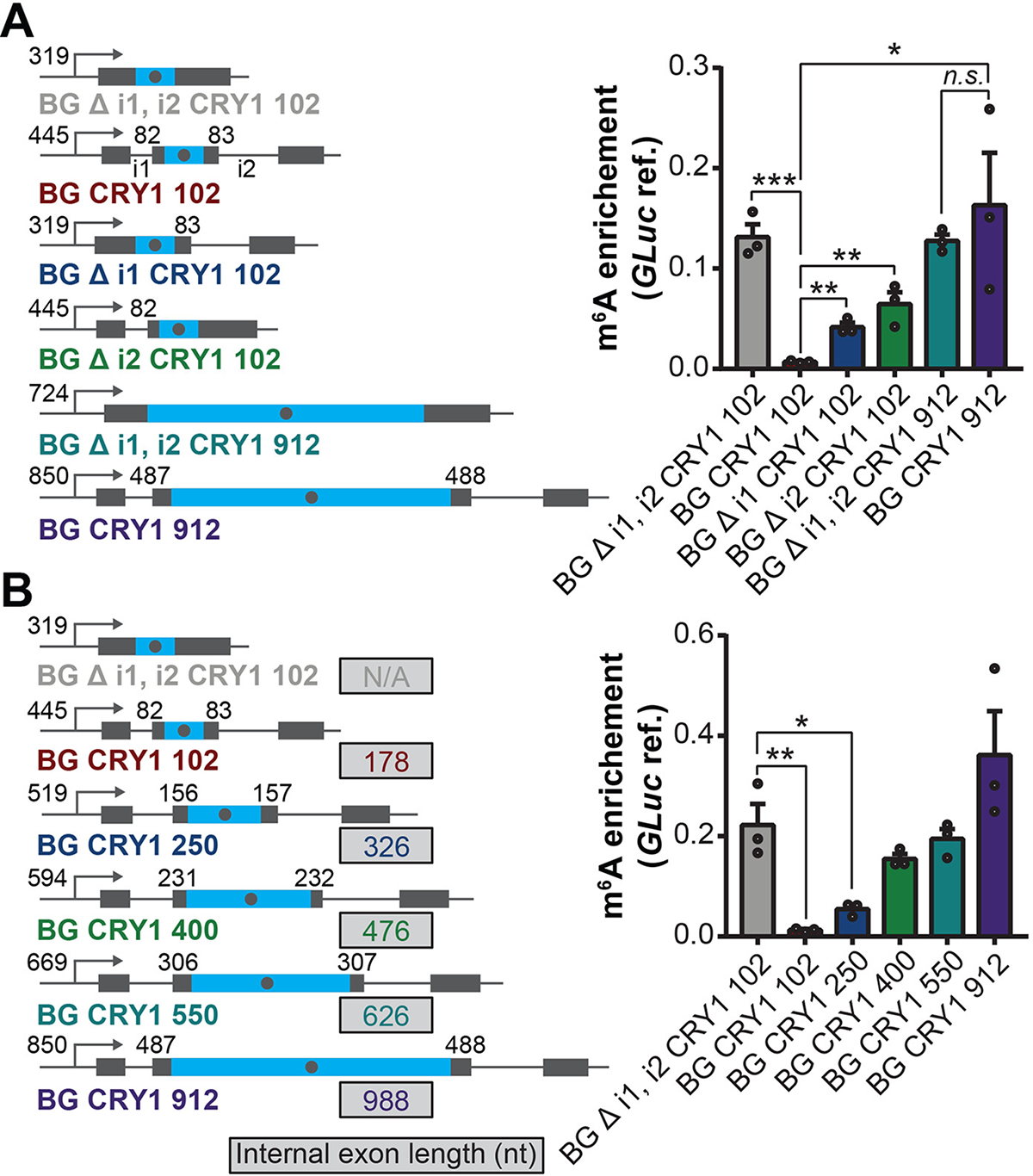
Pre-mRNA splicing suppresses m^6^A methylation in average-length exons. (**A** and **B**) Left: schematic of specified BG CRY1 constructs. Blue regions indicate sequences derived from the *CRY1* endogenous sequence, gray regions indicate sequences derived from rabbit beta-globin (BG). Number following CRY1 refers to the number of nucleotides of exonic sequence surrounding the *CRY1* suppressed m^6^A site in the *CRY1* endogenous mRNA that was cloned into the BG construct. Grey dot in the blue region denotes the suppressed m^6^A site; the number at the left and right of the m^6^A site shows the distance (nt) between the m^6^A site and the 3′ and 5′ splice site, respectively; the number next to the TSS shows the distance (nt) between the m^6^A site and the promoter. Δ denotes deletion of the specified intron(s). Details of each construct are described in the supplementary method. Right: m^6^A enrichment at a *CRY1* suppressed m^6^A site. Primers amplifying a 62 nt-fragment containing the *CRY1* suppressed m^6^A site. m^6^A enrichment was calculated as IP/input normalized to m^6^A-marked *Gaussia* luciferase RNA spike-in IP/input. Mean ± SEM, two-tailed *t*-test, *P < 0.05; **P < 0.01, ***P < 0.001. Three biological replicates.

We next examined the relative enrichment of binding sites for 120 RBPs near endogenously methylated versus suppressed m^6^A sites to identify candidate suppressors (13). Several spliceosome components (*BUD13*, *SF3B4*, *EFTUD2*) were significantly enriched near suppressed sites, suggesting that splicing may suppress m^6^A (fig. S6A). Because suppressed m^6^A sites in CDS primarily reside within average-length internal exons, we hypothesized that the splicing of average-length internal exons may suppress m^6^A methylation. To test this, we cloned a suppressed m^6^A site from an average-length internal exon in the *CRY1* gene (fig. S7A) into a rabbit beta-globin minigene reporter (BG), as well as a version with the introns removed (BG Δi1,i2). First, we cloned the suppressed *CRY1* site and 50/51 nt of flanking sequence into the internal exon, or in the last exon of these constructs. Notably, the spliced construct strongly suppressed methylation of the sequence placed within the internal exon, but not within the last exon (fig. S7B). Removal of either intron (BG Δi1 CRY1 102, BG Δi2 CRY1 102) resulted in partial loss of suppression, indicating that splicing of both introns contributed to methylation suppression (Fig. 2A). Consistent with this notion, deletion of all splice sites also resulted in a decrease in m^6^A suppression (fig. S7C). Cloning in 912 nt of the *CRY1* exonic sequence surrounding the suppressed site into the internal exon (BG CRY1 912), forming a long internal exon, resulted in a loss of suppression (Fig. 2A). We hypothesized that the suppression is dependent on the proximity of the m^6^A site, located within the center of the exon, to splice sites. Expanding the length of the BG CRY1 102 internal exon by cloning in larger amounts of *CRY1* flanking exonic sequence resulted in a progressive loss of suppression, with a > 476 nt internal exon unable to suppress m^6^A (Fig. 2B and fig. S7D). These results reveal a causal role for pre-mRNA splicing in m^6^A regulation.

## Exon junction complexes control m^6^A epitranscriptome specificity

We next sought to understand the mechanism by which splicing suppresses m^6^A deposition. Exon junction complexes (EJCs) are deposited by spliceosomes onto mRNA ~24 nt upstream of exon-exon junctions and plays multifaceted roles in gene expression regulation (14, 15). Notably, two recent studies reported that EJCs efficiently block splicing at proximal aberrant splice sites (16, 17). Additionally, EJCs, together with interacting serine and arginine-rich (SR) proteins, package and compact mRNA and can protect long stretches of proximal RNA from nuclease accessibility in vitro, and also block 5′ to 3′ exonuclease degradation in vivo (18, 19). We reasoned that suppressed m^6^A sites within average-length internal exons are within relatively close proximity to both an upstream and downstream EJC. Conversely, m^6^A sites within long internal exons and near stop codons (which generally reside in long last exons) can be hundreds of nucleotides away from the nearest EJC. We therefore hypothesized that EJCs could mediate the splice site-proximal suppression of m^6^A we observed.

**Fig. 3.**
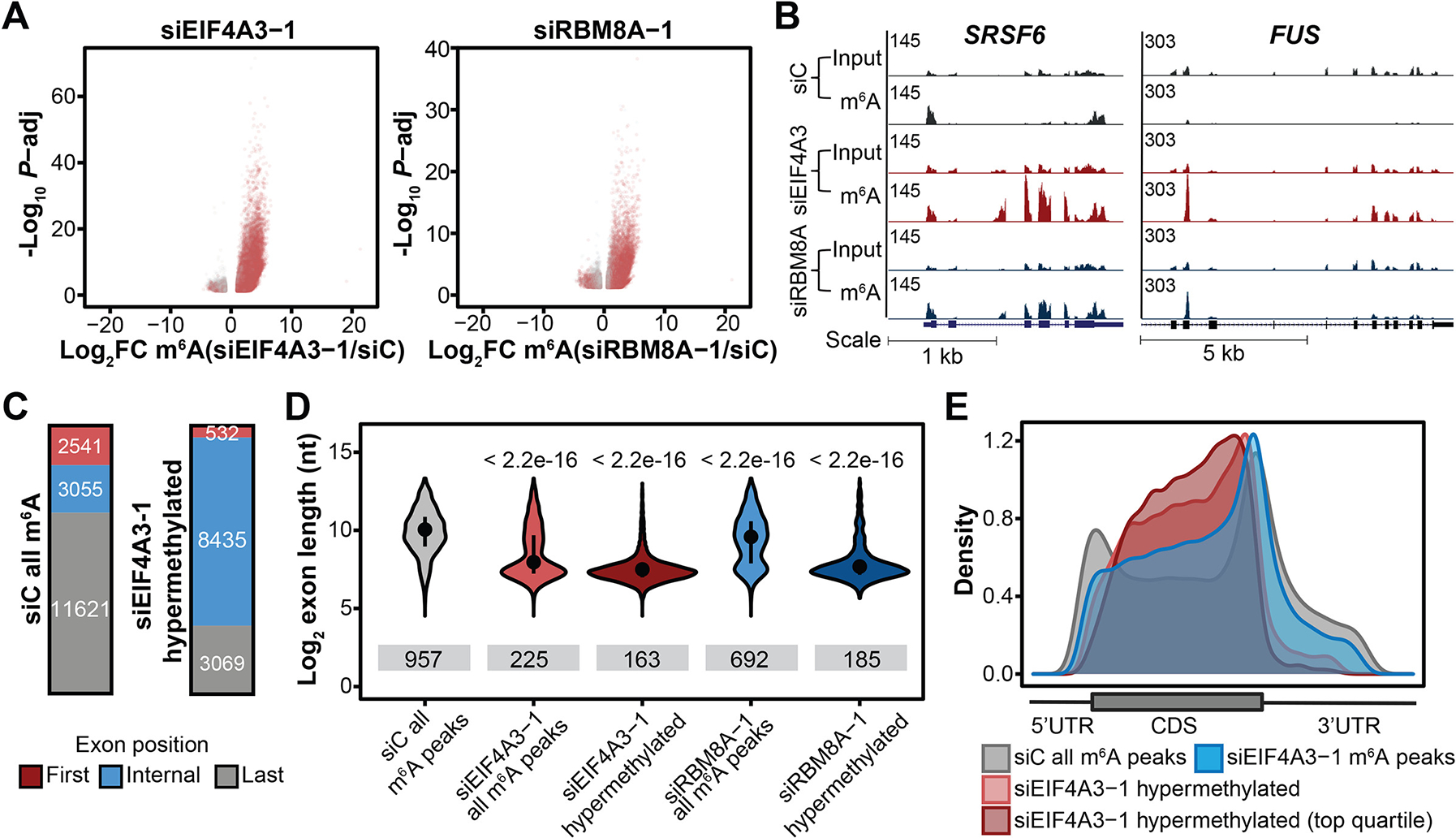
EJCs protect exon junction-proximal RNA in average-length exons within CDS regions from m^6^A methylation. (**A**) Differentially methylated regions upon *EIF4A3* KD (left) and *RBM8A* KD (right) in HeLa cells (FDR<.1, |log_2_FC|>1). Three biological replicates. Gray and red dots indicate differentially methylated regions that overlap and do not overlap m^6^A peaks in the control cells, respectively. (**B**) Input and m^6^A-IP read coverage at *FUS* and *SRSF6* in *EIF4A3* KD, *RBM8A* KD, and control HeLa cells. (**C**) Numbers of *EIF4A3* KD hypermethylated regions (left) and m^6^A peaks in control cells (right) that reside within first, internal or last exons (**D**) Exon lengths for m^6^A peaks residing within internal exons in control KD, EIF4A3 KD and RBM8A KD cells, and exon lengths of hypermethylated regions residing within internal exons in EIF4A3 and RBM8A KD cells. Dot and bar represent median and interquartile range, Wilcoxon rank sum test of indicated group vs. siC all m^6^A peaks. Sample size for each violin plot from left to right is: *n* = 3166, *n* = 6659, *n* = 8438, *n* = 3817, and *n* = 3827. (**E**) Metagenes of m^6^A peaks and significantly hypermethylated m^6^A regions (and top quartile) in *EIF4A3* KD HeLa cells, and m^6^A peaks in control cells.

We knocked down (KD) the core EJC factor EIF4A3 in HeLa cells and assessed the effect on m^6^A deposition transcriptome-wide using m^6^A-MeRIP-seq. Notably, 24,350 regions were significantly hypermethylated upon *EIF4A3* KD, while 3,140 regions were hypomethylated (Fig. 3A). 39% of these hypermethylated regions exhibited a greater than 8-fold increase in m^6^A enrichment compared to the non-targeting siRNA control. We knocked down RBM8A, another core EJC factor (20), and observed similar, though relatively milder, transcriptome-wide m^6^A changes, with 14,034 significantly hypermethylated regions observed, of which 57% overlapped with hypermethylated regions observed in *EIF4A3* KD (Fig. 3A and fig. S8, A and B). The relatively milder m^6^A changes upon *RBM8A* KD may result from relatively lower KD efficiency (table S1) or may indicate a stronger requirement of EIF4A3 for suppression. Concordant with these transcriptome-wide m^6^A changes, using UHPLC-QQQ-MS/MS, we found that *EIF4A3* KD increased global levels of m^6^A in polyadenylated RNA by two-fold, while *RBM8A* KD resulted in a ~25% increase (fig. S8C).

94% of hypermethylated regions from *EIF4A3* KD and 82% of hypermethylated regions from *RBM8A* KD did not overlap with m^6^A peaks identified under the non-targeting siRNA control conditions, suggesting that these regions contain newly methylated suppressed m^6^A sites (Fig. 3, A and B, and fig. S8D). Indeed, out of 1,024 CDS sequences identified by MPm^6^A to contain suppressed m^6^A sites, 46% become methylated upon *EIF4A3* and/or *RBM8A* KD, including the *CRY1* suppressed site (fig. S8E), with three selected suppressed sites validated (fig. S8, F and G) (21). Furthermore, *EIF4A3* KD substantially alleviated the previously observed m^6^A suppression within the internal exon of BG CRY1 102 (fig. S8H).

Consistent with our model, newly methylated and hypermethylated regions were highly enriched in average-length internal exons within CDSs (Fig. 3, C to E, and fig. S8, I and J), with transcriptome-wide increases in m^6^A enrichment in exon junction-proximal regions observed (fig.S9, A and B) upon *EIF4A3* or *RBM8A* KD. *EIF4A3* KD disrupted m^6^A epitranscriptome specificity globally, resulting in substantial loss of enrichment of m^6^A peaks in long internal exons and increased density of m^6^A in the CDS relative to the stop codon (Fig. 3, D and E). It was previously reported that the peak of m^6^A density near stop codons on metagene plots can be more precisely visualized as an increased enrichment 150 nt past the start of last exons (6). *EIF4A3* KD resulted in a global increase in m^6^A enrichment < 150 nt past the start of last exons (fig. S9, A to C), indicating that EJC suppression of methylation proximal to last exon-exon junctions is responsible for the characteristic m^6^A peak density near stop codons. While most transcripts exhibited hypermethylation and contained one or more endogenous m^6^A peaks upon *EIF4A3* KD, over a thousand transcripts that ordinarily lack endogenous m^6^A peaks also gained aberrant m^6^A methylation upon *EIF4A3* KD, revealing a major role for EJCs in suppressing m^6^A deposition on the subset of transcripts that ordinarily are not subject to m^6^A regulation (fig. S9, D to F).

The widespread suppression of m^6^A by the EJCs also implies that many m^6^A are deposited following splicing, which we confirmed using pulse-chase metabolic labeling experiments and UHPLC-QQQ-MS/MS (supplementary text and fig. S10). Two genes used in gene therapies for mucopolysaccharidosis type II and spinal muscular atrophy, *IDS* and *SMN*, contain EJC-suppressed m^6^A sites in their mRNAs, respectively. As expected, when these mRNAs were expressed from cDNA constructs, and thus not bound by EJCs, they were significantly hypermethylated relative to the corresponding endogenous mRNAs (fig. S11). Further, lncRNAs that contain three or more exons globally exhibit EJC suppression of m^6^A in internal exons, while those with two or less do not (supplementary text and fig. S12). We depleted *EIF4A3* with a different siRNA in HeLa cells, and knocked down *EIF4A3* in HEK293T cells as well as in a glioblastoma cancer cell line (U87) that is sensitive to EIF4A3 perturbation (22), and observed similar transcriptome-wide m^6^A changes in each case (figs. S13 to S15). Altogether, our results indicate that spliceosomes widely suppress m^6^A methylation via deposition of EJCs that protect proximal RNA from methylation.

## EJCs regulate mRNA stability by suppressing m^6^A methylation

**Fig. 4.**
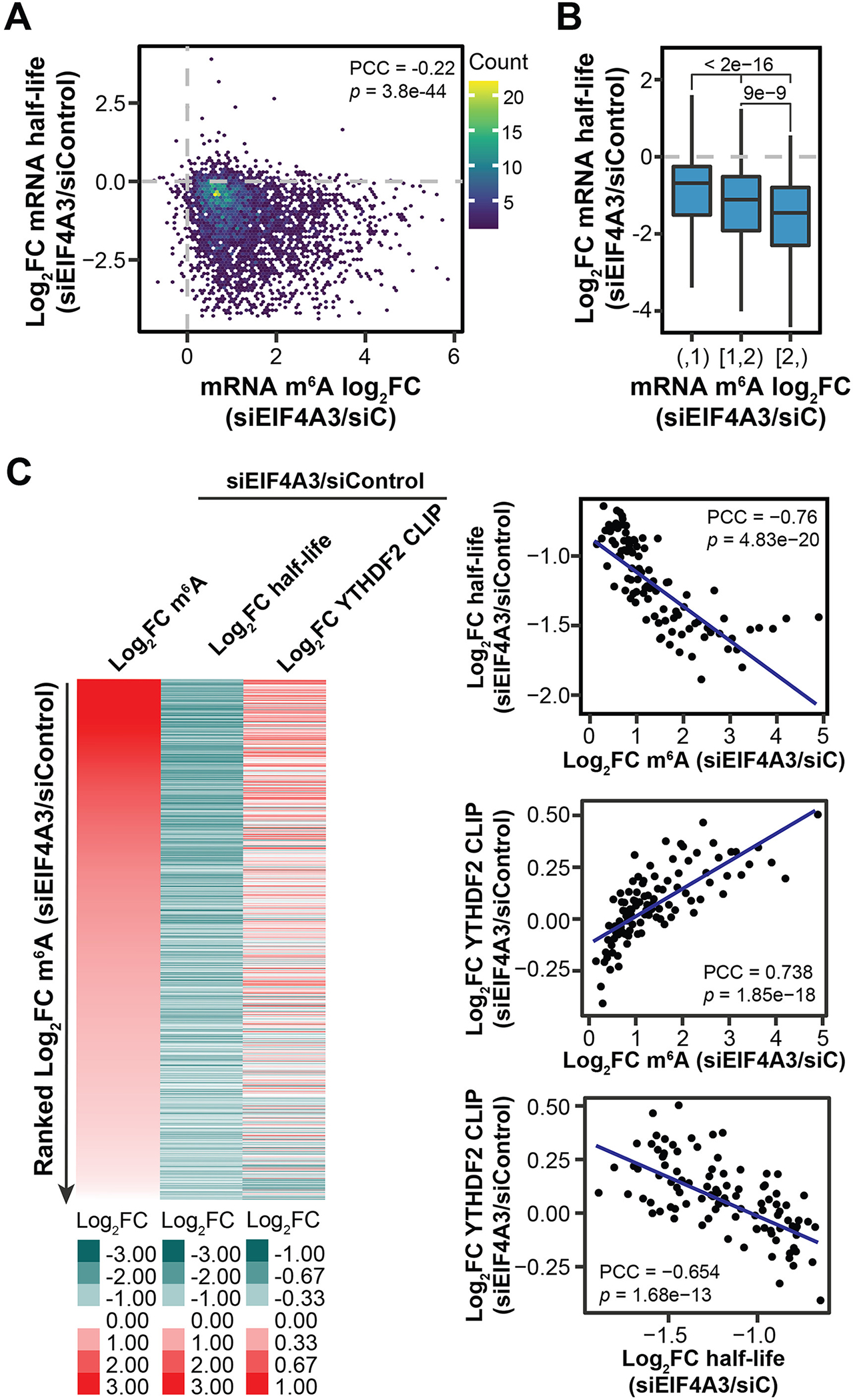
mRNA m^6^A hypermethylation upon EJC depletion destabilizes mRNAs. (**A**) Correlation between fold changes in mRNA half-life and m^6^A level upon *EIF4A3* KD in HeLa cells (*n* = 3840). (**B**) Boxplots showing half-life fold changes of hypermethylated mRNAs upon *EIF4A3* KD in HeLa cells. mRNAs were categorized into three groups according to their methylation changes upon *EIF4A3* KD in HeLa cells. *P* values from Wilcoxon rank sum test. Sample size for each boxplot plot from left to right is: *n* = 1887, *n* = 1201, and *n* = 752. (**C**) Left: heatmap showing fold changes in m^6^A level, mRNA half-life, and YTHDF2 binding upon *EIF4A3* KD in HeLa cells. Right: scatter plots showing the correlation among fold changes in m^6^A level, mRNA half-life, and YTHDF2 binding upon *EIF4A3* KD in HeLa cells. The hypermethylated mRNAs (m^6^A log_2_FC > 0; n = 3424) were categorized into 100 bins based on ranked fold change of m^6^A level upon *EIF4A3* KD. For (A) and (C), PCC and *P* values are shown.

m^6^A is known to mainly accelerate mRNA degradation via the reader protein YTHDF2 (23, 24). Accordingly, we observed globally reduced mRNA half-life of hypermethylated transcripts (~90%) upon *EIF4A3* KD (Fig. 4, A and B). Consistently, we observed generally increased YTHDF2 binding on hypermethylated mRNAs, accompanied with the decreased mRNA half-life (Fig. 4C). *YTHDF2* KD could rescue accelerated degradation of YTHDF2 target transcripts upon *EIF4A3* KD (fig. S16). Further, the density of EJC-loading on transcripts (estimated by the number of exons within CDS regions per 1 kb) correlated with transcriptome-wide mRNA stability (fig. S17). Higher EJC density on transcripts tended to correlate with reduced m^6^A methylation and higher mRNA stability, and the strength of this correlation was diminished by *Mettl3* KO (fig. S17, A and B).

We also found that *METTL3* depletion could generally reduce the expression level changes of hypermethylated genes upon EJC depletion in HeLa cells (supplementary text and fig. S18), indicating that these EJC-dependent gene expression changes are at least in part mediated by m^6^A methylation.

While the vast majority of hypermethylated transcripts were destabilized by *EIF4A3* KD, a small subset of hypermethylated transcripts were stabilized (Fig. 4A). One example is p53, which mediates neurodevelopmental defects in mouse models of EJC haploinsufficiency (25). The *TP53* transcript was hypermethylated but also up-regulated upon *EIF4A3* KD. Mechanistically, we observed increased binding to *TP53* mRNA by IGF2BP proteins, which are known to stabilize methylated transcripts (supplementary text and fig. S19). In summary, while the predominant effect of EJC-mediated m^6^A suppression is to stabilize mRNAs by preventing the YTHDF2-mediated decay, in a minority of instances hypermethylated transcripts can be stabilized by other mechanisms, such as binding by IGF2BPs (26).

Consistent with a general role for m^6^A in promoting translation (12, 27), *EIF4A3* KD led to slightly increased translation efficiency of hypermethylated transcripts, with more highly hypermethylated transcripts exhibiting greater increases in translation efficiency (fig. S20), although the impact was modest relative to the effects observed on mRNA stability.

## Differential m^6^A methylation across tissues and species through EJC suppression

Our model suggests that the cellular EJC levels may impact global mRNA m^6^A deposition in different tissues. Indeed, we observed a negative correlation between *EIF4A3* expression level and global mRNA m^6^A modification level in 25 different human tissues with available transcriptome-wide m^6^A profiles (fig. S21A) (28). We examined the top 10% of genes with the strongest correlations and found that the majority (> 70%) exhibited a negative correlation between m^6^A and *EIF4A3* levels in different tissues. Further, m^6^A levels of these genes also negatively correlated with their transcript abundances (fig. S21B). Similar trends were also observed in mouse tissues (fig. S21C). These results further support m^6^A suppression by EJCs and subsequently mRNA stability regulation in mammalian tissues.

Notably, we observed the lowest *EIF4A3* expressions in brain tissues, which exhibited the highest overall mRNA m^6^A levels (fig. S21A). We further compared the methylome of the human cerebellum (lowest *EIF4A3* level and highest overall mRNA m^6^A) with that of the heart (higher *EIF4A3* level and lower overall mRNA m^6^A). Regions that are hypermethylated in the cerebellum (compared to heart) reside within short internal exons (fig. S21D), suggesting reduced m^6^A suppression due to low *EIF4A3* expression in cerebellum. This association between high m^6^A level and low *EIF4A3* expression in cerebellum was attenuated upon depletion of *METTL3* (fig. S21C). These observations further indicate the widespread suppression by EJCs contributes to tissue-specific m^6^A deposition. We also found that a subset of EJC-suppressed m^6^A sites physiologically escape suppression in certain tissues via methylation of alternative transcript isoforms. These isoforms contain longer exons and thus altered EJC positioning; methylation of these isoforms generates tissue-specific m^6^A patterns (supplementary text and fig. S22).

Lastly, the effect of exon-intron architecture on mRNA stability may have co-evolved with YTHDF2 in vertebrates. The strong correlation between EJC loading, represented by the number of exons, and mRNA level across tissues is maintained across humans, mice, and zebrafish, but not fly and worm, which lack YTHDF2 orthologs (supplementary text and fig. S23).

## EJCs and peripheral EJC factor RNPS1 protect exon junction-proximal RNA regions from aberrant mRNA processing

We did not observe interactions between the methyltransferase complex and EJC complexes (fig. S24), suggesting that steric hindrance from EJCs, rather than a specific inhibitory interaction, accounts for methylation suppression. Nuclear EJCs bound with the peripheral EJC factor RNPS1 multimerize and associate with wide variety of SR and SR-like proteins to package and compact mRNA into higher-order, megadalton-scale mRNPs that ensheathe proximal RNA well beyond the canonical EJC deposition sites (18, 29, 30). Tens to hundreds of nucleotides of proximal RNA could be protected by this mega-complex from nuclease digestion due to this packaging (18, 31). To examine whether the mRNA packaging function of the EJC-mediates suppression of proximal methylation, we isolated EJCs/EJC-bound RNA from cellular extracts, digested away physically accessible RNA with in vitro nuclease treatment, and then measured m^6^A levels on the EJC-protected RNA footprints (fig. S25, A and B). EJC-protected footprints were strongly depleted of m^6^A, indicating that these inaccessible RNA regions are largely protected from m^6^A deposition within cells (fig. S25C). EJCs also protected these footprints from in vitro methylation by recombinant METTL3-METTL14 (fig. S25D). This was not due to general inhibition of methyltransferase activity or lack of methylatable sites on the EJC footprints, as free, unmethylated RNA spiked into the methylation reaction as well as deproteinized footprints were both robustly methylated (fig. S25, D and E). Therefore, EJCs suppress local m^6^A deposition by packaging proximal RNA.

**Fig. 5.**
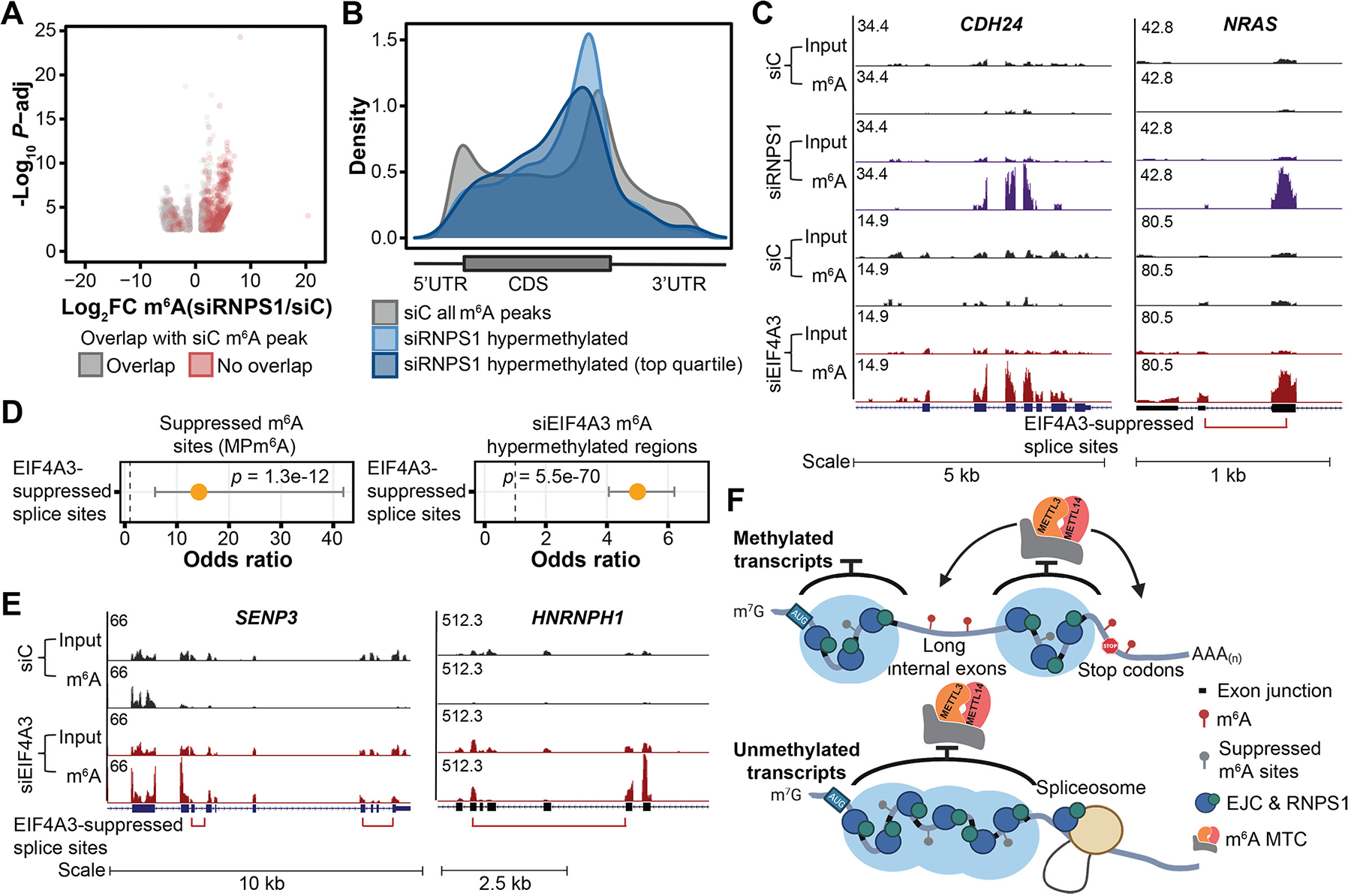
EJCs and RNPS1 protect proximal RNA regions from aberrant mRNA processing. (**A**) Differentially methylated regions upon *RNPS1* KD in HeLa cells (FDR < 0.1, |log_2_FC| > 1), three biological replicates. Gray and red dots indicate differentially methylated regions that overlap and do not overlap with m^6^A peaks in the control KD cells, respectively. (**B**) Metagenes of significantly m^6^A hypermethylated regions (and top quartile) upon *RNPS1* KD in HeLa cells in comparison with that of all m^6^A peaks in control cells. (**C**) Input and m^6^A-IP read coverage at *CDH24* and *NRAS* upon *RNPS1 KD* and *EIF4A3* KD, respectively, as well as corresponding controls in HeLa cells. (**D**) Enrichment of suppressed m^6^A sites (identified from MPm^6^A) at EIF4A3-suppressed splice sites (left) and enrichment of EIF4A3 KD hypermethylated regions at EIF4A3-suppressed splice sites (right). Fisher’s exact test, dot and bar represent odds ratio and 95% confidence interval. (**E**) Input and m^6^A-IP read coverage at *SENP3* and *HNRNPH1* in *EIF4A3* KD and control HeLa cells. (**F**) Schematic model depicting that EJCs and RNPS1 (and potentially other EJC-associated proteins) protect exon junction-proximal RNA from m^6^A deposition through local mRNA packaging. For (C) and (E), red bracket indicates EIF4A3-suppressed splice variant, with ends of bracket indicating the suppressed splice junctions.

We next asked whether the peripheral EJC factor RNPS1, which associates with high molecular weight EJCs in these highly packaged mRNP structures (29), plays a role. *RNPS1* knockdown led to substantial transcript m^6^A hypermethylation within average-length internal exons and CDS regions (Fig. 5, A to C, and fig. S26, A to C). We detected fewer hypermethylated regions overall compared to depletion of the core EJC factors; however, we did observe high overlap (45%) between si*RNPS1* hypermethylated regions and si*EIF4A3*/si*RBM8A* hypermethylated regions (Fig. 5C and fig. S26C). In contrast, depletion of UPF1, a central NMD factor that interacts with the EJC in the cytoplasm, did not result in m^6^A changes similar to those of the core EJC (fig. S27).

The ability of EJCs to protect proximal RNA regions from methylation resembles the recently characterized EJC- and RNPS1-mediated suppression of proximal aberrant splice sites and recursive splicing (16, 17). Transcriptome-wide, EJC-suppressed splice sites significantly colocalize with EJC-suppressed m^6^A sites (supplementary text; Fig. 4, C to E; fig. S26, D to F; and table S2). Altogether, these results suggest that RNPS1-associated EJCs suppress both local cellular m^6^A methylation and splicing through packaging of proximal RNA and point to exon architecture as an important determinant of local RNA accessibility to regulatory machineries. Additionally, beyond components of the m^6^A methyltransferase complex, a number of other RBPs also exhibit preferential binding at long internal exons, suggesting that EJCs may regulate mRNA accessibility to a broader range of mRNA regulators in addition to the splicing and m^6^A methylation machineries through their mRNA packaging function (supplementary text and fig. S28).

## Discussion

Previously identified m^6^A effector proteins fall broadly into three categories according to their activities: “writers”, which catalyze m^6^A methylation, “readers”, which preferentially bind m^6^A, and “erasers”, which reverse m^6^A methylation. Here we establish the EJCs as a member of a new class of m^6^A regulators: “suppressors”, which broadly suppress the deposition of m^6^A (fig. S28). EJCs appear to be a major regulator of m^6^A deposition that mediate multiple key aspects of global m^6^A epitranscriptome specificity, including enrichment of m^6^A in long internal exons, depletion of m^6^A in CDSs and enrichment of m^6^A in last exons near stop codons, and methylation selectivity for transcripts possessing long internal exons. This mechanism may also explain the high abundance of m^6^A on certain non-coding RNAs, such as LINE-1 elements that are generally unspliced and thus not bound by the EJCs (32, 33). Further, our systematic analysis of m^6^A determinants using MPm^6^A may suggest the existence of additional m^6^A suppressing pathways, including m^6^A suppression within the CDS, as *EIF4A3* KD does not appear to completely restore methylation to unspliced levels (supplementary text, fig. S8H, and fig. S29).

Our results point to exon length within transcripts as a functionally relevant element for post-transcriptional gene expression regulation. Mammalian EJCs stably bind the vast majority of pre-translational mRNAs in the transcriptome at closely spaced intervals. Long internal exons and terminal exons, which usually encode UTRs, are notably free of EJCs. This widespread binding, in conjunction with their mRNA packaging function, appears to uniquely position EJCs to broadly determine mRNA accessibility to regulatory machineries, such as the m^6^A methylation and splicing machineries (fig. S30). Our work has relevance for the use of cDNA expression constructs in research studies and gene therapies, as loss of endogenous mRNA exon architecture and EJC protection results in m^6^A hypermethylation (fig. S11), which could modulate gene expression outcome. Finally, our study also suggests that exon length and architecture co-evolved with mRNA processing steps as an additional regulatory layer of gene expression.

## Supplementary Material

SM 1

Sup. Table Captions

Sup. Table 1

Sup. Table 2

Sup. Table 3

Sup. Table 4

Sup. Table 5

Sup. Table 6

mdar reprod. checklist

## Data Availability

Raw and processed data can be found at NCBI GEO accession GSE162199. Custom scripts available on Zenodo (34). All other data are available in the manuscript or the supplementary materials.
