# Supplementary material for: Exon architecture controls mRNA m6A suppression and gene expression
Source: Science. Author manuscript; Available in PMC 2024 Feb 17. (PMC9990141; doi:10.1126/science.abj9090)
Supplement: SM 1 [file NIHMS1874593-supplement-SM_1.pdf]

Science
AAAS

Supplementary Materials for

Exon architecture controls mRNA m^6A suppression and gene expression

P. Cody He *et al.*

Corresponding author: Chuan He, chuanhe@uchicago.edu

DOI: 10.1126/science.abj9090

The PDF file includes:

Materials and Methods
Supplementary Text
Figs. S1 to S30
Captions for Tables S1 to S6
References

Other Supplementary Material for this manuscript includes the following:

Tables S1 to S6
MDAR Reproducibility Checklist

Materials and Methods

Cell lines

HeLa (RRID:CVCL_0030), HEK293T (RRID:CVCL_0063) cell lines used in this study were grown in Dulbecco's modified Eagle's medium (Gibco, 11995040) and supplemented with 10% fetal bovine serum (FBS) (Gibco), and 1% penicillin–streptomycin (Gibco). TREx FLAG-Magoh and TREx FLAG-EIF4A3 cell lines were grown in Dulbecco's modified Eagle's medium (Gibco, 11965126) and supplemented with 10% fetal bovine serum (FBS) (Gibco), and 1% penicillin–streptomycin (Gibco). Cell lines were maintained at 37°C with 5% CO_2. Cells were passaged at ~90% confluency every 2–3 days. Cells were tested for mycoplasma by PCR (*35*). The identity of HeLa and HEK293T cell lines were authenticated with STR profiling (ATCC). HeLa *METTL3*$^{mut/-}$ cells were generated as described in (*36*). TREx FLAG-Magoh and TREx FLAG-EIF4A3 cell lines were gifts from Dr. Guramrit Singh and were generated as described (*18*).

Antibodies

The primary antibodies were purchased from commercial sources, with information about the antibodies and their use in this study listed in table S6.

Oligonucleotides

All individual oligonucleotides in this study were synthesized by IDT, with sequences listed in table S6.

Molecular cloning

pAc-GFP-Hyg-C1 A1335T was generated from pAc-GFP-Hyg-C1 (Clontech) by site-directed mutagenesis using the QuikChange Site-directed mutagenesis kit (Agilent). This A to T substitution at position 1335 disrupts a DRACH motif predicted to be an m^{6}A methylation site (*37*). The pTBG plasmid was a gift from Dr. Ligang Wu (*24*). pBG was constructed by cloning the BG insert from pTBG into the pAc-GFP-HYG-C1 backbone. pBG Δi1, i2, pBG Δi1, and pBG Δi2 and pBG Δss were constructed by cloning synthesized gBlocks (IDT) into the pAc-GFP-Hyg-C1 backbone with PCR amplification to add complementary sequences and assembly with NEBuilder HiFi DNA assembly master mix. *CRY1, FAF1, MLF2*, *MDM2, MAK16,* and *SNTB2* suppressed m^{6}A site sequence with varying amounts of flanking sequence was cloned into BG plasmids by synthesis of oligonucleotide or Gblock (IDT), PCR amplification to add complementary sequences and assembly with AccI and BamHI-HF (NEB) digested plasmids with NEBuilder HiFi DNA assembly master mix. The endogenous promoters for these genes were identified using the Broad ChromHMM annotations (*38*). A forward primer was designed to amplify starting around 500 bp upstream of the identified promoter and a reverse primer sequence was designed targeting a region after the predicted TSS but before the 1st splice site or start codon. The promoter region was PCR amplified from purified HeLa genomic DNA, then inserted into the BG plasmid containing the corresponding insert *via* USER cloning to replace the CMV promoter sequence. pcDNA3.1+ SMN1 Myc His was a gift from Dr. Gideon Dreyfuss. pcDNA3.1+ EV was generated by PCR amplification add complementary sequences and assembly with NEBuilder HiFi DNA assembly master mix. Cloning products were transformed into NEB Turbo Competent E. coli, High

Efficiency (NEB). All cloning primers can be found in table S6. Description of plasmids can be found in table S6.

Design of BG CRY1 constructs

For specified BG CRY constructs shown in Fig. 2, number following CRY1 in the construct name refers to the number of nucleotides of exonic sequence surrounding the *CRY1* suppressed m6A site in the *CRY1* endogenous mRNA that is cloned into the BG construct. BG CRY1 102, BG Δ i1,i2 CRY1 102, BG Δi1 CRY1 102, and BG Δi2 CRY1 102 all contain 102 nt of endogenous CRY1 sequence surrounding the suppressed m^6A site within their internal exons. BG CRY1 102 contains both introns, BG Δ i1,i2 CRY1 102 lacks both introns, and BG Δ i1 CRY1 102 and BG Δ i2 CRY1 102 lack the first and second introns, respectively. BG CRY1 912 and BG Δ i1,i2 CRY1 912 contain 912 nt of endogenous CRY1 sequence surrounding the suppressed m6A site within their internal exons. BG CRY1 912 contains both introns while BG Δ i1,i2 CRY1 912 lacks both introns. BG CRY1 250, BG CRY1 400, BG CRY1 550 contain 250, 400, and 550 nt of endogenous *CRY1* exonic sequence surrounding the suppressed m^6A site within their internal exons, respectively.

siRNA knockdown

Dharmacon ON-TARGET Plus siRNAs were used for siRNA knockdown, with sequences listed in found in table S6. Cells were seeded into TC-treated culture dishes (Corning) 16–20 h before transfection to yield ~30% confluency on the day of transfection. siRNA was transfected at 20 nM concentration with Lipofectamine RNAiMAX (Invitrogen) per the manufacturer's protocol. Cells were incubated with siRNA for 5 hours and then media was replaced with fresh media. Cells were collected 48 hours post-transfection. siRNA sequences can be found in table S6.

Plasmid transfection

Cells were seeded into tissue culture-treated dishes (Corning) 16–20 h before transfection to yield ~80% confluency on the day of transfection. Plasmids were transfected using Lipofectamine 2000. For every μg of plasmid transfected, 3 μl of Lipofectamine 2000 were used. For MPm^6A library transfections, 10 μg of library was transfected into 80% confluent 10 cm plates of HeLa cells, and cells were collected 24 hours later. For pBG transfections, 5 μg of library was transfected into 80% confluent 10 cm plates of HeLa cells. Cells were incubated with plasmid/lipofectamine mixture for 4 hours and then media was replaced with fresh media. Cells were collected 24 hours post-transfection.

RNA isolation

For MPm^6A, m^6A-seq, m^6A-IP-RT-qPCR and SELECT, total RNA was isolated by adding TRIzol reagent directly to cell culture plates (Invitrogen), followed by chloroform extraction (5:1 Trizol:Chloroform), precipitation of RNA from the aqueous phase with isopropanol (2:1 original volume of Trizol:Isopropanol), washing of the RNA pellet with 80% ethanol, and then resuspension in RNase-free water. RNA concentration was measured by ultraviolet absorbance at 260 nm using a Nanodrop instrument. Polyadenylated RNA was purified from total RNA using

the Dynabeads mRNA purification kit (Invitrogen), with the following modifications. Lysis Binding Buffer from the Dynabeads Direct mRNA purification kit (Invitrogen) was used instead of Binding Buffer, and the initial incubation of mRNA at 65°C was omitted. mRNA was eluted in nuclease-free water and concentration was measured by ultraviolet absorbance at 260 nm using a Nanodrop instrument. RNA samples used for RT-qPCR were isolated by using a Direct-zol RNA miniprep kit (Zymo Research) with the optional on-column DNase-I digestion step.

m^6A-meRIP-seq

Polyadenylated RNA was isolated as described above. RNA was adjusted to 10 ng/μL in 100 μL H_2O, 2 μL of 100 pM GLuc m^6A methylated RNA (NEB) transcribed in the presence of 20% m^6ATP and 80% ATP and 2 μL of 100 pM CLuc (NEB) unmethylated RNA was spiked in, and RNA was sheared using a Bioruptor Ultrasonicator (Diagenode), with 30 cycles of 30 seconds on/off at 4°C to generate ~150 nt fragments. 5% of RNA was saved as input and the remainder underwent m^6A-immunoprecipitation (m^6A-IP) using the EpiMark N^6-Methyladenosine Enrichment Kit (NEB). SUPERase-in RNase inhibitor was added to all buffers for a concentration of 20 U/mL. Eluted RNA was purified using RNA Clean and Concentrator (Zymo Research). Library preparation of input and IP samples was performed with the Illumina Truseq Stranded mRNA Library Prep kit. Sequencing was carried out on an Illumina HiSeq4000 machine with SE 50 bp reads.

mRNA lifetime-seq

EIF4A3 was knocked down in HeLa cells as described above. Six 6-cm plates of siControl and *siEIF4A3* HeLa cells were seeded and controlled to afford the same number of cells, respectively. After 48 h, actinomycin D was added to 5 μg/mL at 6 h, 3 h, and 0 h before trypsinization collection. Total RNA was purified by TRIzol reagent (Thermo Scientific). External RNA Control Consortium (ERCC) RNA spike-in control (Ambion) was added proportional to total RNA to each sample before polyadenylated RNA isolation. Library preparation was performed using the Stranded mRNA Library Prep Kit (Illumina) following the manufacturer's protocols. Sequencing was carried out on an Illumina NovaSeq6000 machine with PE 50 bp reads.

CLIP-seq

EIF4A3 was knocked down in HeLa cells as described above. 20 plates of HeLa cells in 15 cm style dish for each replicate of siControl and *siEIF4A3* samples were irradiated twice with 400 mJ/cm^2 at 254 nm in Stratalinker on ice. After irradiation, cells were harvested with a scraper, transferred to a microtube, pelleted at 4000 rpm for 3 min at 4°C, and then lysed in lysis buffer (150 mM NaCl, 0.5% NP-40, 50 mM Tris-HCl (pH 7.5), 2 mM EDTA) with cOmplete, Mini, EDTA-free Protease Inhibitor Cocktail and SUPERase In at 4°C for 1 hour. Subsequently, the lysis mixture was centrifuged at 17,000 g at 4°C for 30 min and the supernatant was carefully collected. The samples were then treated with RNase T1 (1 u/μL) at RT for 15 min and centrifuged to collect the supernatant, 10% of which was saved as input. Then YTHDF2 antibody-conjugated protein G beads prepared by incubating antibody and beads at 4°C for 6 hours were added into the samples and incubate overnight. After beads washing, 10 u/µl RNase T1 was added and incubated at RT

for 8 minutes. After incubation, the beads were extensively washed and then resuspended in 50 µL of SDS-PAGE loading buffer and heated at 95°C for 5 min. The RNA was finally extracted by cutting and recovering the band of YTHDF2-RNA complex from the SDS-PAGE gel. Before library preparation, end-repair by PNK and ATP was performed for the input and IP RNAs. The libraries for both input and IP samples are prepared using NEBNext® Multiplex Small RNA Library Prep Set for Illumina® (NEB, E7300S). Sequencing was carried out on an Illumina NovaSeq6000 machine with PE 50 bp reads.

RiboLace-seq

EIF4A3 was knocked down in HeLa cells as described above. Two 6-cm plates of siControl and *siEIF4A3* HeLa cells were seeded and controlled to afford the same number of cells, respectively. Active ribosome-protected mRNA fragments (RPFs) from each sample were pulled down using RiboLace Module 1 Kit (IMMAGINA) per the manufacturer's protocol. 10% of the cell lysate was saved as input and the polyadenylated RNA was isolated for library preparation using the Stranded mRNA Library Prep Kit (Illumina) following the manufacturer's protocols. The RPFs were finally extracted by cutting and recovering the band between 25-35 nt on the TBE-UREA PAGE gel. Before library preparation, end-repair by PNK and ATP was performed for RPFs. The libraries for both input and IP samples are prepared using NEBNext® Multiplex Small RNA Library Prep Set for Illumina® (NEB, E7300S). Sequencing for both input and RPFs was carried out on an Illumina NovaSeq6000 machine with PE 50 bp reads.

m^{6}A-meRIP-seq peak calling and differential methylation analysis

HISAT2 (version 2.1.0) (*39*) was used to align the sequence reads to reference genome (hg38) with options -k 1 and --known-splicesite-infile generated by the hisat2_extract_splice_sites.py script with the UCSC hg38 GTF. samtools (version 1.7) was used to generate sort and index bam files. The callPeakBinomial function and reportJointPeak from the MeRIPtools R package (version 0.1.0) was used to call m^{6}A peaks with options min_counts = 15, peak_cutoff_fdr = 0.05, peak_cutoff_oddRatio = 1.5, and joint_threshold = 2. RADAR R/Bioconductor package (version 0.2.1) (*40*) was used to perform differential methylation analysis with options fragmentLength = 150, binSize =50, minCountsCutOff = 10, FDR cutoff = .1, log2FC cutoff = 1. Results were robust to varied filtering cutoff levels. Significantly differentially methylated regions were annotated to the RefSeq database (hg38). ClusterProfiler (version 3.6) was used to carry out GO term enrichment analysis (*41*). Metagene profiles were constructed with MetaPlotR. The center position of peaks was used for plotting. If the peak spanned multiple exons and the center of the peak fell in an intron, the end position of the peak was used for plotting. UTRs were scaled to reflect the average size of UTRs in the transcriptome. Bedgraph files for the forward and reverse strand were created using deepTools (version 3.1.3) bamCoverage with options --binSize 10, --normalizeUsing CPM, --exactScaling, --filterRNAstrand forward/reverse. Bedgraph files were visualized using the UCSC genome browser (*42*). To visualize enrichment of input and m^{6}A IP read density surrounding splice sites, deepTools ComputeMatrix and Plotprofiles were used with splice sites obtained from the hisat2_extract_splice_sites.py script and hg38 GTF.

mRNA lifetime-seq data analysis

Raw reads were trimmed by Trimmomatic-0.39 (*43*) to remove low-quality bases and adapters, then aligned to the human genome (hg38), together with ERCC RNA spike-in control (Thermo Fisher Scientific) using HISAT2 (version 2.1.0) (*39*) with '--rna-strandness RF -k 5' parameters. Reads on genes were counted by using HTSeq (*44*) and then normalized to counts per million (CPM). CPM was converted to attomole by linear fitting of the RNA ERCC spike-in. Half-life of RNA was estimated using the mothed described in a previous paper (*32*). Specifically, as actinomycin D treatment results in transcription stalling, the change of RNA concentration at a given time (dC/dt) is proportional to the constant of RNA decay (K_{decay}) and the RNA concentration (C), leading to the following equation:

$$\frac{dC}{dt} = -K_{decay}C$$

Thus, the RNA degradation rate K_{decay} was estimated by:

$$\ln\left(\frac{C}{C_0}\right) = -K_{decay}t$$

To calculate the RNA half-life ($t_{1/2}$), when 50% of the RNA is decayed (that is $\frac{C}{C_0} = \frac{1}{2}$), the equation was:

$$\ln\left(\frac{1}{2}\right) = -K_{decay}t_{\frac{1}{2}}$$

From which we get:

$$t_{\frac{1}{2}} = \frac{ln2}{K_{decay}}$$

CLIP-seq data analysis

Low quality reads were filtered using 'fastq_quality_filter', and adapter were clipped using 'fastx_clipper', then adapter-free were collapsed to remove PCR duplicates by using 'fastx_collapser' and finally reads longer than 15 nt were retained for further analysis (http://hannonlab.cshl.edu/fastx_toolkit/). Reads from rRNA were removed. The preprocessed reads were mapped to hg38 using bowtie (*45*) with '-v 3 -m 10 -k 1 –best –strata' parameters. Reads on each gene were counted using HTSeq (*44*) and then differential binding to genes between knockdown and control were identified by DESeq2 (*46*).

RiboLace-seq data analysis

Raw reads were trimmed by Cutadapt (*47*) to remove low-quality bases and adapters then aligned to the human genome (hg38) using STAR (*48*). Reads on each gene were counted using featureCounts (*49*). Translation efficiency (TE) was calculated as the ratio of ribosome-protected fragments (RPFs) and RNA abundance determined by RiboLace-seq and RNA-seq (*50*) identified by DESeq2 (*46*).

MPm^{6}A Experimental Procedures

MPm^{6}A Oligonucleotide Library Design

59,730 148-nt oligonucleotides were synthesized as a SurePrint Oligonucleotide Library (Agilent). These sequences consist of a 17 nucleotide (nt) 5' universal priming sequence (ACTGGCCGCTTCACTGC), 102 nt of genomic sequence, a 12 nt barcode sequence, a 17 nt 3' universal priming sequence (AGATCGGAAGAGCGTCG). The universal priming sequences were constant for all oligonucleotides and were used to clone the sequences into pAc-GFP-Hyg-C1 A1335T to create a plasmid library, and for Illumina NGS library construction. For "experimental" sequences, the 102 nt genomic sequence consists of an endogenously m^{6}A methylated site or endogenously unmethylated DRACH site, along with 50/51 nucleotides of the flanking genomic sequence for all experimental sequences. If possible, a sequence that does not introduce a stop codon when cloned in-frame was chosen. A minimum read count filter was imposed at the position of the DRACH sequence to filter out DRACH sites that are potentially false-negative m^{6}A sites due to low read coverage. Sequences that did not fall completely within exons were filtered out. Each experimental sequence had a corresponding mutant sequence that served as a negative control to control for potential non-specific enrichment. All "mutant" sequences were identical to experimental sequences but with all RACs mutated to RTT. The 12 nt barcodes were generated using the DNABarcodes R package (*51*). All barcodes that created RAC sequences or microRNA seed sequences (*52*) when assembled into the full oligonucleotide sequence or matched were filtered out.

6897 endogenously methylated sequences were chosen for use in the MPm^{6}A assay. The m^{6}A sites were sampled from m^{6}A sites taken from a published m^{6}A antibody-crosslinking based HeLa single-base resolution m^{6}A dataset (m^{6}A-CLIP) (*53*). To reduce potential false positive m^{6}A sites, we required that the m^{6}A site be present in the cytoplasmic, nuclear and chromatin-associated m^{6}A-CLIP datasets from Ke et al., 2017 and fall within HeLa m^{6}A-seq peaks.

3058 endogenously unmethylated sequences were chosen for use in the assay. To generate the endogenous sequences containing non-methylated DRACH sites, Ensembl hg38 transcriptome cDNA and ncRNA sequences were input into a random forest classifier that predicts m^{6}A sites based on sequence features (*37*). The sequences were sampled from sites predicted to be methylated but which exhibited no cellular methylation in HeLa cells. We implemented a conservative filtering criteria for unmethylated sites, choosing sites that are at least 200 nt away from all m^{6}A-CLIP sites and at least 200 nt away from all HeLa m^{6}A-seq peaks. We implemented a minimum read coverage cutoff as an additional filter to eliminate potential false-negative m^{6}A sites deriving from low expression levels. Additionally, we only chose sites that exhibited depletion of IP reads compared to input at that site (log_2Fold change < -1) to eliminate any potential lowly-methylated m^{6}A sites that fall just under the threshold for significance by m^{6}A-seq or m^{6}A-CLIP. Each experimental and mutant sequence were associated with three distinct barcodes to generate three internal replicates per biological replicate.

MPm^{6}A plasmid library construction

The oligonucleotide pool was amplified by emulsion PCR (Micellula DNA Emulsion & Purification (ePCR) Kit, ChimerX) with Herculase II Fusion DNA polymerase (Agilent) and primers that anneal to the 5' and 3' priming regions and add sequences needed for Gibson assembly cloning. Amplified DNA was purified using DNA Clean and Concentrator-5 (Zymo Research). pAcGFP1-Hyg-C1 A1335T was digested with restriction enzymes overnight at 37°C (BclI-HF(NEB) for 3'UTR MPm^{6}A, BstEII-HF(NEB) for CDS MPm^{6}A, Age1-HF (NEB) for 5'UTR MPm^{6}A), and then gel purified with Qiaquick Gel Extraction kit (Qiagen) to remove contaminating uncut plasmid. ePCR-amplified sequences were cloned by Gibson Assembly with NEBuilder HiFi DNA assembly master mix (NEB) to generate a library of reporter constructs. The assembly reaction was incubated for 1 hour at 50°C, then purified with DNA Clean and Concentrator-5. Restriction sites used for cloning were destroyed by successful assembly. To eliminate any residual uncut plasmid, DNA was isolated and treated with the original restriction enzyme used for cloning and Exonuclease V (RecBCD) (NEB). The DNA was purified using DNA Clean and Concentrator-5 and then 1 μl of eluted DNA was transformed via electroporation into 10 NEB10-beta tubes, using the high efficiency transformation protocol. Transformed cells were spread on 10 15 cm LB agar petri dishes and grown at 37°C overnight. Dilutions were also plated to estimate library coverage. The following day, colonies were scraped into LB and then plasmid DNA was purified with two Zymopure Maxiprep (Zymo Research) columns. Library quality was checked by Sanger sequencing of the plasmid library as well as individual clones that were not pooled into the library.

Cellular MPm^{6}A experimental procedure

10 μg of the MPm^{6}A plasmid library was transfected into an 80% confluent 10 cm plate of HeLa cells for cellular transcription and m^{6}A methylation. 24 hours post-transfection, polyadenylated RNA was isolated as described above. Polyadenylated RNA was incubated with 2U of Turbo DNAse in a 50 μl reaction for 37°C for 1 hour to eliminate plasmid DNA. RNA was adjusted to 10 ng/μL in 100 μL H_2O, 2 μL of 100 pM GLuc m^{6}A methylated RNA transcribed in the presence of 20% m^{6}ATP and 80% ATP and 2 μL of 100 pM CLuc unmethylated RNA was spiked in. 5% of RNA was saved as input and the remainder underwent m^{6}A-immunoprecipitation (m^{6}A-IP) using the EpiMark N^6-Methyladenosine Enrichment Kit (NEB). cDNA was generated using Superscript IV (Thermo) and a site-specific RT primer (Universal Primer 2) that hybridizes to the 3' universal priming sequence. Next generation sequencing libraries were prepared from cDNA by PCR with NEBNext Ultra II Q5 master mix (NEB) using the recommended NGS library PCR cycling conditions. PCR primers that anneal to the 5' and 3' universal priming sequences and add Illumina adaptor sequences were used for library PCR. PCR amplification cycles were determined by qPCR by calculating the number of cycles required for ½ max fluorescence. To control for PCR bias across samples, all input libraries were amplified the same number of cycles, and all IP were amplified the same number of cycles. Library quality was checked by Agilent Bioanalyzer. Libraries were sequenced on an Illumina HiSeq4000 with SE 50 bp reads.

In vitro MPm^{6}A experimental procedure

A T7 promoter sequence was added upstream of the TSS through emulsion PCR of the MPm^{6}A library with primers that anneal to the TSS and cleavage/polyadenylation site. The MPm^{6}A library was in vitro transcribed (IVT) using NEB HiScribe T7 High Yield RNA Synthesis Kit. RNA was incubated with 2U of Turbo DNAse in a 50 µl reaction for 37°C for 1 hour to eliminate plasmid DNA. The purified IVT library was incubated with recombinant METTL3/METTL14. The in vitro methyltransferase reaction was carried out in a 25 µl reaction containing the following components: 1.5 µg IVT RNA, 300 nM recombinant METTL3/METTL14 complex (Active Motif), 1.6 mM SAM, 80 mM KCl, 1.5 mM MgCl2, 0.2 U µl−1 SUPERase-IN RNase inhibitor (Invitrogen), 10 mM DTT, 4% glycerol and 20 mM Tris-HCl (pH 8.0). Mock treatments omitted recombinant METTL3/METTL14. For mock-treated RNA, all reaction components except for the recombinant METTL3/METTL14 complex were added. The reaction was incubated for 22 h at 16 °C; RNA was purified with a Monarch RNA cleanup kit (NEB). A portion of RNA was digested with nuclease P1 and alkaline phosphatase for LC-MS/MS detection to check m^{6}A methylation levels. The remaining RNA was immunoprecipitated with an anti-m^{6}A antibody and further processed as described in the cellular MPm^{6}A method.

MPm^{6}A computational procedure

Reads were mapped back to a reference genome containing the designed sequences with HISAT2 (version 2.1.0), with option -k 1. Counts for each sequence were calculated with samtools (version 1.7) idxstats and sequences with at least 10 counts for all barcoded experimental and mutant sequences were retained. IP/input ratios are calculated for each sequence, representing the m^{6}A enrichment level that is present on each sequence. To calculate an m^{6}A enrichment score for each experimental sequence, we took the average of the IP/input ratios of the three experimental sequence internal replicates and three mutant sequence internal replicates over three or four biological replicates. We then took the difference of the experimental and mutant negative control sequence to calculate the enrichment score. Statistical significance of enrichment was determined with the Wilcoxon Rank Sum test with the Benjamin-Hochberg procedure to control FDR.

Exon length analysis

For exon length analysis, first, internal and last exons were obtained from the TxDb.Hsapiens.UCSC.hg38.knownGene R/Bioconductor package (version 3.4.0). For exon length analysis, only m^{6}A peaks that unambiguously reside within first, internal or last exons were included for analysis. If an m^{6}A peak overlapped multiple exons from different transcript isoforms, the mean length of the exons was used. We determined the exon length for each m^{6}A peak using Bedtools (version v2.25.0) intersectBed and mergeBed. For the RBP internal exon length analysis, a minimum read coverage filter using K562 RNA-seq dataset (*54*) was imposed to filter out intronic binding sites that overlap internal exons from transcript variants not expressed in K562. Results were robust to varied filtering cutoff levels. Statistical significance was determined with the Wilcoxon rank sum test.

RT-qPCR

RT-qPCR was used to assess the relative abundance of mRNA. Total RNA or purified mRNA was reverse transcribed with Primescript Reverse transcriptase (Takara) using poly(dT) primers and random 6 mers to obtain complementary DNA. FastStart Essential DNA Green Master Mix (Roche) was used for qPCR reactions, with primers added to 5 μM. qPCR assays were performed on a LightCycler® 96 Instrument (Roche). qPCR was run using the following conditions: 95°C, 10min; (95°C, 20s; 60°C, 20s; 72°C, 20s) × 40 cycles. Primers used for RT-qPCR can be found in table S6.

m^6A-IP-RT-qPCR

For m^6A-IP-RT-qPCR, the m^6A-IP procedure used in the m^6A-seq was followed until the library preparation step, then input and IP RNA were used for RT-qPCR as described above. m^6A enrichment is calculated by taking the IP/input ratio for the gene of interest, to control for expression level, and then normalizing to the IP/input ratio for a reference RNA, to control for IP efficiency. An m^6A-methylated Gaussia luciferase mRNA spike in or endogenous transcripts from the genes *EID1* or *CCDC77* was used for normalization to control for IP efficiency, as indicated in the corresponding figures. If the measured transcript was expressed from a plasmid, prior to m^6A-IP, RNA was incubated with 2U of Turbo DNAse in a 50 μl reaction for 37°C for 1 hour and then purified with the RNA Clean and Concentrator-5 kit (Zymo Research).

mRNA lifetime-RT-qPCR

Target protein was knocked down in HeLa cells as described above. Nine 6-cm plates of siControl, *siEIF4A3*, *siEIF4A3* and *siYTHDF2*, and *siEIF4A3* and *siIGF2BP1-3* HeLa cells were seeded and controlled to afford the same number of cells, respectively. After 48 h, actinomycin D was added to 5 μg/mL at 6 h, 3 h, and 0 h before trypsinization collection (three replicates for each time point). Total RNA was purified by TRIzol reagent (Thermo Scientific). External RNA Control Consortium (ERCC) RNA spike-in control (Ambion) was added proportional to total. RT-qPCR were performed as described above.

SELECT

For each sample, 25 ng of total RNA was mixed with 0.8 μL up-probe and down-probe oligonucleotide (1 μM), 1 μL dTTP (100 μM), and 2 μL 10X CutSmart buffer (NEB) supplemented with H_2O to 17 μL total volume. The reaction was incubated at a temperature gradient: 90 °C for 1 min, 80 °C for 1 min, 70 °C for 1 min, 60 °C for 1 min, 50 °C for 1 min, and then 40 °C for 6 min. Subsequently, a 3 μL of enzyme mixture containing 0.5 μL *Bst* 2.0 DNA polymerase (0.02 U/μL) (NEB M0275S), 0.5 μL SplintR ligase (1 U/μL) (NEB M0375S), and 2 μL ATP (5 mM) was added for a final volume of 20 μL. The final reaction mixture was incubated at 40 °C for 20 min then denatured at 80 °C for 20 min. qPCR is used to quantify the abundance of the ligated product. 10 μL 2X FastStart Essential DNA Green Master Mix (Roche) qPCR master mix, 0.8 μL universal SELECT primer (10 μM), 2 μL reaction from previous step, and 7.2 μL H_2O. qPCR quantification of the mRNA levels for the target gene is used to normalize the amount of ligated product for gene expression variation.

Western blot

Cells were lysed in either 2X Laemmli buffer (Biorad) or NP-40 lysis buffer (150 mM sodium chloride, 1.0% NP-40, 50 mM Tris pH 7.5) with 1 × protease inhibitor cocktail (Sigma). Pierce™ BCA Protein Assay Kit (Thermo Fisher) was used to measure protein concentrations. SDS-PAGE was carried out using NuPAGE 4 to 12%, Bis-Tris, 1.5 mm, Mini Protein Gels and buffers (Invitrogen). Proteins were transferred to a .45 µm pore nitrocellulose membrane (Biorad) using a semi-dry transfer apparatus and stained with Ponceau S (Sigma) to check transfer efficiency. Membrane was blocked with PBST (0.1% (v/v) Tween 20 in 1x Phosphate-buffered Saline) with 5% blotting grade blocker (Biorad) for 1 hour at room temperature. Primary antibody with PBST with 2% blocker or BSA and .02% sodium azide was added overnight at 4°C. at dilution listed in table S6. Membrane was washed three times for 5 min in PBST. Secondary antibody conjugated to HRP added in PBST with 2% blocker for 1 hour at 4°C at dilution listed in table S6. Washed 3X5min in PBST. Membrane was washed three times for 5 min in PBST. Membrane was visualized using SuperSignal™ West Dura Extended Duration Substrate (Thermo Scientific).

LC-MS/MS quantification of m^6A in RNA

For each sample, 25 ng of RNA was digested with 1 unit of Nuclease P1 (Sigma) in P1 buffer (20 mM NH4OAc, pH 5.5) in a final reaction volume of 20 µL for 2 hours at 42°C. Subsequently, 1µL of FastAP (Thermo Fisher) and 2.5µL of 10x FastAP buffer were added to each sample, and they were incubated at 37°C for 4 hours. Samples were then diluted with 27µL of water and filtered through a 0.2µm PVDF filter (0.2µm pore size, 0.4mm diameter, Millipore). 5µL of each filtered sample was separated by reverse phase ultra-performance liquid chromatography on a C18 column on an Agilent Technologies 1290 Infinity II liquid chromatography system, followed by mass spectrometry on a Sciex Triple Quad 6500 triple-quadrupole mass spectrometer in positive electrospray ionization mode. Nucleosides were quantified using nucleoside-to-base transitions of 282.101>150.100 (m^6A), 267.966>136.000 (A), and 284.004>152.100 (G). Quantification was performed by comparing with the standard curve obtained from pure nucleoside standards running with the same batch of samples. The concentration of m^6A was normalized to the concentration of A or G in each sample to enable comparisons in m^6A levels between samples.

Nascent RNA m^6A measurement

EIF4A3 was knocked down in HeLa cells as described above. Cells were grown to ~80% confluency in 15 cm plates and 15-20 plates are needed for each replicate at each time point. BrU (Sigma 850187-250MG) was added to cell culture medium at final concentration of 2 mM and incubated for 15 min. Then the cells were washed with PBS twice and chased with fresh media for 0, 15, 30, 45, 60, 90, and 120 min. Total RNA was extracted using TRIzol following the manufacture's protocol. BrU labeled nascent RNA was pulled down with Anti-BrdU antibody (Abcam ab8955) referring to the manufacture's protocol of the EpiMark N^6-Methyladenosine Enrichment Kit (NEB). rRNA was further depleted with using a RiboMinus Eukaryote kit (Thermo Scientific). m^6A/A% of rRNA depleted nascent RNA at each time point with or without *EIF4A3* KD in HeLa cells was measured by LC-QQQ-MS/MS as described above.

Isolation of EJC-protected RNA footprints

EJC-protected RNA footprints were isolated with a procedure adapted from previous reports (*18*, *29*). For isolation of EJC-protected RNA footprints via FLAG-Magoh or FLAG-EIF4A3 immunoprecipitation, TREx-HEK293 cells containing a stable copy of FLAG-tagged Magoh or a FLAG-tagged EIF4A3 were used. TREx-HEK293 cells were grown in four 15-cm plates. Expression of the FLAG-tagged protein was induced with a working concentration of 200 ng/mL tetracycline for ~16 hr. 90 minutes prior to cell harvesting, cycloheximide (CHX) was added to 100 μg/ml. The monolayer was rinsed and harvested in phosphate-buffered saline (PBS) containing 100 μg/ml CHX. The cells were lysed in 4 mLhypotonic lysis buffer (20 mM Tris-HCl pH7.5, 15 mM 11 NaCl, 10 mM EDTA, 0.5% NP-40, 0.1% Triton X-100, 1x Halt Protease Inhibitor (Thermo Fisher), .2 U/μL of SUPERase-In (Thermo Fisher), 100 μg/ml CHX) for 1 hour on ice. The suspension was sonicated (Branson Digital Sonifier-250) at 40% amplitude using a Microtip for a total of 16 seconds (in 2 second bursts with 10 second intervals). NaCl was adjusted to 150 mM and the lysate was cleared by centrifugation at 10,000xg for 10 min at 4°C. The cleared lysate was diluted to 10 mL in the above lysis buffer with final NaCl concentration of 150 mM. The diluted lysate was incubated for 3 hr at 4°C with 1mL of anti-FLAG magnetic beads (50% slurry, Sigma) pre-washed twice with 12 mL IsoWB (Isotonic wash buffer; 20 mM TrisHCl pH7.5, 150 mM NaCl, 0.1% NP-40). The RNA-protein (RNP) complexes captured on beads were sequentially washed four times (4 x 12ml) with ice-cold IsoWB. After the fourth wash, bound RNP complexes were incubated with one bed volume of IsoWB containing 1U/μL of RNase I (Ambion) for 10 min at 37°C shaking at 1200 rpm. RNP complexes were again washed four times with 10 mL IsoWB supplemented with .02 U/μL SUPERase-IN. RNPs were eluted with 40 μL of clear sample buffer (100 mM Tris-HCl pH6.8, 4% SDS, 10 mM EDTA, 100 mM DTT) at 25°C for 5 min, and subsequently at 95°C for 2 min. For isolation of EJC-protected RNA footprints via endogenous EIF4A3 immunoprecipitation, HEK293T cells were harvested and lysed as above, and the cleared lysate was added to 300 μl Protein G Dynabeads pre-coupled to 40 μg of anti-EIF4A3 (Bethyl A302-980A). Immunoprecipitation, RNase I digestion, and elution were carried out as above. After elution, RNA footprints were isolated by Trizol/chloroform extraction and isopropanol precipitation with GlycoBlue coprecipitant (Invitrogen) and then run on a 6% TBE-Urea gel to assess length distribution. m^6A levels were measured by LC-MS/MS as described above.

In vitro methylation of EJC-protected RNA footprints with recombinant METTL3-METTL14

For in vitro methylation of EJC-bound RNA eluted under native conditions, the procedure for isolating EJC-protected RNA footprints from TREx-HEK293 cells expressing FLAG-tagged EIF4A3 (described above) was followed until the denaturing elution step. RNP complexes were eluted under native conditions by incubating beads with 100 μL of elution buffer consisting of 28 mM Tris-HCl (pH 8.0), 112 mM KCl, 2.1 mM $MgCl_2$, and 1 μg/μL FLAG peptide (Sigma) per 250 μL of anti-FLAG 50% magnetic beads slurry used, rotating at 4°C for 2.5 hours. The supernatant containing the eluted RNPs was collected, and DTT, glycerol, SUPERase-IN RNase inhibitor (Invitrogen), SAM (Cayman) and recombinant METTL3-METTL14 (Active Motif) were added up to the following final reaction conditions: 300 nM recombinant METTL3/METTL14 complex, 1.6 mM SAM, 80 mM KCl, 1.5 mM $MgCl_2$, 0.2 U μl^{-1} SUPERase-IN RNase inhibitor,

10 mM DTT, 4% glycerol and 20 mM Tris-HCl (pH 8.0). For methylation reactions containing RNPs with unmethylated RNA spiked in, in vitro transcribed MPm^{6}A library (produced as described above) was added to the reaction to a final concentration of 7 ng/ul, approximately 10-fold the yield of eluted EJC-protected RNA footprints. Reactions were incubated at 16°C for 12 hours, then collected in Trizol and purified by Trizol/chloroform extraction and isopropanol precipitation with GlycoBlue coprecipitant. For in vitro methylation of Trizol purified, deproteinized EJC-protected RNA footprints, EJC-protected RNA footprints were first purified using Trizol/chloroform extraction and isopropanol precipitation with GlycoBlue coprecipitant. The isolated RNA was subjected to the same in vitro methylation conditions as the RNPs eluted under native conditions described above, at 16°C for 12 hours. m^{6}A levels were measured by LC-MS/MS as described above, subtracting background signal from mock reactions containing all components except for RNA.

Protein co-immunoprecipitation

For METTL3 co-immunoprecipitation, 10^7 HeLa cells were lysed in 500 µL of ice-cold lysis buffer (50 mM Tris-HCl pH 7.4, 100 mM KCl, 5 mM MgCl2, .5% NP-40, 1x Halt Protease Inhibitor). Cells were incubated on ice for 5 min. Lysate was then sonicated in a Bioruptor Pico, with five cycles of 30 second bursts at 30 second intervals. Lysates were spun down, 20,000xg for 10 min at 4°C, and a portion was saved as an input sample. 50 µl M280 sheep anti-rabbit beads (Thermo Fisher) was incubated with 4 µg of anti-METTL3 (Abcam, ab195352), or 4µg of rabbit IgG isotype control (Cell Signaling Technology) rotating end over end for 1 hour at room temperature, and washed 6 times in NT-2 wash buffer (50 mM Tris-HCl pH 7.4, 150 mM NaCl, .05% NP-40). Lysate was added to prepared beads and incubated rotating end over end at 4°C for 16 hours. Beads were washed 5 times with ice-cold NT-2 wash buffer, and proteins were eluted by addition of 2X Laemmli buffer and incubation at 95°C for 30 min. Input and IP samples were run on an SDS-PAGE gel and visualized by Western blot, as described above. For EIF4A3 co-immunoprecipitation, HEK293T cells were lysed and EIF4A3 was immunoprecipitated as described for above for isolation of EJC-protected footprints, but with a 16-hour incubation of lysate with beads and antibody, rotating end over end at 4°C. Beads were treated with RNase I as above, or left untreated. Proteins were eluted from beads by addition of 2X Laemmli buffer and incubation at 95°C for 30 minutes. Input and IP samples were run on an SDS-PAGE gel and visualized by Western blot, as described above.

Statistics and reproducibility

At least three biological replicates were used in each experiment unless otherwise stated. Statistics and *P*-values are described in figure legends.

Differential expression analysis

DESeq2 (version 1.18.1) was used to detect differentially expressed genes using a significance threshold of adjusted p-value <.05 per a previously reported procedure (*46*). To identify genes that have a significant change in response to EJC KD in WT vs. METTL3mut cells, the design formula

~ genotype + treatment + genotype:treatment was used. The *P*-value and magnitude of the genotype:treatment interaction term for tested genes was reported.

Human tissue m^6A analysis

m^6A-MeRIP-seq data for human tissues was obtained from Liu et al. 2020 (*28*). As described in (*28*), we used read 2 of each fastq file for alignment. Reads were trimmed with TrimGalore (version 0.6.6) with options --length 30 --clip_R1 3 (*47*). HISAT2 (version 2.1.0) was used to align the trimmed sequence reads to reference genome (hg38) with options -k 1 and --known-splicesite-infile generated by the hisat2_extract_splice_sites.py script with the UCSC hg38 GTF. samtools (version 1.7) was used to generate, sort and index bam files. BigWig files for the forward and reverse strand were created using deeptools (version 3.1.3) bamCoverage with options --binSize 10, --normalizeUsing CPM, --exactScaling, --filterRNAstrand forward/reverse. To assess the methylation status of EJC-suppressed m^6A regions in tissues, we took *siEIF4A3*-1 KD significantly hypermethylated regions from HeLa cells that did not overlap a HeLa siC m^6A peak. We filtered out m^6A peaks from human tissues that overlapped any portion of siC m^6A peaks and filtered out any *siEIF4A3*-1 KD hypermethylated regions that overlapped any HeLa m^6A peak regions from the Liu et al. 2020 dataset to control for batch effects or protocol-specific differences. We then assessed the methylation status of EJC-suppressed m^6A regions in tissues by assessing the overlap of these filtered hypermethylated regions with filtered tissue m^6A peaks. To identify peaks that overlapped exon-intron boundaries we filtered for peaks that spanned splice sites and exhibited m^6A-IP read coverage > 1 CPM in the intron 20 nt away from splice sites. Results were robust to varied filtering cutoff levels. To obtain the number of m^6A-IP reads that mapped to the exon-intron junction, and obtain the m^6A enrichment for peaks, we used the summarizeOverlaps function from the GenomicAlignments R package to count reads. We normalized the read numbers to the total number of mapped reads in each sample and set a minimum input expression filter. We identified intron retention events using IRFinder, with default settings, using a cutoff of IR ratio >.1, per a previously reported procedure (*55*). We obtained polyA sites from PolyA.Site.2.0 (*56*) and gene models from GENCODE v32 comprehensive transcript set.

m^6A-QTL analysis

m^6A-QTLs and corresponding m^6A peaks were obtained from (*12*). Reads were aligned as previously described, and BigWig files for the forward and reverse strand were created using deeptools (version 3.1.3) bamCoverage with options --binSize 10, --normalizeUsing CPM, --exactScaling, --filterRNAstrand forward/reverse. To assess the methylation status of EJC-suppressed m^6A regions in tissues, we took regions that were significantly hypermethylated upon *siEIF4A3*-1 and *siEIF4A3*-2 KD in regions from HeLa and HEK293T cells that did not overlap an siC m^6A peak in HeLa and HEK293T cells, respectively. We filtered out m^6A-QTL m^6A peaks that overlapped with any portion of siC m^6A peaks for HeLa and HEK293T cells. We then assessed whether the filtered m^6A-QTL m^6A peaks overlapped the filtered EJC-suppressed m^6A regions. We obtained polyA sites from PolyA.Site.2.0 (*56*) and gene models from GENCODE v32 comprehensive transcript set.

Enrichment tests

For all enrichment tests, Fisher's exact test was used to assess significance of enrichment. To test enrichment of RBPs at MPm^{6}A endogenously methylated vs MPm^{6}A suppressed m^{6}A sites, 120 K562 RBP eCLIP datasets were obtained from ENCODE (*13*). Peaks with $-\log_{10}$ (PValue) >= 3 and $\log_2$(signalValue) >= 3 in two replicates were used, per the previously reported procedure (*13*). Benjamin-Hochberg procedure was used to control FDR. Only sites that were not differentially expressed more than 5-fold in K562 (using DESeq2) were included in the analysis to filter possible cell-type specific peaks due to differential gene expression. Results were robust to varied filtering cutoff levels. To test enrichment of FTO and ALKBH5 at MPm^{6}A endogenously methylated vs MPm^{6}A suppressed m^{6}A sites, FTO and ALKBH5 binding sites were obtained from (*57*, *58*). To test enrichment of EIF4A3-suppressed suppressed splice sites at MPm^{6}A endogenously methylated CDS m^{6}A sites vs MPm^{6}A suppressed CDS m^{6}A sites, and enrichment of EIF4A3-suppressed suppressed splice sites at *EIF4A3* KD hypermethylated regions vs m^{6}A peak regions that are not hypermethylated, EIF4A3-suppressed suppressed splice sites were obtained from (*16*). 100 nt region proximal to suppressed splice sites was used for enrichment analysis. MPm^{6}A endogenously methylated CDS m^{6}A sites (that remain methylated in the MPm^{6}A reporter) and MPm^{6}A suppressed CDS m^{6}A sites were taken from cellular 3'UTR MPm^{6}A data. *EIF4A3* KD and m^{6}A peak regions that are not hypermethylated were obtained from data from siC and *siEIF4A3*-1 KD in HeLa cells described in Fig 3. m^{6}A peak regions that are not hypermethylated were obtained by taking m^{6}A peaks that do not overlap *EIF4A3* KD hypermethylated regions. To test enrichment of m^{6}A-containing transcripts in transcripts with at least one internal exon > 400 nt *vs* transcripts with all internal exons < 400 nt, constitutive internal exons greater than 400 nt in length were obtained from the HEXEvent database (*59*) with constitLevel > .9. Long internal exons were associated with their respective genes to identify genes that contained at least one internal exon > 400 nt. m^{6}A-methylated genes were defined based on HeLa m^{6}A-seq data. Gene expression levels were calculated using Kallisto (version 0.44.0) (*60*) and an expression cutoff at the lowest quartile of expression for all m^{6}A-methylated genes was used to exclude lowly expressed genes that do not have sufficient read coverage to detect m^{6}A peaks from the analysis. Results were robust to varied filtering cutoff levels.

Supplementary Text

Temporal order of m^{6}A methylation and pre-mRNA splicing

Our results reveal widespread crosstalk between splicing and m^{6}A methylation. m^{6}A has previously been reported to affect splicing (*61–63*), and we now present evidence that, reciprocally, splicing is a widespread regulator of m^{6}A deposition. The widespread suppression of m^{6}A by the EJCs implies that a significant amount of m^{6}A is deposited following splicing. A previous report found that nascent mRNA, which is enriched for unspliced/non-EJC bound pre-mRNA, exhibits increased relative frequency of m^{6}A peaks near splice sites compared to bulk mRNA (*61*). Consistent with our model, further analysis of this dataset reveals that m^{6}A peaks in nascent RNA reside in shorter internal exons than in mRNA and exhibit reduced enrichment near stop codons, which parallels the distribution of m^{6}A in bulk mRNA upon EJC depletion (fig. S10, A and B).

To study the temporal order of splicing and m^{6}A deposition globally, we performed pulse-chase with metabolic labeling experiments using HeLa cells with or without *EIF4A3* KD. We quantified the m^{6}A levels of nascent RNA after rRNA depletion with UHPLC-QQQ-MS/MS (fig.

S10C). We found that m^6A was deposited continuously within the two-hour chase (fig. S10D). Compared to wild-type cells, we observe increased m^6A deposition at all time points for the *EIF4A3* KD samples, with the greatest increases observed for the samples with 45-60 min from the beginning of pulse (fig. S10E). The time scale of splicing and EJC loading of the majority of the transcripts could occur faster than the nascent RNA m^6A deposition rate measured here (fig. S10, D and E) (*64*, *65*), our data thus indicate that a significant portion of m^6A deposition can occur during/after splicing (EJC-dependent) (9), which is consistent with our model.

LncRNAs with three or more exons are also subject to the EJC-mediated m^6A suppression

The EJC-mediated suppression should be active not only on mRNA, but also on other RNA Pol II-transcribed and spliced RNA that are bound by EJCs, such as lncRNAs with multiple exons. Indeed, we found that average-length internal exons of lncRNAs containing three or more exons were more likely m^6A hypermethylated upon *EIF4A3* KD, while no obvious difference was observed for different groups of exons for lncRNAs with less than three exons (fig. S12). This data is consistent with our EJC-mediated m^6A suppression model and indicates a broader impact of this suppression mechanism.

METTL3 depletion generally reduces expression changes of hypermethylated genes induced by EJC depletion

The most significantly enriched gene ontology terms for transcripts containing EJC-suppressed m^6A sites are cell division, splicing, and chromatin modification, which are also cellular processes that are disrupted upon loss of the EJC (*66–68*) (fig. S18A). This suggests that aberrant m^6A hypermethylation upon EJC KD may alter expression of these transcripts and contribute to defects in these pathways.

To test this, we knocked down *EIF4A3* in wild-type and *METTL3* heterozygous knockout (*METTL3*$^{mut/-}$) HeLa cells (*36*) that exhibit reduced global mRNA m^6A level. METTL3 depletion did not affect global EJC loading (fig. S18D). We compared gene expression changes in WT HeLa cells and *METTL3*$^{mut/-}$ cells upon EJC depletion (fig. S17B). *METTL3* depletion could generally reduce expression changes of hypermethylated genes observed upon EJC depletion in HeLa cells (fig. S17, C to E), indicating that these EJC-dependent gene expression changes are at least in part mediated by m^6A methylation.

A small subset of hypermethylated transcripts were stabilized upon EJC depletion

~10% of transcripts with m^6A hypermethylation were stabilized upon *EIF4A3* KD (Fig. 3A). EJC haploinsufficiency within neural stem cells in mice has been shown to cause neurodevelopmental defects, which may model neurodevelopmental disorders in humans that are caused by EJC mutations. p53 is upregulated in EJC haploinsufficient cells and mediates the observed neurodevelopmental defects (*25*). However, the molecular mechanism by which EJC depletion leads to increased p53 levels is unclear. We found that *TP53* mRNA, which encodes p53, is normally unmethylated but gains aberrant methylation upon *EIF4A3* KD (fig. S19A). Further, p53 upregulation upon EJC depletion is disrupted in *METTL3*$^{mut/-}$ cells (fig. S19B); note that the faster

migrating band corresponds to an alternatively spliced, smaller p53 isoform that was previously reported to appear upon EJC depletion (*69*). m^6A sites in the internal exon may facilitate alternative splicing to generate short isoforms of *TP53*, which could be partially rescued by METTL3 depletion (fig. S19C), while METTL3 depletion did not affect global EJC loading (fig. S19D).

These results indicate that EIF4A3 downregulates p53 expression through its effects on m^6A. We next investigated the mechanism by which m^6A methylation upregulates TP53. We found that the hypermethylated region on the *TP53* transcript upon *EIF4A3* KD could recruit more IGF2BPs to *TP53* (fig. S18A); IGF2BP1-3 (IGF2BPs) are proteins known to selectively bind and stabilize m^6A-modified transcripts (*26*). This suggests that IGF2BPs may be involved in stabilizing a subset of hypermethylated RNAs upon *EIF4A3* KD. Indeed, we performed *IGF2BP1-3* KD rescue experiments in EJC depletion cells and validated the mRNA stabilization effect of *TP53* and three other IGF2BPs targets through the IGF2BP-m^6A axis (fig. S19, E and F). Altogether, EJCs impact mRNA stability through m^6A suppression *via* different reader proteins, such as YTHDF2 and IGF2BPs. EJC may also stabilize mRNA in an m^6A-independent manner due to its role in promoting nuclear export, which may decrease degradation of certain transcripts by the RNA exosome. Our result show that loss of EJCs result in increased decay of most hypermethylated transcripts, but a small portion are stabilized, through distinct m^6A readers or other mechanisms.

Differential m^6A deposition in alternative transcript isoforms

To further probe EJC-mediated suppression in human tissues we investigated the methylation status of the EJC-suppressed m^6A regions identified from HeLa cells (Fig. 3A) across human tissues. Indeed, we found that the vast majority (> 95%) of these EJC-suppressed regions identified in HeLa cells were also suppressed *in vivo* (fig. S22A). Most regions were not methylated in any tissue, indicating that EJCs ubiquitously suppress many m^6A sites in a tissue-independent manner, consistent with the conserved m^6A distribution pattern across human tissue types (*28*). While the vast majority of the thousands of EJC-suppressed regions appear ubiquitously suppressed, we noted that hundreds of regions escape suppression in various tissues (fig. S22B). Notably, the methylation status of these regions varies across tissues, suggesting that these regions correspond to tissue-specific m^6A peaks (fig. S22C).

We have shown that *EIF4A3* expression levels across tissues could affect the mRNA m^6A levels (fig. S21, A to C), particularly the m^6A sites residing in shorter internal exons (fig. S21D). Another mechanism by which the EJC-mediated suppression could regulate tissue-specific m^6A deposition is through alternative transcript isoforms, where alternative splicing or polyadenylation leads to altered exon structure and EJC binding in different tissue types. Indeed, we found that 100 of the tissue-specific m^6A peaks that escape EJC suppression span exon-intron boundaries and have significant m^6A-IP read coverage in regions annotated as intronic in the canonical transcript. Accordingly, our analyses suggest that the regions which are ordinarily suppressed by the EJC because of their close proximity to flanking upstream and downstream EJCs, appear to physiologically escape m^6A suppression due to the lack of splicing and EJC deposition on the unspliced exon-intron boundary, as depicted for *SGSM2*, *FAM120A* and *MBTPS1* as examples (fig. S22D and table S3).

We inspected the aligned m^6A-IP reads and observed that these m^6A peaks contain reads that span the canonical exon-intron junction, as depicted for *SGSM2, FAM120A* and *MBTPS1* as

examples (fig. S22D and table S3). These junction-spanning reads represent RNA isoforms that do not use that splice site, and thus presumably lack EJC binding at that location. This suggests that methylation of alternative transcript isoforms mediates the differences in m^6A methylation across tissues. We quantitatively assessed across tissues the correlation between the number of m^6A-IP reads that span the canonical exon-intron junction to the m^6A enrichment at the corresponding peak region in each tissue. These junction-spanning reads represent RNA isoforms that do not use that splice site, and thus presumably lack EJC binding at that location. Indeed, the number of reads that span the exon-intron junction in each tissue correlates with variation in m^6A enrichment across tissues (fig. S22E). Thus, expansion of exon length and removal of a flanking EJC appear to allow the suppressed m^6A sites within the internal exon to escape EJC suppression and acquire methylation across different tissues.

We next probed the identities of these alternative transcript isoforms. We analyzed transcriptome-wide intron retention in tissues with IRFinder and found that 53 of these exon-intron spanning m^6A peaks reside on alternatively spliced, retained introns, including the *SGSM2* m^6A peak (fig. S22D and table S4) (*32*). Some of the peaks appear to occur on alternative isoforms that are generated through intronic alternative cleavage and polyadenylation (APA), illustrated by *FAM120A* and *MBTPS1* (fig. S22D). Both these alternative pre-mRNA processing events alter transcript exon structure by lengthening exons. This expansion of exon length and removal of a flanking EJC appears to allow the suppressed m^6A sites within the internal exon to escape EJC suppression and acquire methylation (fig. S22F). This model is consistent with the results from our *CRY1* minigene experiments, in which we demonstrated that removal of either an upstream or downstream intron is sufficient for m^6A in an average-length internal exon to escape suppression (Fig. 2A).

Our results suggest that a subset of EJC-suppressed regions can physiologically escape suppression through methylation of transcripts with longer exons and generate tissue-specific m^6A patterns that affect tissue-specific gene expression (fig. S22, D to F and table S4). A previous study identified m^6A peaks in LCLs that vary in methylation levels due to interindividual genetic variation (*13*). We found that 48 of these variable peaks occur at EJC-suppressed regions in HeLa cells. Notably, variations in the m^6A levels of a subset of these peaks are significantly associated with complex traits, suggesting their functional importance (fig. S22G and table S5). For instance, genetic-mediated variation in methylation levels of an m^6A peak on *OGDH* is associated with variation in platelet counts. A SNP (rs740040) within an intron upstream of the *OGDH* m^6A peak is significantly associated with methylation level variation. Methylation variation in this transcript region appears to be driven by methylation of an APA transcript isoform, pointing to the functional relevance of this mode of m^6A regulation (fig. S22H). Note that we cannot exclude the possibility that variation in m^6A levels is correlated with variation in another molecular process that is causal for the phenotypes. Also, additional EJC-suppressed m^6A peaks in both tissues and LCLs can escape suppression without apparent changes in transcript structure, suggesting the presence of additional pathways to enable methylation of these regions. Altogether, these results indicate that a subset of EJC-suppressed m^6A sites can escape suppression in certain physiological contexts, which contributes to methylation variation across tissues and individuals.

EJCs may mediate m^6A suppression and gene expression across organisms

In both human and mouse, we observed a strong correlation between EJC loading, represented by the number of exons, and mRNA level across tissues, and showed that tissue-specific m^{6}A deposition regulated by EJC affects gene expression (Fig. 4, fig. S17, and fig. S21, A to B). We further investigated this correlation in other organisms. This correlation can be extended to vertebrates such as zebrafish, but not fly and worm (fig. S23). Comparing the exon/intron length and m^{6}A metagene plot across these species indicates that the involvement of exon-intron architecture and EJCs for m^{6}A suppression may only be true for vertebrates (fig. S23). Vertebrates have three YTHDF proteins, YTHDF1, 2, and 3, with YTHDF2 mediating decay of methylated transcripts. Worm does not have orthologs of the YTH family proteins (*70*), and fly has only one YTHDF gene which seems to be an YTHDF1 homologue that does not mediate mRNA degradation but instead affect translation (*71*). It will be very interesting to further investigate whether the effect of exon-intron architecture on mRNA stability might co-evolve with YTHDF2 in vertebrates.

EJC-suppressed m^{6}A sites generally colocalize with EJC-suppressed splice sites

Transcriptome-wide, EJC-suppressed splice sites significantly colocalize with EJC-suppressed m^{6}A sites, representing common regions in which both the m^{6}A methylation and splicing machineries are suppressed by the EJC (Fig. 5D). Overall, we identify 592 EIF4A3-suppressed and 125 RNPS1-suppressed splice sites that reside within 100 nt of hypermethylated m^{6}A regions with *siEIF4A3* KD, with representative examples shown at *SENP3*, *HNRNPH1*, *DHX15*, *KPNA1* and *MARS* (Fig. 5E and fig. S26D and table S2). Consistent with the co-localization of suppressed methylation and splice sites, suppressed splice sites are predominantly found in average-length internal exons (fig. S26, E and F). Not all regions with relatively strong hypermethylation necessarily contain EJC-suppressed splice sites, which may be due to the differential availability and strength of DRACH motifs and splice sites in specific regions. Altogether, these results suggest that RNPS1-associated EJCs suppress both local cellular m^{6}A methylation and splicing through packaging of proximal RNA and point to exon architecture as an important determinant of local RNA accessibility to regulatory machineries.

A subset of RBPs preferentially bind long internal exons

If long internal exons are more broadly accessible than average-length internal exons, one would predict that other mRNA regulators beyond the m^{6}A methyltransferase complex may also prefer to bind long internal exons. We performed a systematic analysis of whether other RBPs show a binding preference for long internal exons (fig. S28A), using existing eCLIP data (*13*). For instance, *SPEN* mRNA contains an 8,176 nt internal exon that is highly methylated and is also bound by RBPs that prefer binding on long internal exons (fig. S28B). To ensure that this length preference does not simply reflect the fact that RBP binding sites are more likely to fall in long internal exons by chance due to their greater lengths relative to short internal exons, we also calculated the ratio of RBP binding to longer (> 200nt) versus shorter (< 200nt) internal exons and normalized by the number of nucleotides in each group. We still observed a strong preference for long internal exons for this subset of RBPs (fig. S28C).

Note that our model does not predict that all RBPs would prefer to bind long exons. RBPs that are added on before EJC deposition would not be expected to prefer long exons. Indeed, we

found that RBPs like EFTUD2, SF3B4, which are spliceosome components, prefer to bind short internal exons (fig. S28C). This observation makes sense given that these RBPs should obligately bind at all exons regardless of length. Once normalized for total nucleotide length of exons, they exhibit preferential binding at shorter internal exons. Other splicing factors such as SRSF1 and ZRANB2 also show this bias. Conversely, as expected, RBM15, an m^6A methyltransferase complex subunit, prefers long exons (fig. S28C). Furthermore, any accessibility regulation by the EJCs would be a layer of regulation on top of intrinsic sequence preferences, which may bias certain RBPs towards shorter or longer internal exons at baseline even if they are impacted by the EJC-mediated mRNA packaging.

These observations suggest that EJCs could possibly regulate mRNA accessibility to a broader range of mRNA regulators in addition to the splicing and m^6A methylation machineries through its mRNA packaging function.

Active demethylation unlikely contributes to the EJC-mediated m^6A suppression

As shown in Fig. S6B, FTO and ALKBH5 binding sites are not enriched near suppressed m^6A sites, making it unlikely that they suppress a large portion of these sites through demethylation. Additionally, we have never seen notable m^6A landscape changes in mRNA m^6A-MeRIP-seq with FTO or ALKBH5 KD or KO, indicating that if demethylation contributes it would be very minimal as compared with EJC KD (*72–76*). The vast majority of hypermethylation upon FTO or ALKBH5 KD is increased m^6A level at sites that are already methylated at some level in the control samples, not sites that are completely suppressed as is seen in EIF4A3 KD (*72–76*). Consistent with the changes observed in sequencing data, FTO or ALKBH5 KD only leads to a ~20% increase in global mRNA m^6A level measured by UHPLC-QQQ-MS/MS (*72–76*), while EIF4A3 KD can cause up to a 2-fold increase in global mRNA m^6A level (fig. S8C).

Alternative polyadenylation (APA) may contribute to m^6A suppression in 3’UTR regions

We identify thousands of suppressed m^6A sites in 3’UTRs, downstream of stop codons (Fig. 1C). The EJC does not appear to mediate suppression of these sites. Transcript isoforms that use proximal 3’UTR polyadenylation sites tend to be methylated, while those that use distal 3’UTR polyadenylation sites tend to be unmethylated (*36*, *77*). Many of the identified suppressed 3’UTR sites reside in 3’UTR regions only present on the transcript isoforms generated from distal polyadenylation sites (fig S29) (*36*, *77*). Thus, additional, currently uncharacterized m^6A suppressing pathways most likely exist, which prevent m^6A deposition on transcript isoforms generated using distal polyadenylation sites and contribute to global depletion of m^6A downstream of stop codons. We note that other yet-to-be characterized m^6A suppressors could also potentially contribute to suppression of m^6A in the CDS, as EIF4A3 KD does not appear to completely restore methylation to unspliced levels (fig. S8H).

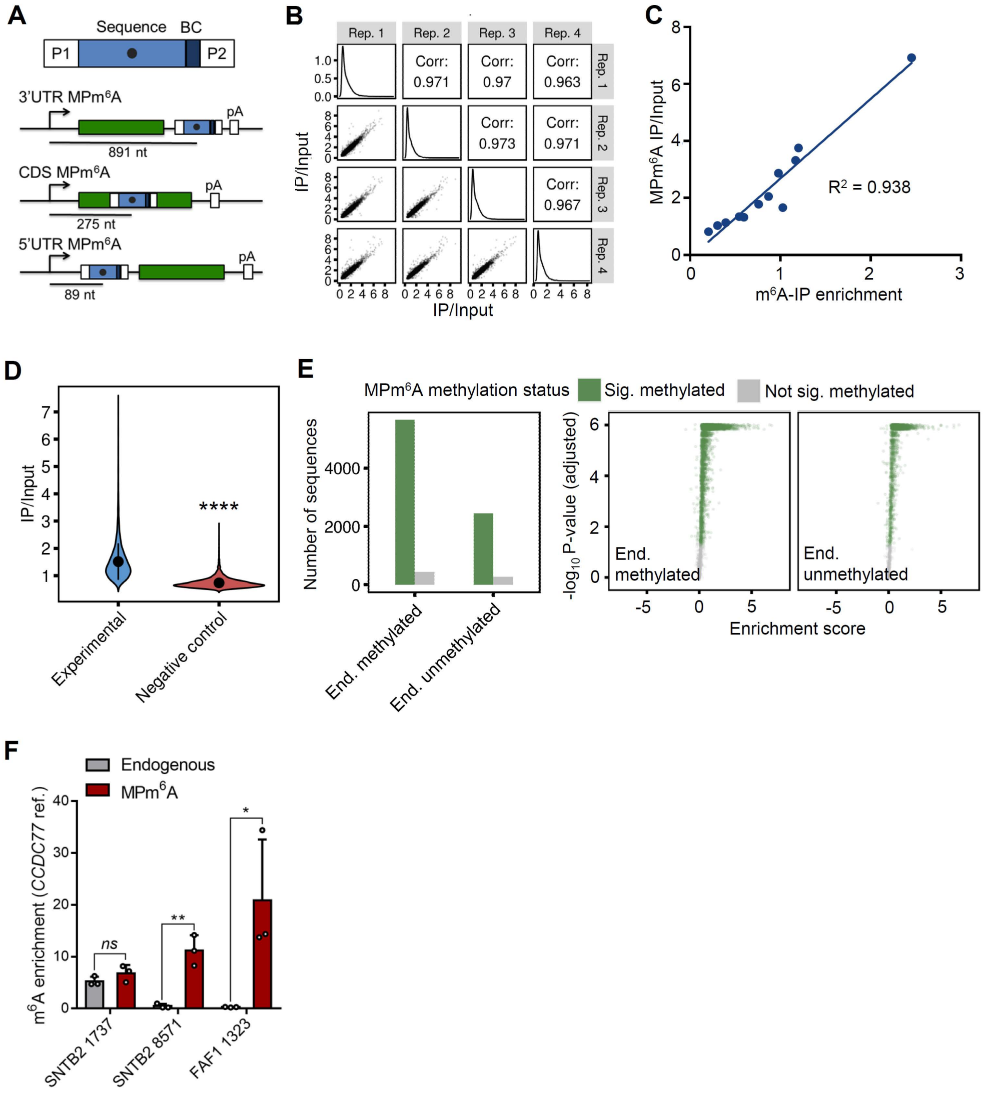

Fig. S1. MPm^{6}A reveals widespread suppression of transcriptomic m^{6}A sites. (A) Schematic model of MPm^{6}A reporter constructs with key elements illustrated including 5'UTR, CDS, 3'UTR, and pA denoting the polyA site. P1 and P2 are universal priming sequences. BC is a unique barcode. Sequence with m^{6}A motif is shown as a grey dot within the center. Distance between promoter and m^{6}A site are labeled for each set of constructs. **(B)** Scatterplots of 3'UTR MPm^{6}A (sequences cloned into 3'UTR of reporter plasmid) IP/Input for four biological replicates (bottom left). Histograms of IP/Input for individual replicates (middle). Pearson correlation for biological replicates (top right). **(C)** Scatterplot of MPm^{6}A (3'UTR) IP/Input and m^{6}A-IP-qPCR normalized enrichment values for 12 MPm^{6}A sequences. We observed high correlations in m^{6}A enrichment ratios between four biological replicates and a strong correlation in m^{6}A level measurements between MPm^{6}A and conventional m^{6}A-IP qPCR. **(D)** MPm^{6}A (3'UTR) IP/Input for experimental and negative control mutant sequences, mean ± SD, Wilcoxon rank-sum test, ****$P < 2.2e\text{-}16$. As expected, negative control sequences were depleted following m^{6}A-IP. **(E)** Numbers of endogenously methylated and endogenously unmethylated sites that are significantly methylated ($P < 0.05$, Wilcoxon rank sum test with BH procedure to control FDR) or not significantly methylated ($P > 0.05$) in MPm^{6}A (3'UTR), four biological replicates (left). MPm^{6}A (3'UTR)

enrichment score and p-values for endogenously methylated and endogenously unmethylated sequences. Significance threshold defined at P<.05, Wilcoxon rank-sum test with BH procedure to control FDR (right). **(F)** m^6A methylation at *SNTB2* 1737, *SNTB2* 8571, and *FAF1 1323* sequences in endogenous mRNA or in RNA expressed from the MPm^6A reporter (cloned into 3'UTR), assessed by m^6A-IP-qPCR, mean ± SEM, two-tailed T-test, $*P < 0.05$; $**P < 0.01$, m^6A enrichment is normalized to *CCDC77* mRNA, three biological replicates. Numbers following the gene names indicate the position of the m^6A site or unmethylated DRACH site relative to the transcription start site.

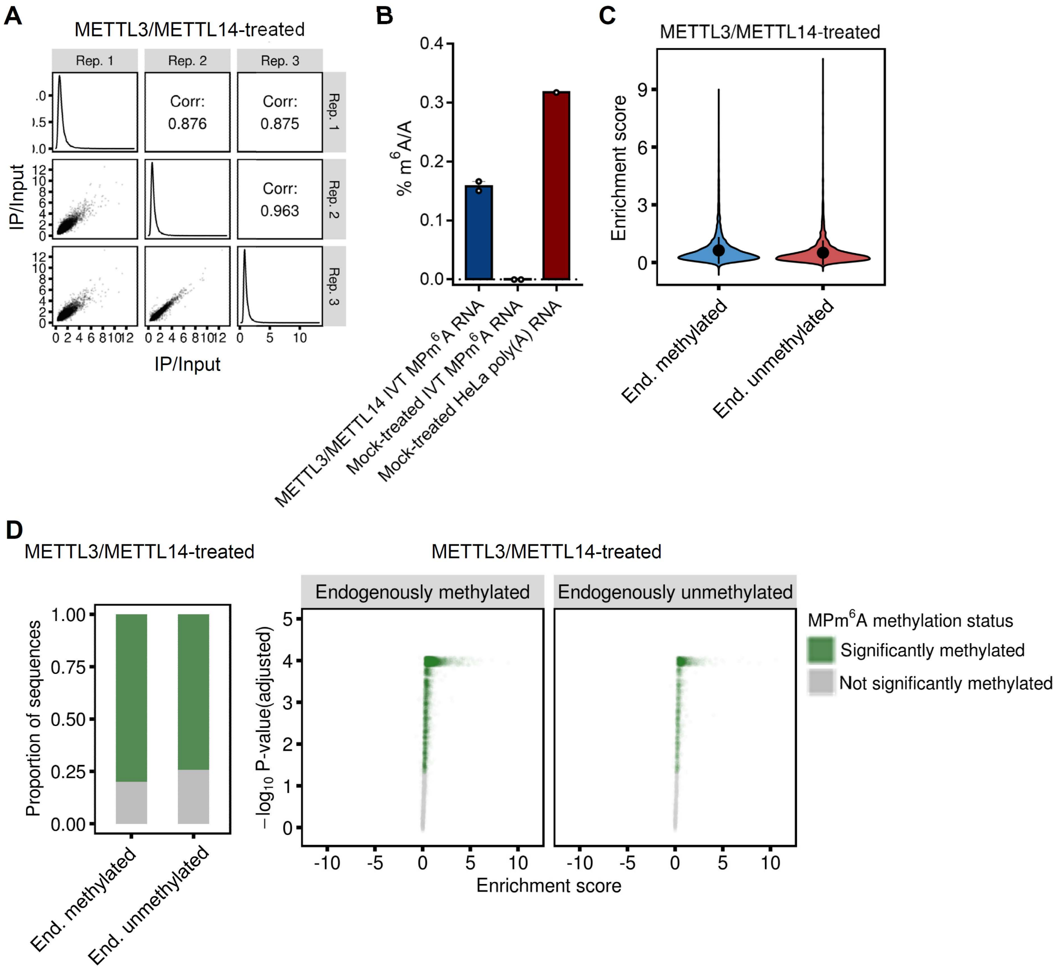

Fig. S2. ***In vitro*** **MPm^{6}A reveals that local sequence context is not sufficient to specify endogenous methylation status**. **(A)** Scatterplots of *in vitro* methylated MPm^{6}A (3'UTR) IP/Input for three replicates (bottom left) using recombinant METTL3/METTL14 treatment. Histograms of IP/Input for individual replicates (middle). Pearson correlation between replicates (top right). **(B)** LC-MS/MS measurement of m^{6}A levels of *in vitro* transcribed MPm^{6}A mRNA methylated by the recombinant METTL3/METTL14 treatment *in vitro* methylated, mock-treated (all *in vitro* methylation reaction components, but omitting recombinant METTL3/METTL14) *in vitro* transcribed MPm^{6}A mRNA, and mock-treated cellular polyadenylated RNA. **(C)** Enrichment scores for endogenously methylated and endogenously unmethylated sequences for recombinant METTL3/METTL14-treated *in vitro* MPm^{6}A (3'UTR), mean ±SD. **(D)** Proportion of sequences that are significantly methylated ($P < 0.05$) or not significantly methylated ($P > 0.05$) in recombinant METTL3/METTL14-treated *in vitro* MPm^{6}A (3'UTR). Wilcoxon rank sum test with BH procedure to control FDR, three replicates (left). Enrichment scores and p-values for endogenously methylated and unmethylated sequences in MPm^{6}A (3'UTR) with recombinant METTL3/METTL14 treatment *in vitro*. Significance threshold defined at $P < 0.05$, Wilcoxon rank-sum test with BH procedure to control FDR, three replicates (right).

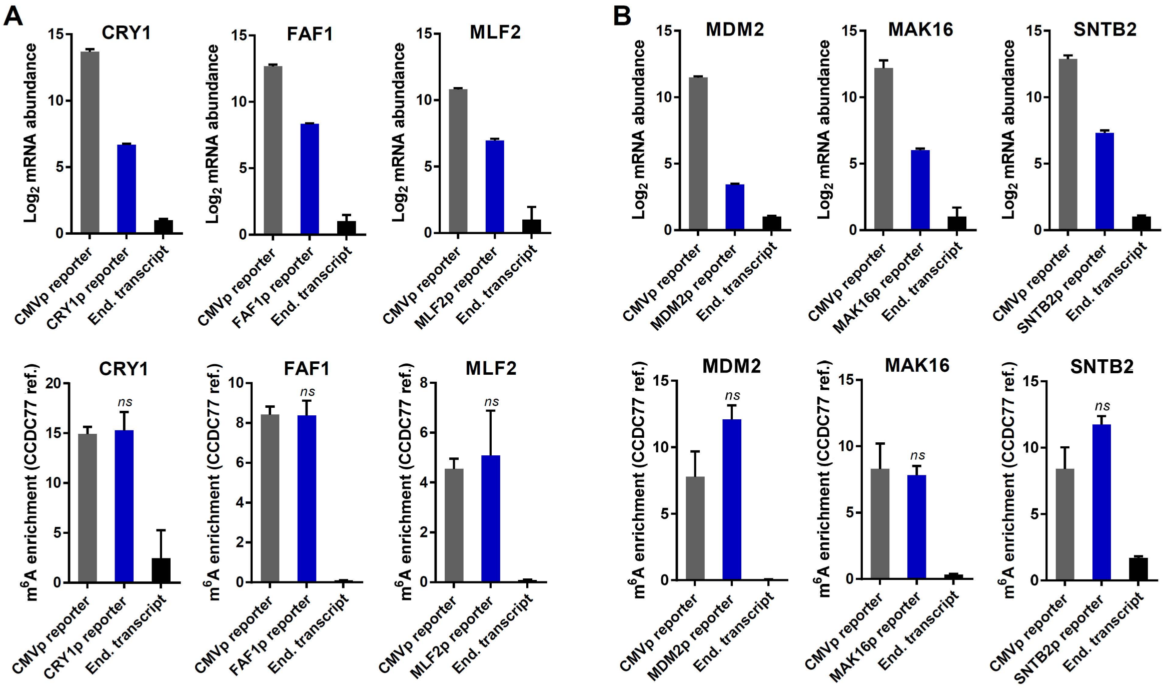

Fig. S3. Consistent patterns were obtained from MPm^{6}A with endogenous promoters and a CMV promoter. (**A**) For selected CDS suppressed sites, the differences in mRNA abundance (top) and m^{6}A enrichment (bottom) between reporters with a CMV promoter and an endogenous promoter. (**B**) For selected 3'UTR suppressed sites, the differences in mRNA abundance (top) and m^{6}A enrichment (bottom) between reporters with a CMV promoter and an endogenous promoter. Three biological replicates.

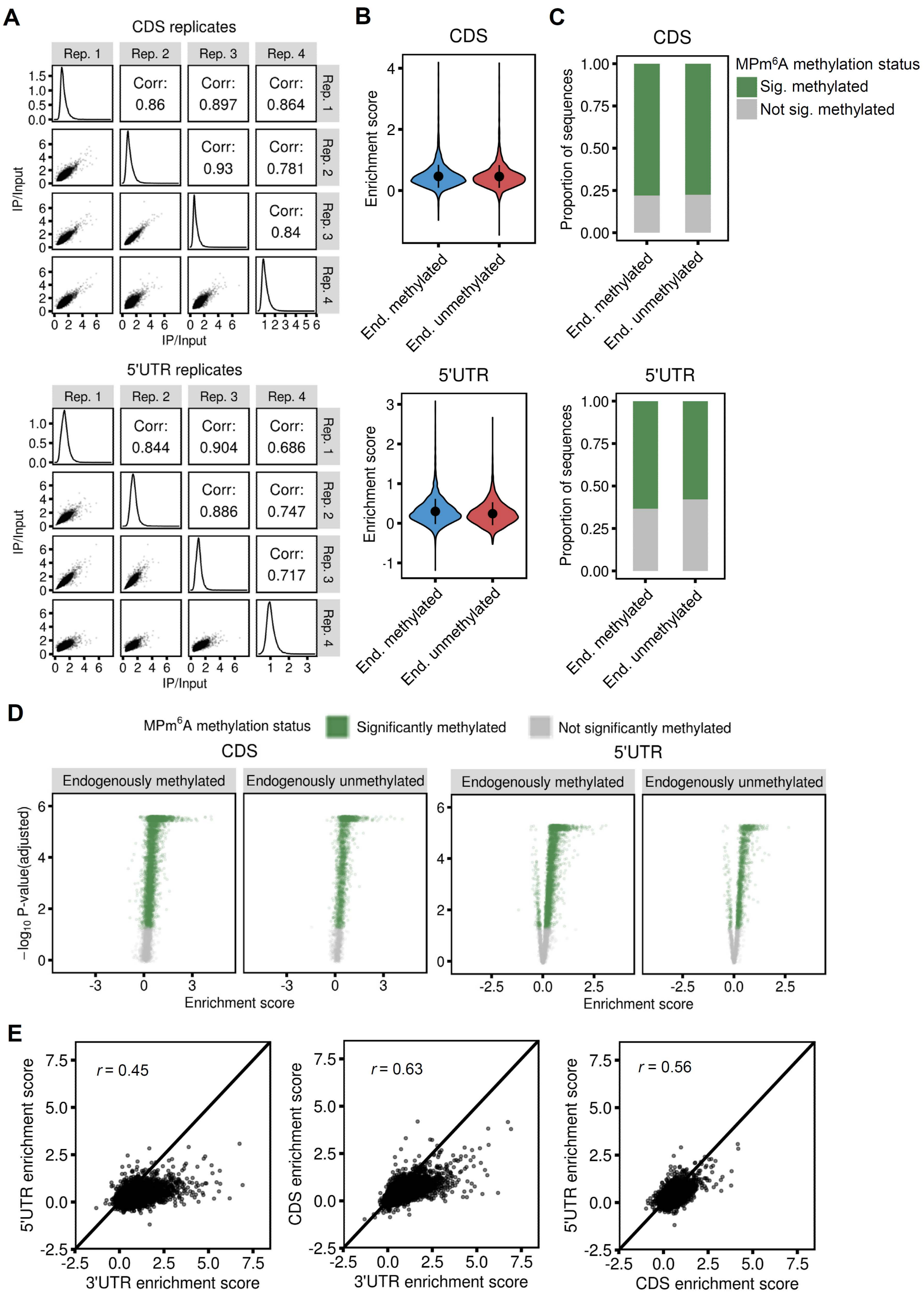

Fig. S4. CDS MPm^{6}A and 5’UTR MPm^{6}A reveal that local sequence context is not sufficient to specify endogenous methylation status. **(A)** Scatterplots of CDS MPm^{6}A IP/Input (left) and 5’UTR MPm^{6}A IP/Input (right) for four biological replicates, (bottom left). Histograms of IP/Input for individual replicates (middle). Pearson correlation for biological replicates (top right). **(B)** CDS MPm^{6}A enrichment scores (top) and 5’UTR MPm^{6}A enrichment scores (bottom) for

endogenously methylated and endogenously unmethylated sequences, mean ±SD. **(C)** Fractions of endogenously methylated and endogenously unmethylated sequences that are significantly methylated ($P < 0.05$, Wilcoxon rank sum test with BH procedure to control FDR) or not significantly methylated ($P > 0.05$) for CDS MPm^{6}A (top) and 5'UTR MPm^{6}A (bottom), four biological replicates. **(D)** CDS MPm^{6}A (top) and 5'UTR MPm^{6}A (bottom) enrichment scores and p-values for endogenously methylated and endogenously unmethylated sequences. Significance threshold defined at $P < 0.05$, Wilcoxon rank-sum test with BH procedure to control FDR. **(E)** Scatterplots of 5'UTR and 3'UTR MPm^{6}A enrichment scores (left), CDS and 3'UTR MPm^{6}A enrichment scores (middle), 5'UTR and CDS MPm^{6}A enrichment scores (right), for endogenously methylated sequences. A y = x line is plotted for reference. 5'-end regions are less permissive to m^{6}A deposition than 3'-end regions.

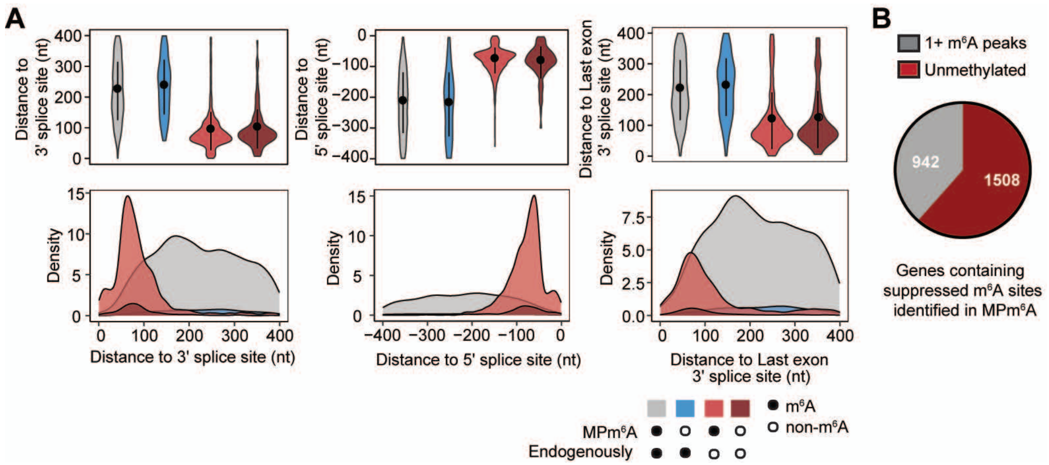

Fig. S5. Distribution of MPm^{6}A sites across the transcriptome. (A) The relative density of endogenously (un)methylated sites to 400 nt downstream and upstream 3' (*left* two panels) and 5' (*middle* two panels) splice sites, and 400 nt downstream last exon 3' splice sites (*right* two panels). The y-axis in the density plot shows the proportional quantity of sites for each group. Sample size for each group (color in the legend) from left to right: 5657, 438, 2450, and 266. (**B**) Number of genes containing suppressed m^{6}A sites identified in MPm^{6}A that exhibit one or more m^{6}A peaks on other regions of the endogenous transcript, and number that does not exhibit m^{6}A on any part of the transcript. Endogenously methylated mRNAs were defined as mRNAs with at least one m^{6}A peaks in control HeLa cells from the m^{6}A-seq data used when designing MPm^{6}A.

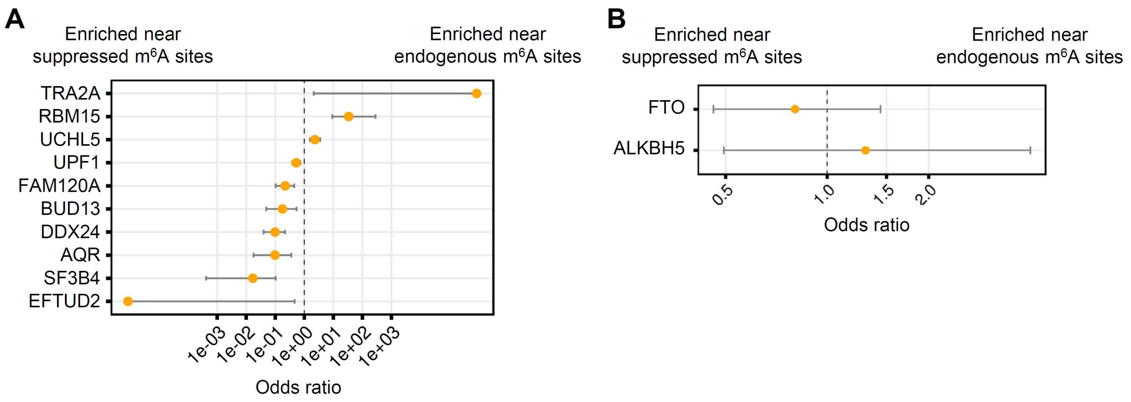

Fig. S6. Spliceosome components are enriched near suppressed m^{6}A sites. (A) RBPs with statistically significant enrichment or depletion (P < 0.05, Fisher's exact test with BH procedure to control FDR) of binding sites at endogenously methylated or endogenously unmethylated MPm^{6}A sequences that are significantly methylated in MPm^{6}A. Dot and bar represent odds ratio and 95% confidence interval. **(B)** Enrichment of FTO and ALKBH5 binding sites at endogenously methylated or endogenously unmethylated MPm^{6}A sequences that are significantly methylated in MPm^{6}A. Dot and bar represent odds ratio and 95% confidence interval. Analyzed from public eCLIP-seq (*13*).

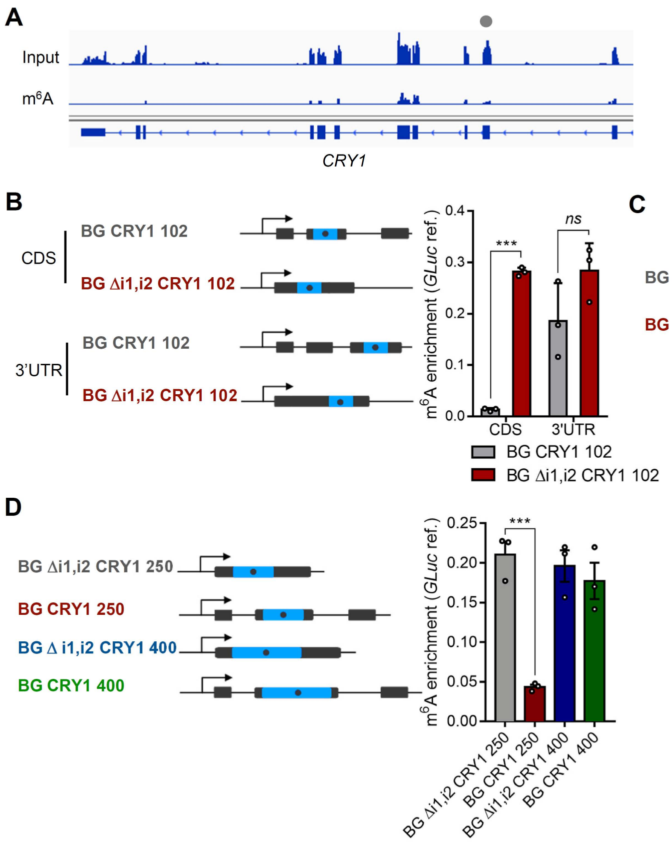

Fig. S7. Pre-mRNA splicing suppresses m^{6}A methylation in an exon length-dependent manner. **(A)** Input and m^{6}A-IP read coverage at the *CRY1* gene in HeLa cells, gray dot marks location of the suppressed m^{6}A site cloned into BG *CRY1* constructs. **(B)** m^{6}A methylation of specified BG *CRY1* constructs, with the CRY1 suppressed m^{6}A site cloned into CDS or 3'UTR of BG construct containing introns or with introns removed. Schematic of constructs (left), gray dot represents position of the *CRY1* suppressed m^{6}A site. Blue region indicates sequence derived from *CRY1*, gray region indicates sequence derived from rabbit beta-globin. Number following CRY1 refers to nucleotides surrounding the *CRY1* suppressed m^{6}A site in the endogenous *CRY1* mRNA that is cloned into BG constructs. m^{6}A enrichment at the *CRY1* suppressed m^{6}A site within mRNA expressed from the constructs (right). **(C)** m^{6}A methylation of specified BG *CRY1* constructs, with the 102 nt surrounding the *CRY1* suppressed m^{6}A site cloned into the internal exon of BG CRY1, or with 102 nt surrounding the *CRY1* suppressed m^{6}A site cloned into the internal exon of BG Δss CRY1, in which all the splice sites are deleted. Schematic of constructs (left), m^{6}A enrichment at the *CRY1* suppressed m^{6}A site (right). **(D)** m^{6}A methylation of specified BG *CRY1* constructs with the presence of introns and length of internal exon varied. Schematic of constructs (left), m^{6}A enrichment at the *CRY1* suppressed m^{6}A site (right). For (B) to (D), RNA is sheared to ~150 nt fragments and primers amplify 62 nt fragment containing the *CRY1* suppressed m^{6}A site. In each construct, grey dot in the blue region denotes the suppressed m^{6}A site; the number at the left and right of the m^{6}A site shows the distance (nt) between the m^{6}A site to 5' and 3' splice site, respectively; the number next to the TSS shows the distance (nt) between the m^{6}A site to the promoter. Mean ± SEM, two-tailed T-test, $**P < 0.01$, $***P < 0.001$, three biological replicates used in all panels.

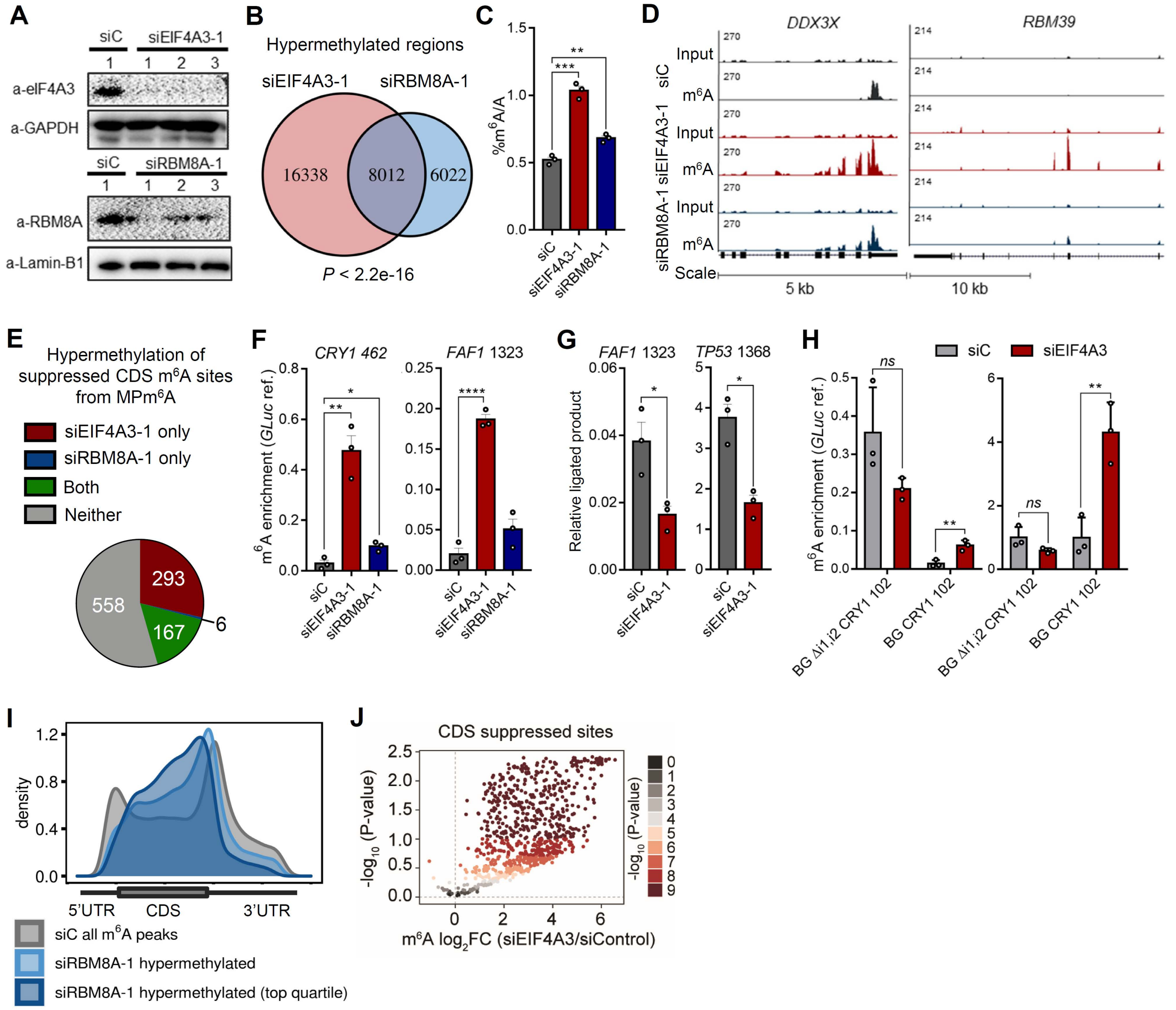

Fig. S8. The EJCs suppress exon junction-proximal m^{6}A methylation within coding sequences in thousands of mRNA transcripts. (A) Knockdown efficiency of *siEIF4A3*-1 and si*RBM8A*-1 in HeLa cells. **(B)** Overlap of hypermethylated regions upon *EIF4A3* and *RBM8A* KD in HeLa cells. *P*-value was calculated using Fisher's exact test. **(C)** LC-MS/MS measurement of m^{6}A levels of polyadenylated RNA from *EIF4A3* and *RBM8A* KD HeLa cells, mean ± SEM, two-tailed T-test, ****P* = 0.0002, ***P* = 0.0027. Three biological replicates **(D)** Input and m^{6}A-IP read coverage at *DDX3X* and *RBM39* upon non-targeting control KD, *EIF4A3* KD and *RBM8A* KD in HeLa cells. **(E)** Overlap of suppressed CDS m^{6}A sites identified in MPm^{6}A with hypermethylated regions upon *EIF4A3* and/or *RBM8A* KD. **(F)** m^{6}A methylation at *CRY1* 462 and *FAF1* 1323 suppressed m^{6}A sites upon *EIF4A3* and *RBM8A* KD in HeLa cells assessed by m^{6}A-IP-qPCR. 462 and 1323 denote the position of the site relative to the transcription start site, mean ± SEM, two-tailed T-test, ****P* < 0.0001, ***P* = 0.0019, **P* = 0.0163. Three biological replicates **(G)** m^{6}A methylation at *FAF1* 1323 and *TP53* 1368 suppressed m^{6}A sites upon *EIF4A3* KD in HeLa cells assessed by SELECT, mean ± SEM, two-tailed T-test, **P* < 0.05. Relative ligated product levels are inversely proportional to m^{6}A levels; a decrease in relative ligated product indicates an increase in m^{6}A. Three biological replicates **(H)** m^{6}A methylation enrichment at the *CRY1* suppressed m^{6}A

site in specified BG *CRY1* constructs upon *EIF4A3* KD. Results depicted as normalized m^6A enrichment (left) and fold-change compared to control KD (right). Number following *CRY1* refers to nucleotides surrounding the *CRY1* suppressed m^6A site in the endogenous *CRY1* mRNA that is cloned into the BG construct. Δi1,i2 denotes deletion of both introns. RNA is sheared to ~150 nt fragments and primers amplify 62 nt fragment containing *CRY1* suppressed m^6A site. m^6A level is normalized to m^6A^+ *in vitro* transcribed Gaussia luciferase RNA spike in, mean ±SEM, two-tailed T-test, $^{**}P < 0.01$, three biological replicates**.** **(I)** Metagenes of m^6A hypermethylated regions upon *RBM8A* KD in HeLa cells. **(J)** Volcano plot of suppressed sites in CDS with differential m^6A modification level in *EIF4A3* KD HeLa cells *versus* control cells. *P*-values were calculated using a two-tailed student's t-test.

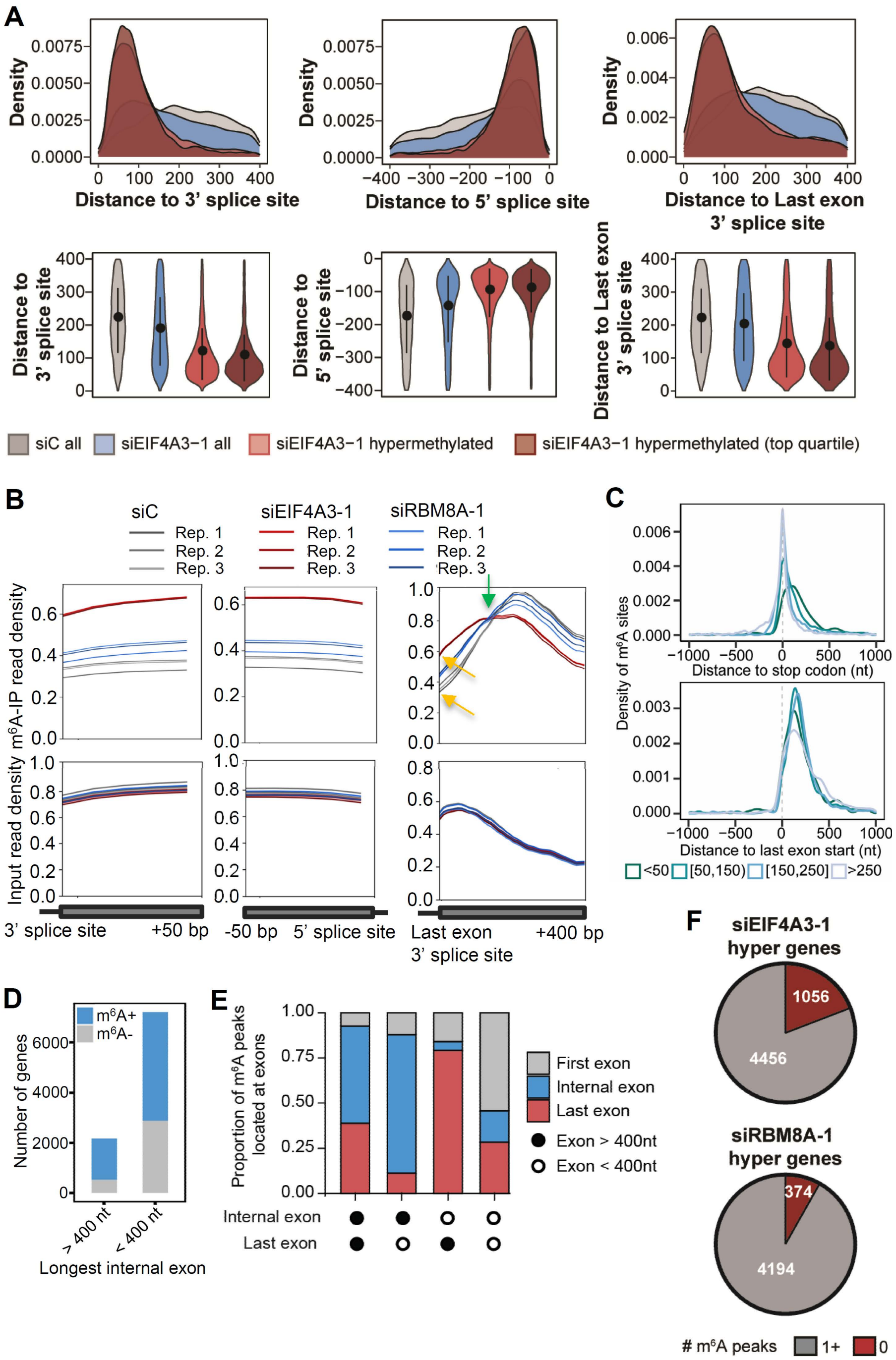

Fig. S9. Relationship between EJC-suppressed methylation and exon structure. (A) Density of m^{6}A sites to 400 nt downstream and upstream 3' (left) and 5' (middle) splice sites, respectively, and 400 nt downstream last exon 3' splice sites (right). m^{6}A in siC (grey) and *siEIF4A3* (blue) samples, as well as *siEIF4A3*-hypermethylated (red; top quartile, dark red), are shown in indicated colors, respectively. **(B)** Density of m^{6}A-IP reads (top) and input reads (bottom) 50 nt upstream and downstream 5' and 3' splice sites, respectively, and 400 nt downstream last exon 3' splice sites upon *EIF4A3* KD and *RBM8A* KD. m^{6}A density is ~0.6 for siEIF4A3 and ~0.4 for siC at the immediate start of the last exon (orange arrows). The m^{6}A density in the *siEIF4A3* samples remains elevated over the siC m^{6}A density until 100-150 nt downstream the 5' ends of last exons (green

arrow). **(C)** Distribution of m^6A sites relative to stop codon (top) or start of last exon (bottom). mRNAs were grouped according to their stop codon locations to the last exon start. m^6A sites were obtained from our m^6A-SAC-seq in HeLa cells cells (*78*). **(D)** Transcript methylation status of genes in HeLa cells, grouped by presence of an internal exon > 400 nt. m^6A+ indicates that transcripts of these genes contain at least one m^6A peak, m^6A- indicates that transcripts of these genes do not contain an m^6A peak. **(E)** The proportion of m^6A peaks in HeLa located in genes' first, internal and last exons. Genes were categorized according to their longest internal/terminal exon length (> 400 nt). **(F)** Number of hypermethylated genes upon *EIF4A3* KD or *RBM8A* KD with at least one m^6A peak in the control KD, and number of hypermethylated genes with no m^6A peaks in the control KD. Hypermethylated genes are defined as genes whose transcripts contain a new m^6A peak upon *EIF4A3* or *RBM8A* KD that is not present in the control KD.

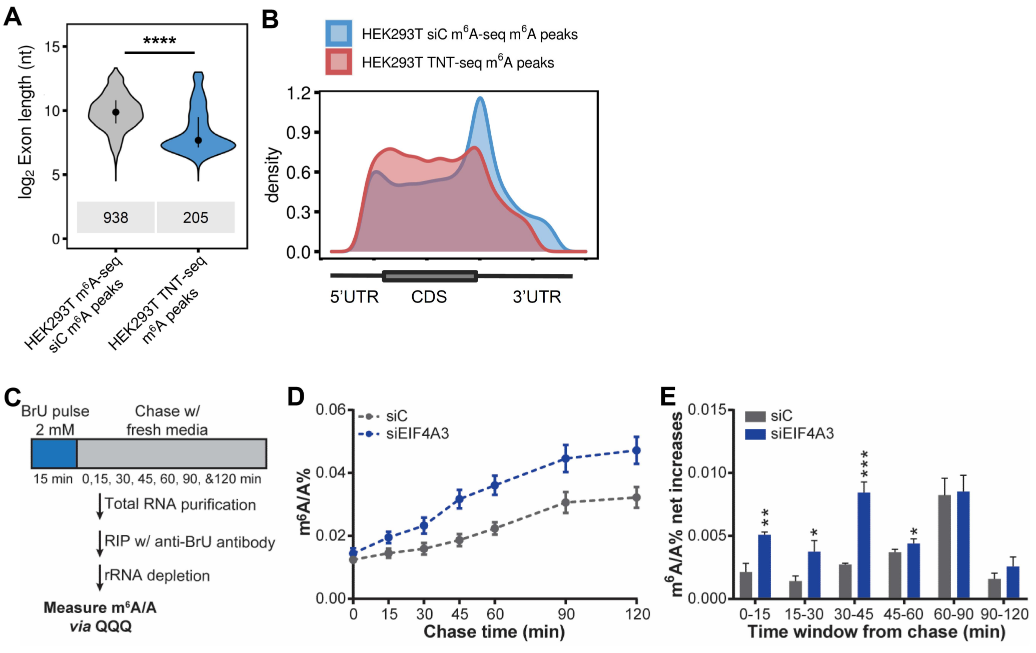

Fig. S10. Temporal analysis of m^{6}A deposition. (A) Exon lengths of m^{6}A peaks that reside in internal exons in m^{6}A-meRIP-seq of bulk polyadenylated RNA and Transient N-6-methyladenosine transcriptome sequencing (TNT-seq) of nascent RNA in HEK293T cells. Dot and bar represent median and interquartile range, Wilcoxon rank sum test, *****P* < 2.2e-16. **(B)** Metagenes of m^{6}A-seq and TNT-seq peaks in HEK293T cells. (**C**) Schematic model showing the design of the BrU pulse-chase experiments followed by UHPLC-QQQ-MS/MS measurements. (**D**) The m^{6}A/A ratio of nascent RNA after rRNA depletion in HeLa cells with or without *EIF4A3* KD at the indicated chase time. (**E**) The increase of the m^{6}A/A ratio within the indicated time window (time window is counted from the beginning of chase). *P*-values were determined using unpaired two-tailed *t*-tests; **P* < 0.05, ***P* < 0.01, ****P* < 0.001, Error bars and means ± SD are shown for *n* = 3 experiments.

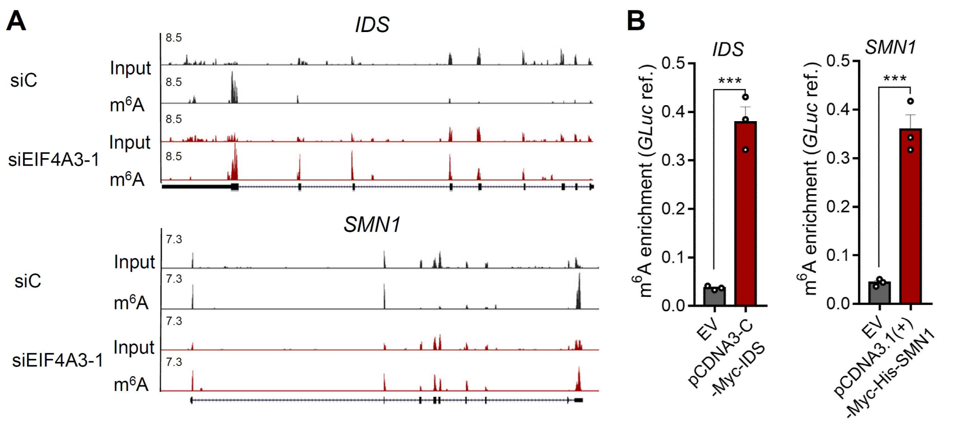

Fig. S11. *IDS* and *SMN1* mRNA expressed from unspliced cDNA expression constructs are hypermethylated compared to endogenous mRNA. (A) Input and m^6A-IP read coverage at *IDS* and *SMN1* upon non-targeting control KD and *EIF4A3* KD in HeLa cells. **(B)** m^6A enrichment on *IDS* and *SMN1* mRNA expressed from cDNA expression plasmids, or on endogenous mRNA (corresponding empty vector (EV) plasmid control transfection). m^6A enrichment is calculated by taking the IP/input ratio for the gene of interest, to control for expression level, and then normalizing to the IP/input ratio to an m^6A+ reference Gaussia luciferase spike in RNA, to control for IP efficiency. mean ± SEM, two-tailed T-test, ***$P < 0.0001$, three biological replicates.

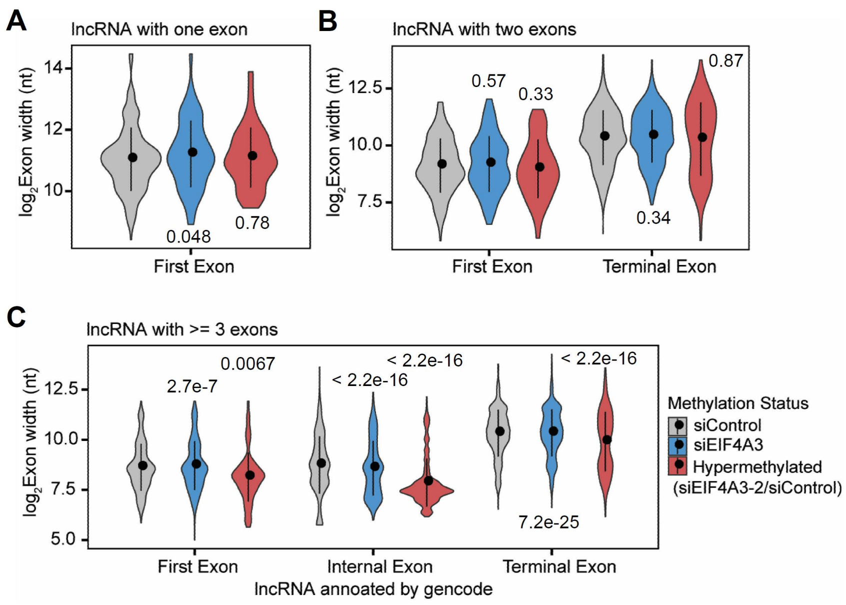

Fig. S12. EJCs suppress exon-junction-proximal m^{6}A methylation within coding sequences in lncRNAs with more than three exons. (A-C) Exon length of all m^{6}A peaks residing within long non-coding RNAs (lncRNAs) with different types of exons in control (siC), *EIF4A3* KD (*siEIF4A3*), and exon lengths of m^{6}A hypermethylated regions within different types of exons comparing *EIF4A3* KD with the control HeLa cells. Wilcoxon rank sum test of indicated groups *vs.* siC exon length and *P* values are shown.

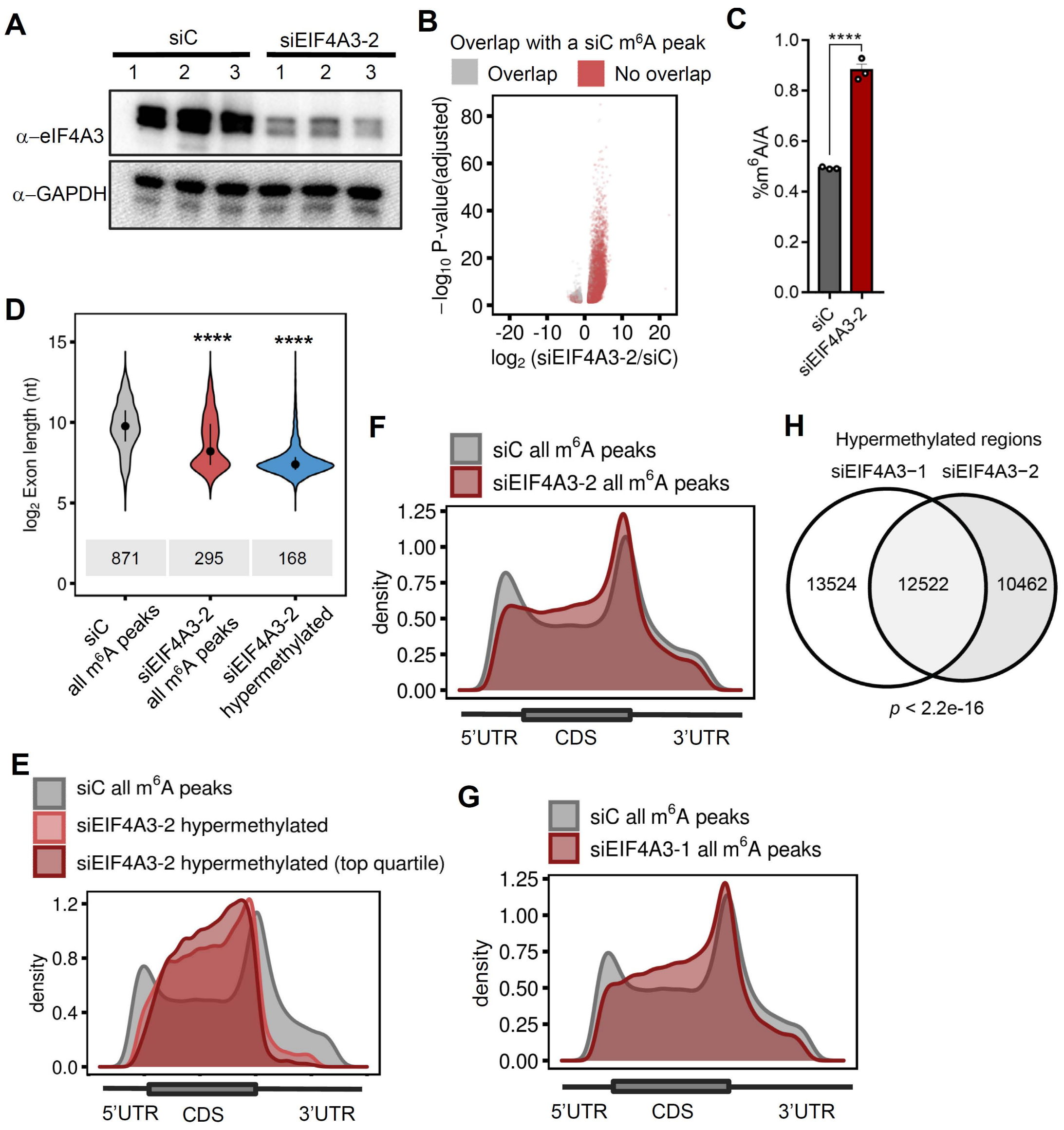

Fig. S13. Knockdown of *EIF4A3* with an alternative siRNA confirms EIF4A3-mediated suppression of exon junction-proximal m^6A methylation within coding sequences. (A) Knockdown efficiency of *siEIF4A3*-2 in HeLa cells. **(B)** Differentially methylated regions upon *siEIF4A3*-2 knockdown (KD) in HeLa cells (FDR<.1, $|log_2FC|>1$), three biological replicates. Gray dots indicate differentially methylated regions that overlap m^6A peaks in the non-targeting control knockdown cells, red dots indicate differentially methylated regions that do not overlap m^6A peaks in the control. **(C)** LC-MS/MS of m^6A of polyadenylated RNA from *siEIF4A3*-2 HeLa cells, mean ± SEM, two-tailed T-test, ****$P < 0.0001$. Three biological replicates **(D)** Exon lengths of all m^6A peaks within internal exons in control and upon KD with *siEIF4A3*-2, and exon lengths of m^6A hypermethylated regions within internal exons upon KD with *siEIF4A3*-2. Dot and bar represent median and interquartile range, Wilcoxon rank sum test, ****$P < 2.2e\text{-}16$. **(E)** Metagenes of m^6A hypermethylated regions upon KD with *siEIF4A3*-2 in HeLa cells. **(F)** Metagenes of all m^6A peaks in control and *siEIF4A3*-2 KD cells. **(G)** Metagene plots of all m^6A peaks upon control KD and all m^6A peaks upon *siEIF4A3*-1 KD in HeLa cells. **(H)** The overlap between the hypermethylated regions upon the *EIF4A3* knockdown with two different siRNAs in HeLa cells.

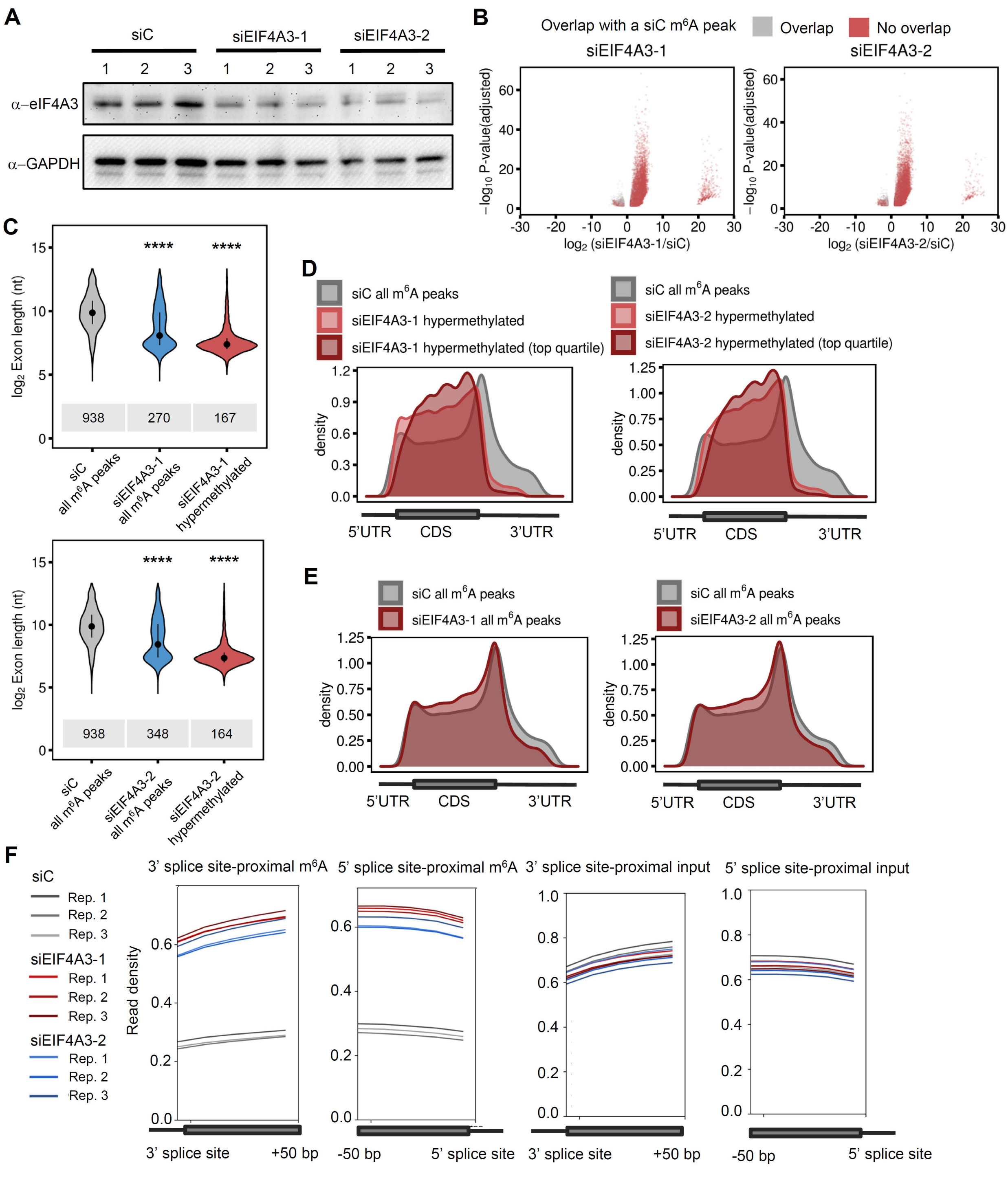

Fig. S14. EIF4A3 globally suppresses exon junction-proximal m⁶A methylation within mRNA coding sequences in HEK293T cells. (A) Differentially methylated regions upon *EIF4A3* KD in HEK293T (FDR<.1, $|\log_2 FC|>1$) with two different siRNAs, three biological replicates. Gray dots indicate differentially methylated regions that overlap m^{6}A peaks in the non-targeting control knockdown cells, red dots indicate differentially methylated regions that do not overlap m^{6}A peaks in the control. **(B)** Knockdown efficiency of *siEIF4A3*-1 and *siEIF4A3*-2 in HEK293T cells. **(C)** Exon lengths of all m^{6}A peaks within internal exons in control and upon *EIF4A3* KD,

and exon lengths of m^{6}A hypermethylated regions within internal exons upon *EIF4A3* KD in HEK293T. Dot and bar represent median and interquartile range, Wilcoxon rank sum test of specified group vs. siC all m^{6}A peaks, ****$P < 2.2e\text{-}16$. **(D)** Metagenes of all m^{6}A peaks in control and significantly m^{6}A hypermethylated regions upon *EIF4A3* KD in HEK293T cells. **(E)** Metagenes of all m^{6}A peaks in control and of all m^{6}A peaks upon *EIF4A3* KD in HEK293T cells. **(F)** Density of m^{6}A-IP and input reads near 5' and 3' splice sites upon *EIF4A3* KD in HEK293T cells.

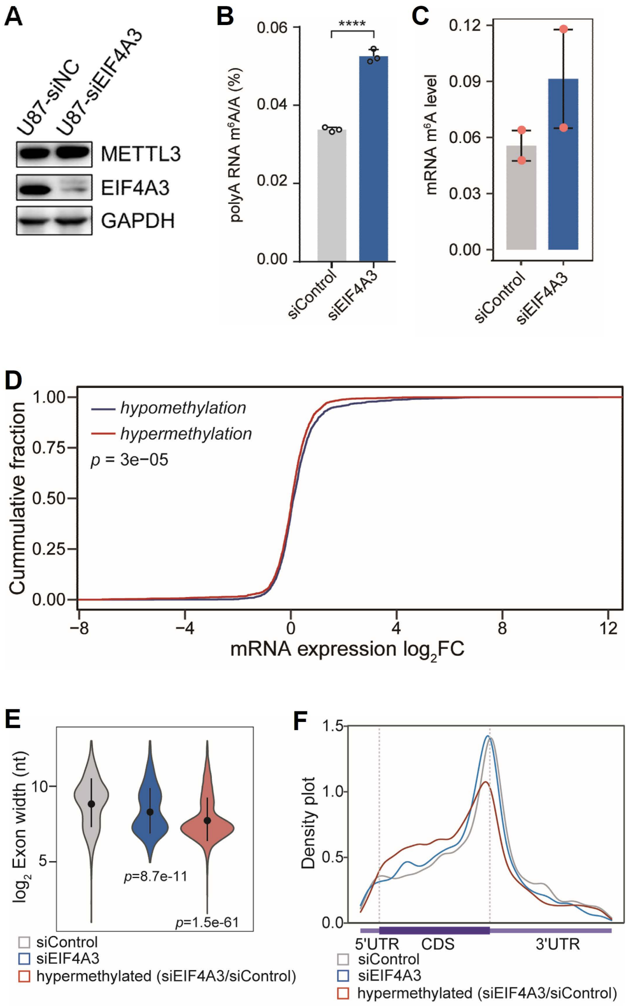

Fig. S15. EIF4A3 globally suppresses exon junction-proximal m^{6}A methylation within mRNA coding sequences in a glioblastoma cancer cell line. (**A**) Western blot of EIF4A3 and METTL3 levels in control and *EIF4A3* KD U87 cells. (**B**) LC-MS/MS quantification of the m^{6}A/A ratio in polyA from control or *EIF4A3* KD U87 cells. $n = 3$ biological replicates; error bars indicate means ± SEM. (**C**) m^{6}A levels were quantified through normalizing m^{6}A sequencing results with spike-in in WT and *EIF3A3* KD in U87 cells. $n = 2$ biological replicates. (**D**) Transcripts with m^{6}A hypermethylation showed reduced mRNA expression level compared to those with m^{6}A hypomethylation. (**E**) Exon length of all m^{6}A peaks residing within internal exons in control, *EIF4A3* KD, and exon lengths of m^{6}A hypermethylated regions upon *EIF4A3* KD in U87 cells. Dot and bar represent the median and interquartile range, Wilcoxon rank sum test of indicated groups *vs*. control all m^{6}A peaks. (**F**) Metagene plot of all m^{6}A peaks in control, *EIF4A3* KD, and hypermethylated m^{6}A peaks upon *EIF4A3* KD in U87 cells.

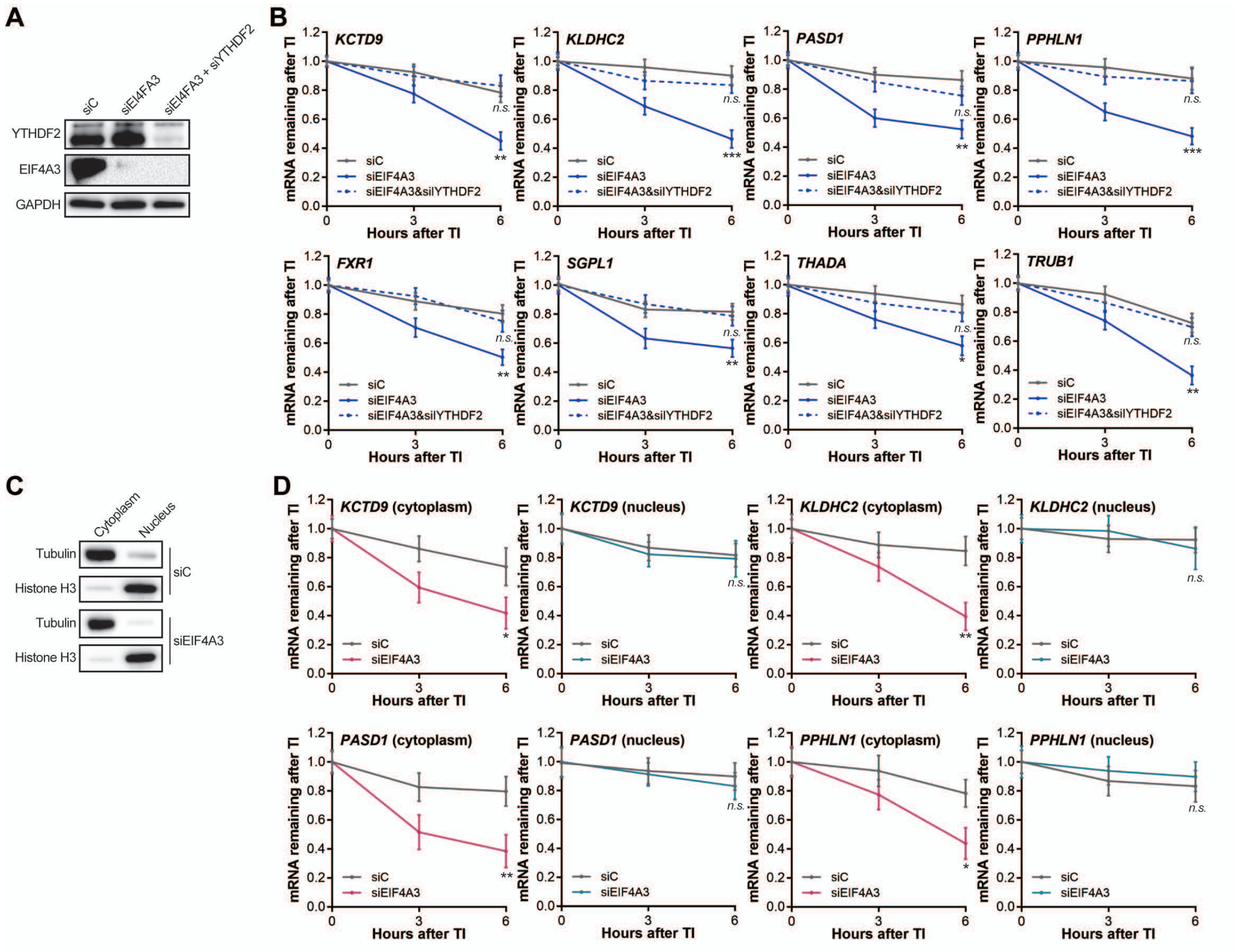

Fig. S16. ***YTHDF2*** **KD in** ***EIF4A3*** **KD HeLa cells rescued reduced mRNA half-life of selected hypermethylated transcripts.** (**A**) Western blots showing the KD efficiency of *EIF4A3* and *YTHDF2*. (**B**) mRNA stability of selected top YTHDF2 targets in control, *EIF4A3* KD, and *EIF4A3* and *YTHDF2* double KD (as rescue) in HeLa cells. TI denotes transcription inhibition. Transcription was inhibited by actinomycin D. (**E**) Western blots showing the fractionation efficiency of cell nucleus and cytoplasm. (**F**) mRNA stability of selected top YTHDF2 targets in the cytoplasm *versus* nucleus for control and *EIF4A3* KD in HeLa cells. *P*-values were determined using unpaired two-tailed *t*-tests; **P* < 0.05, ***P* < 0.01, ****P* < 0.001, Error bars and means ± SD are shown for indicated group *vs*. siC for *n* = 3.

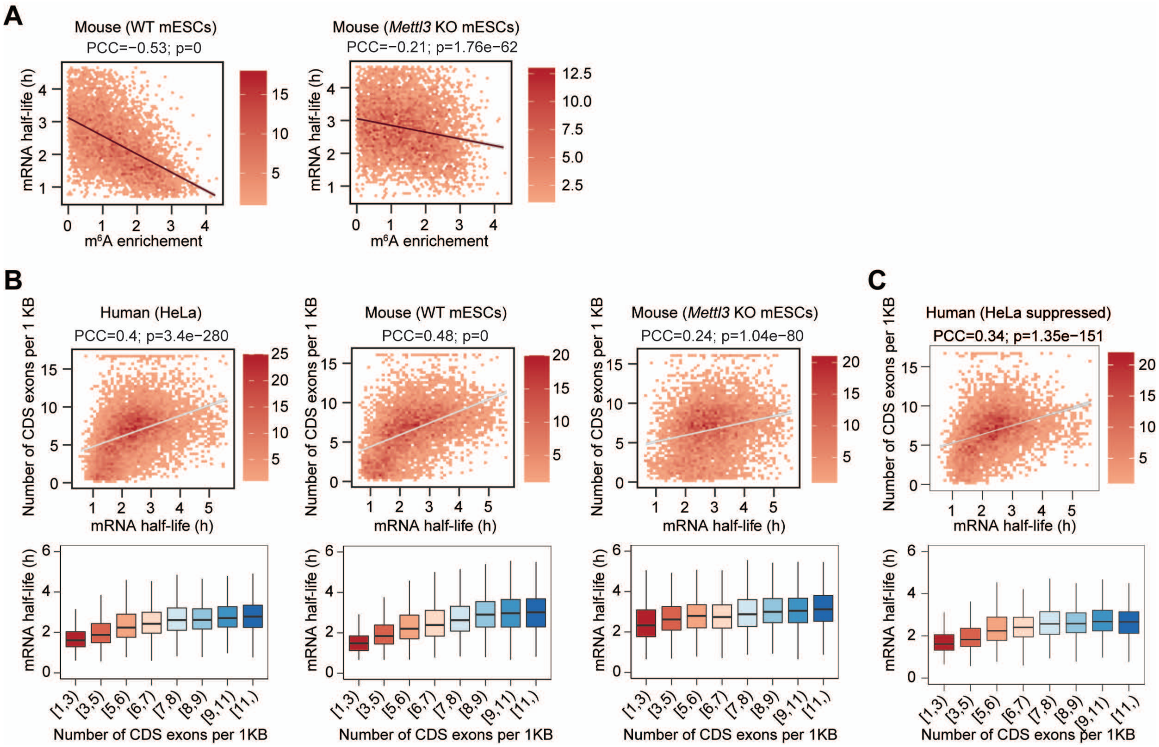

Fig. S17. EJC loading correlates with mRNA half-life in HeLa and mES cells. (**A**) The correlation between m^{6}A (GSE183399, unpublished) and mRNA half-life (GSE86336) in WT (left) and *Mettl* KO (right) mESCs. The Pearson Correlation Coefficient (PCC) and *P* values are shown. (**B**) The correlation between mRNA half-life and number of exons (representing EJC loading) within CDS regions per 1 KB in human (GSE49339) (left) and WT (middle) and *Mettl3* KO (right) mESCs (GSE86336), respectively. PCC and *P* values are shown. (**C**) The correlation between mRNA half-life and number of exons within CDS regions per 1 KB (representing EJC loading) in human (GSE49339) for transcripts with EJC-suppressed m^{6}A identified in HeLa cells. PCC and *P* values are shown.

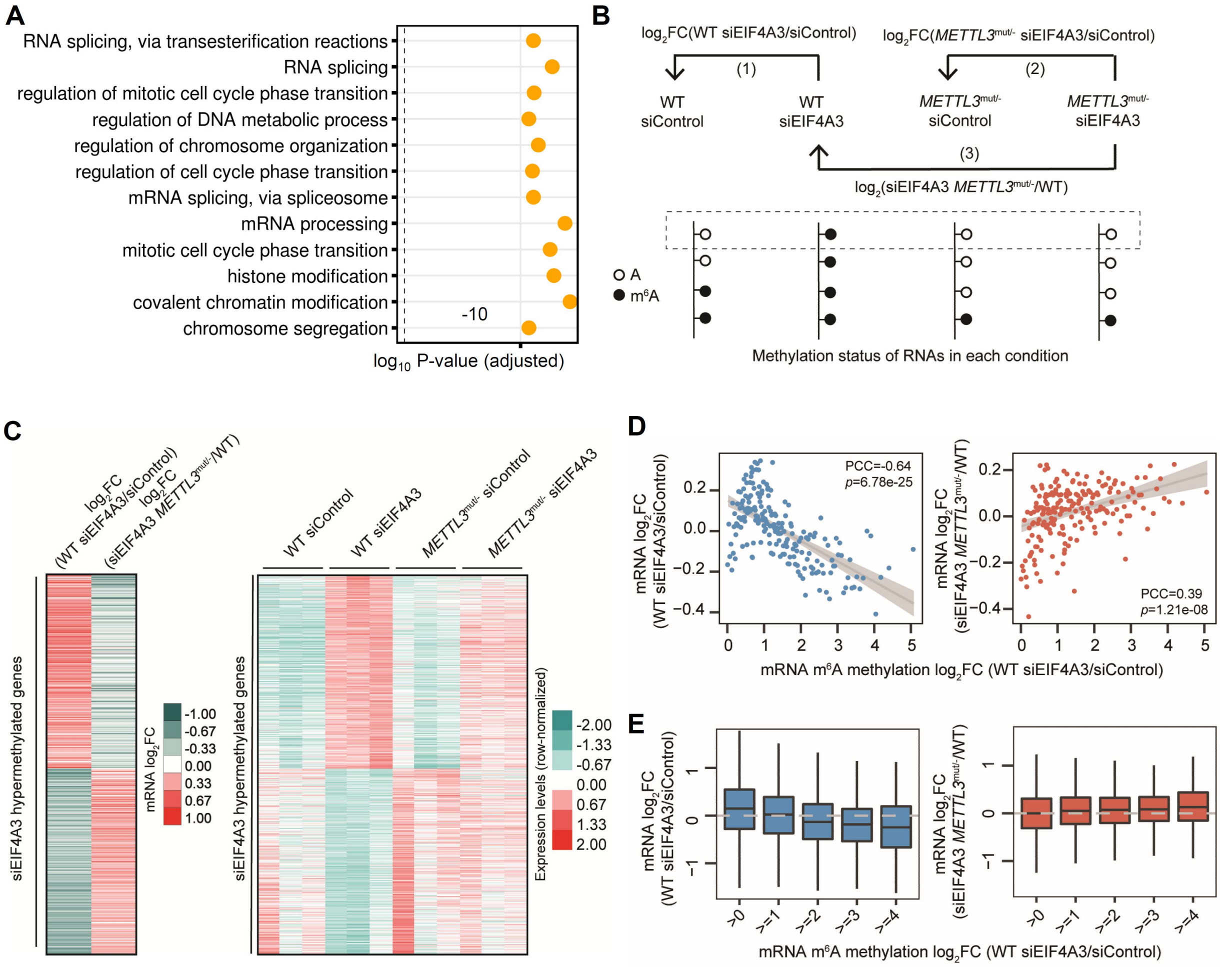

Fig. S18. *METTL3* depletion could at least partially rescue gene expression changes upon EJC KD. (A) Gene ontology term analysis for hypermethylated transcripts upon *EIF4A3* KD, 12 statistically significant terms with lowest *P*-values depicted. (**B**) A schematic model showing comparisons among different conditions. (**C**) Left: heatmap showing fold changes in mRNA abundance upon *EIF4A3* KD ($\log_2$(*siEIF4A3*/siControl)) in WT HeLa cell, and upon METTL3 depletion ($\log_2$(METTL3$^{mut/-}$/WT)) in *siEIF4A3* HeLa cell. mRNA expression level fold changes shown in heatmap was clustered using K-means clustering method with k = 2 and Pearson correlation coefficient as a measure of distance. Right: heatmap showing row wise *Z*-score normalized gene expression level for each of the four conditions shown in (B). Genes with hypermethylated m^{6}A upon EJC knockdown ($\log_2$FC > 0) in HeLa cells were included in the heatmap (n = 9643). Three biological replicates. (**D**) Scatter plots showing the correlation between changes in m^{6}A methylation upon *EIF4A3* KD (WT *siEIF4A3*/siControl) and changes in mRNA abundance changes upon *EIF4A3* KD (WT *siEIF4A3*/siControl) (left) and upon METTL3 depletion in *EIF4A3* KD HeLa cells (*siEIF4A3* METTL3$^{mut/-}$/WT) (right). The hypermethylated mRNAs were categorized into 100 bins based on fold change rank of m^{6}A level upon *EIF4A3* KD in HeLa cells. Pearson Correlation Coefficient (PCC) and *P* values are shown. (**E**) Barplots showing transcript abundance fold changes ($\log_2$FC) of hypermethylated mRNAs upon *EIF4A3* KD (WT *siEIF4A3*/siControl) (left) and upon *METTL3* depletion in *EIF4A3* KD HeLa cells (*siEIF4A3 METTL3*$^{mut/-}$/WT) (right). mRNAs were categorized into three groups according to their

methylation changes upon *EIF4A3* KD in HeLa cells. By “expression”, we are referring to mRNA abundance.

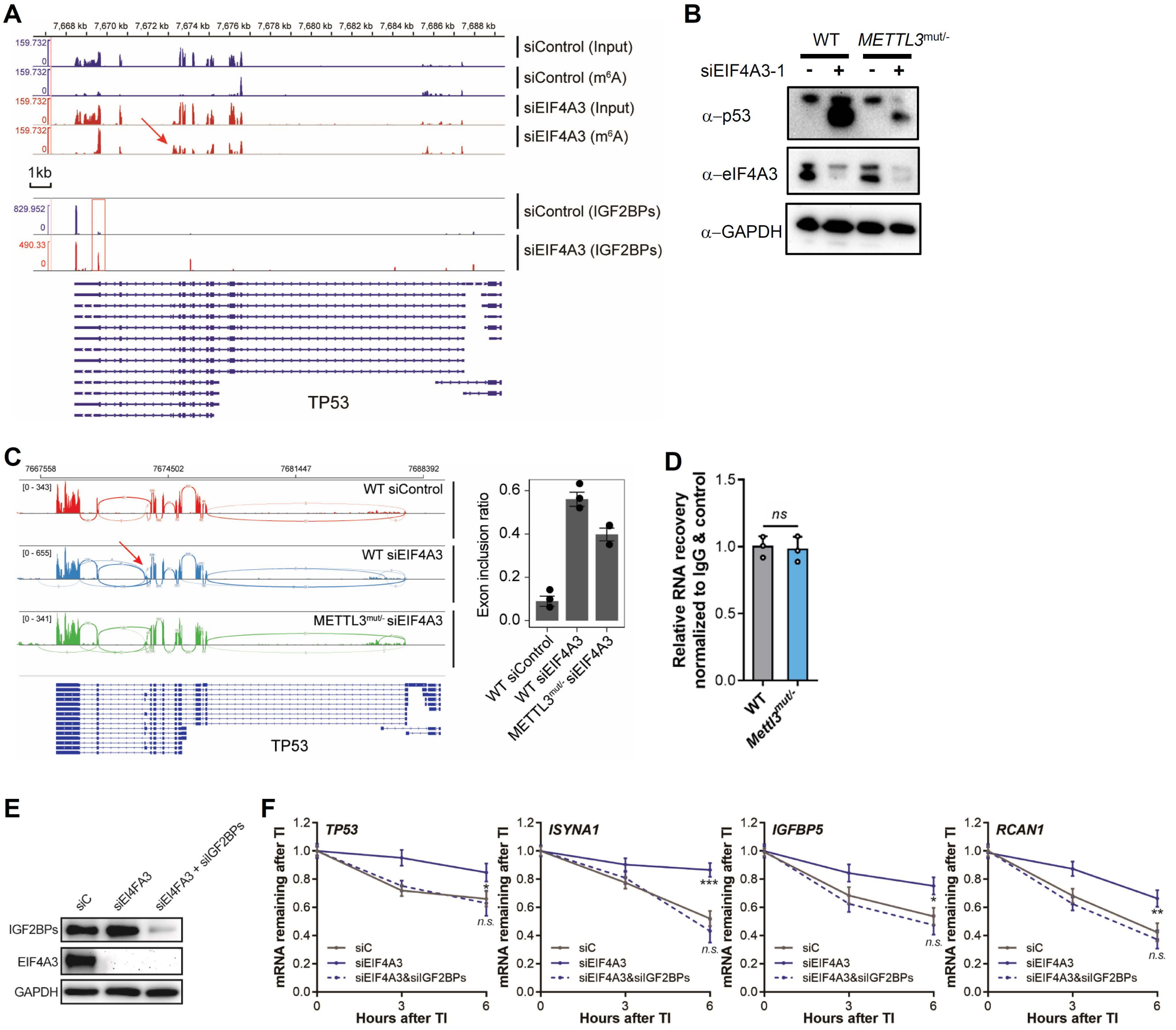

Fig. S19. p53 expression is impacted by EJC KD-mediated m^{6}A hypermethylation. (**A**) IGV profiles showing increased m^{6}A modification levels and IGF2BPs binding at *TP53* genomic loci in control and *EIF4A3* KD HeLa cells. *EIF4A3* KD led to the hypermethylated peaks: one is localized at the start of the last exon (accounted for ~80%, could be isoform-dependent and independent) and another one is localized at internal short exon (accounted for ~20%, exclusively on the shorter isoform, isoform-dependent). **(B)** Western blot of p53 levels upon *EIF4A3* KD in wild-type and *METTL3*$^{mut/-}$ cells, representative image of three biological replicates. We quantified the band intensity in Fig. 4D and found that the longer isoform also upregulated ~30-40% with *EIF4A3* KD. Both the high and low-molecular-weight p53 proteins were downrnegulated when comparing *METTL3-deficient EIF4A3* KD with WT *EIF4A3* KD HeLa cells. IGF2BPs can stabilize both isoforms in *EIF4A3* KD samples, with a greater effect on the short isoform, with effects on both isoforms able to be rescued by METTL3 depletion. Also note that the protein level of the TP53 longer isoform was not obviously changed when comparing *METTL3*$^{mut/-}$ with WT HeLa cells. This is because the longer isomer of *TP53* was almost unmethylated without *EIF4A3* KD, thus *METTL3* KO did not affect its expression. **(C)** IGV profiles showing Sashimi plot of mRNA in control, *EIF4A3* KD and *METTL3*$^{mut/-}$ in *EIF4A3* KD HeLa cells at *TP53* genomic loci (left). Barlot of the exon (arrowed in IGV plot) inclusion ratio in control, *EIF4A3* KD and *METTL3*$^{mut/-}$ *EIF4A3* KD HeLa cells (right). **(D)** Relative RNA recovery after EIF4A3 CLIP.

EIF4A3 binding was normalized to IgG and recovery rates from *METTL3*$^{mut/-}$ were normalized to that of WT HeLa cells. (**E**) Western blots showing the KD efficiency of *EIF4A3* and *IGF2BP1-3*. (**F**) mRNA stability of selected top targets of IGF2BPs in control, *EIF4A3* KD, and KD of both *EIF4A3* and *IGF2BP1-3* (as rescue) in HeLa cells. *P*-values were determined using unpaired two-tailed *t*-tests; *$P < 0.05$, **$P < 0.01$, ***$P < 0.001$, Error bars and means ± SD are shown for indicated group *vs*. siC for $n = 3$.

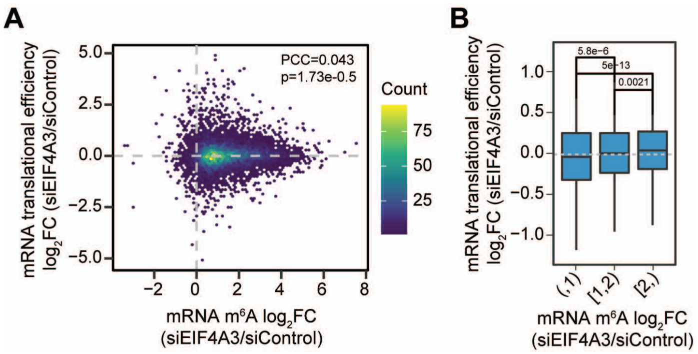

Fig. S20. EJC KD slightly affects translation. (A) Scatter plot of fold changes (log_2FC) in mRNA translation efficiency and fold changes in m^6A methylation level upon *EIF4A3* KD in HeLa cells. PCC and *P* values are shown. (**B**) Barplots showing translational efficiency fold changes (log_2FC) of hypermethylated mRNAs upon *EIF4A3* KD in HeLa cells. mRNAs were categorized into three groups according to their methylation changes upon *EIF4A3* KD in HeLa cells. *P* values obtained from Wilcoxon rank sum test are shown.

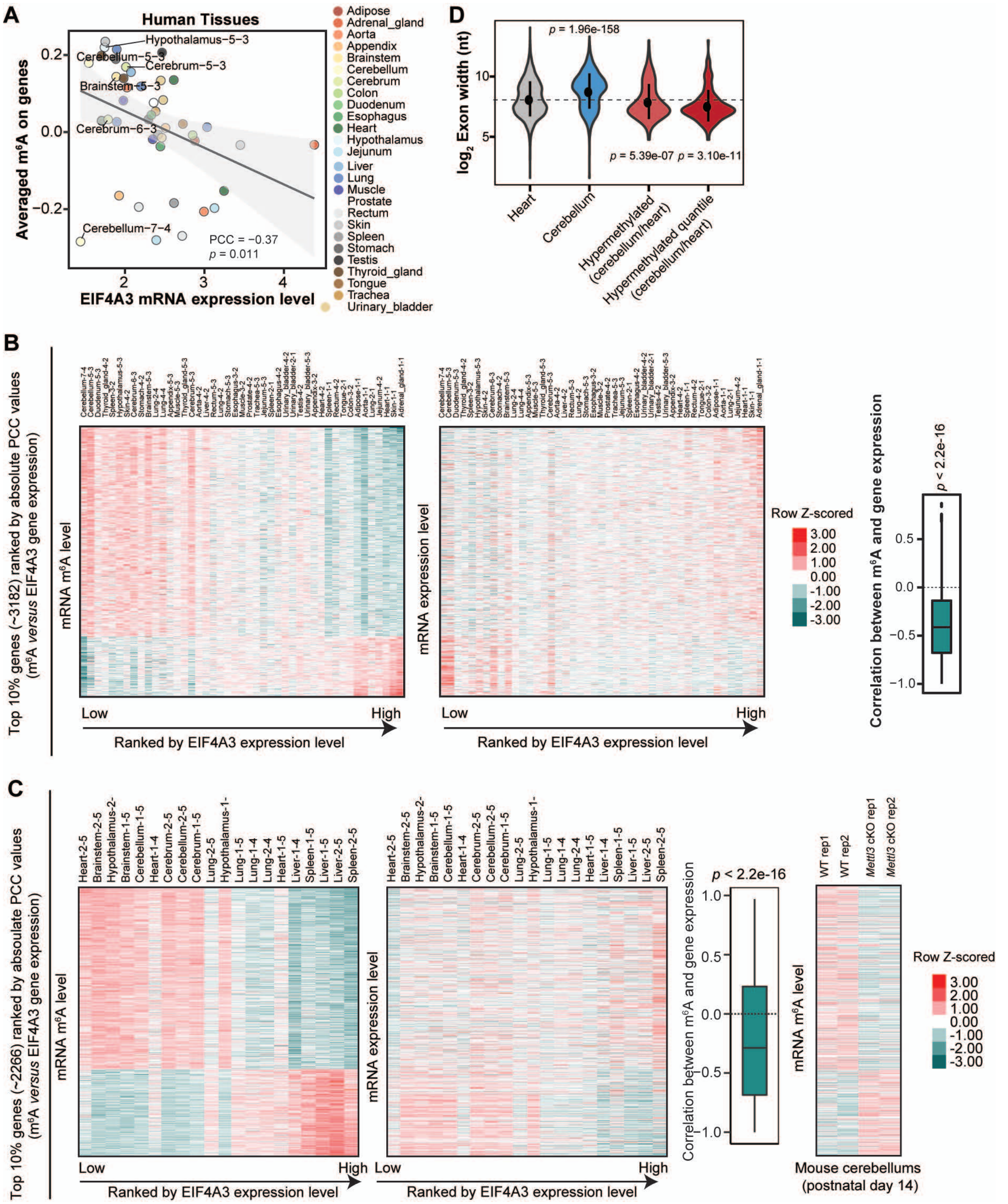

Fig. S21. EJCs regulate m^{6}A deposition and mRNA stability across tissues. (**A**) The negative correlation between *EIF4A3* mRNA expression and mRNA m^{6}A level across human tissues (CRA001315). PCC and *P* value are shown. (**B**) Heatmap of mRNA m^{6}A level (left) or abundance (right) across human tissues: Columns represent genes, rows represent human tissues and entries are mRNA m^{6}A level (left) or abundance (right). mRNAs with m^{6}A level highly correlated with *EIF4A3* mRNA expression across human tissues were selected (the top 10% genes ranked by |PCC| values). Columns were ranked according to the correlation (PCC) between mRNA m^{6}A level and

EIF4A3 expression levels across human tissues. Rows were ranked according to the *EIF4A3* expression levels across human tissues. Boxplot (bottom) is the summary of the negative correlation between m^6A and mRNA expression level for genes with m^6A methylation negatively correlated with *EIF4A3* expression levels across human tissues shown in the heatmap. (**C**) Heatmap of mRNA m^6A level (left) or abundance (middle) across mouse tissues: Rows represented genes, columns represented mouse tissues and entries were mRNA m^6A level (first panel) or abundance (second panel). mRNAs with m^6A level highly correlated with *EIF4A3* gene expression level across mouse tissues (the top 10% genes ranked by |PCC| value) were selected. Rows were ranked according to the correlation (PCC) between mRNA m^6A level and *EIF4A3* gene expression across mouse tissues. Columns were ranked according to the expression level of *EIF4A3* gene expression across mouse tissues. Third panel: Boxplot showing the negative correlation between m^6A and mRNA expression level for each gene with m^6A methylation negatively correlated with *EIF4A3* expression in left panel. P value obtained from Wilcoxon rank sum test is shown. Forth panel: heatmap of mRNA m^6A level in WT and *Mettl3* KO mouse cerebellum. (**D**) Exon length of all m^6A peaks residing within internal exons in heart, cerebellum, and exon lengths of m^6A hypermethylated regions within internal exons comparing human cerebellum with human heart. Dot and bar represent the median and interquartile range, Wilcoxon rank sum test of indicated groups *vs.* heart all m^6A peaks. For (B) and (C), the color scale on the graph represents the row wise *z*-scored normalized m^6A (*left*) or gene expression (*right*) level across different human or mouse tissues.

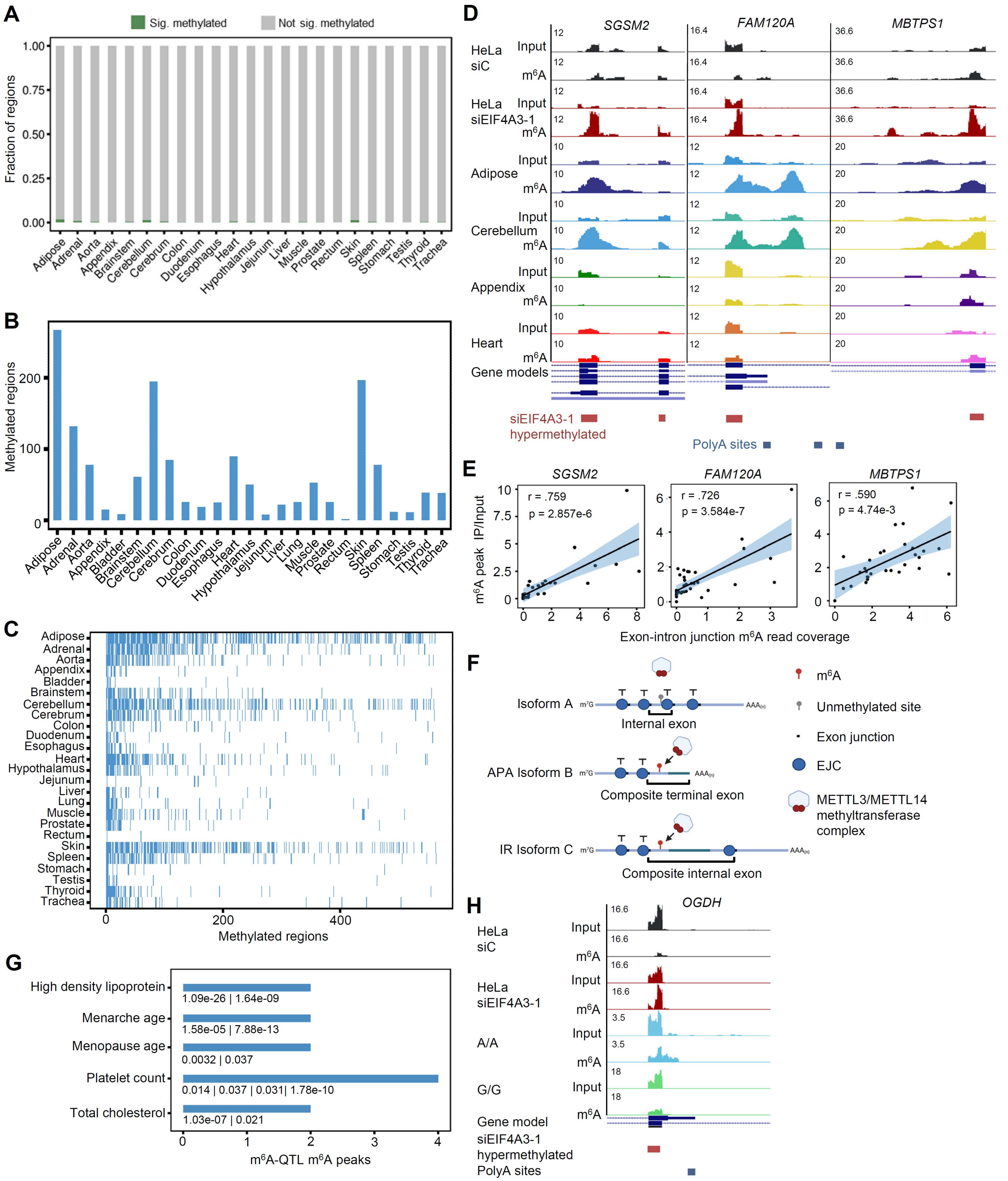

Fig. S22. The EJC-mediated m^{6}A suppression contributes to tissue-specific m^{6}A methylation and inter-individual m^{6}A variation associated with complex traits. (**A**) Fractions of EJC-suppressed m^{6}A regions that are significantly methylated or not significantly methylated in different human tissues. (**B**) Numbers of the EIF4A3-suppressed regions in HeLa cells that are methylated in different human tissues. (**C**) The methylation status (methylated *versus* unmethylated) of the EIF4A3-suppressed regions in HeLa cells across different human tissues. (**D**)

Read coverage of Input and m^6A-IP samples for *SGSM2, FAM120A* and *MBTPS1* in control HeLa cells, *siEIF4A3*-1 HeLa cells and representative tissues. Gene models and annotated polyadenylation sites are shown below. (**E**) Positive correlations between read coverage in m^6A-IP samples across exon-intron junction regions and IP/input enrichment ratio of m^6A peak located at exon-intron junction regions for the above three transcripts, Pearson correlation. Each dot on the plot represents a sequencing sample, with some samples being from the same tissue (biological replicates). (**F**) Schematic model for suppression of methylation within the internal exon of the canonical isoform (Isoform A), escape from suppression at the corresponding site in an isoform with the escaping site residing in a longer composite terminal exon due to intronic polyadenylation (APA Isoform B), and escape from suppression at the corresponding site in an isoform with the escaped site residing in a longer composite internal exon due to intron retention (IR Isoform C). Intron retention at these sites converts an average-length internal exon into a long composite internal exon that contains two exons and their intervening intronic sequence. Similarly, APA at these sites converts an average-length internal exon into a longer composite terminal exon that contains both the internal exon and downstream intronic sequence. (**G**) Numbers of the EIF4A3-suppressed regions (m^6A peaks $\geq$ 2) overlapped with m^6A-QTL m^6A peaks in Yoruba lymphoblastoid cell lines that are significantly associated with specified complex traits. *P* value for each m^6A peak is shown under the bar. (**H**) Read coverage of Input and m^6A-IP samples in control HeLa cells, *siEIF4A3*-1 HeLa cells and Yoruba lymphoblastoid cell lines NA18498 and NA18507. NA18498 is genotype A/A at the *OGDH* m^6A-QTL SNP (rs740040). NA18507 is genotype G/G. Gene models and annotated polyadenylation sites are shown below.

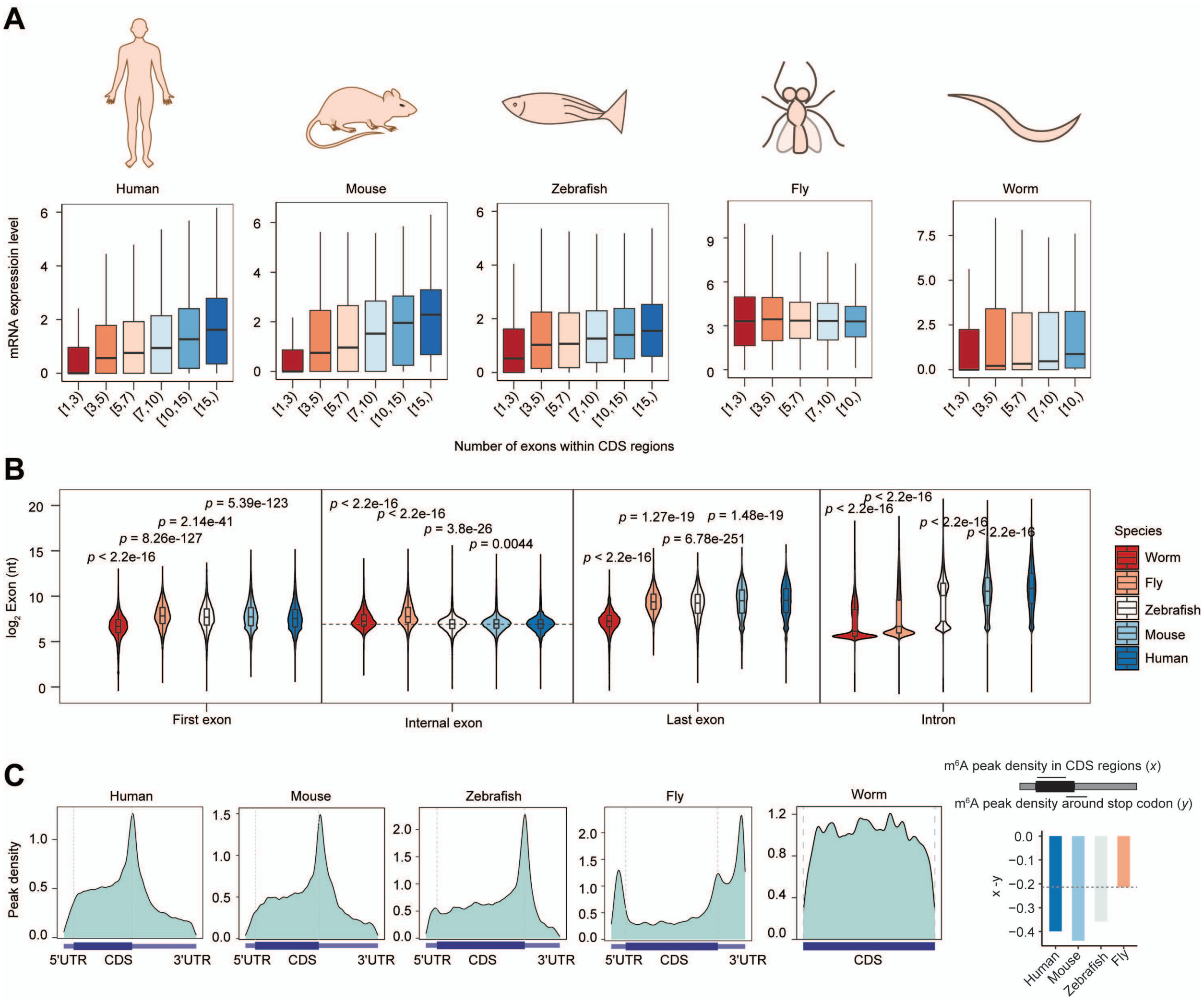

Fig. S23. Exon-junction architecture regulates mRNA abundance through m^{6}A methylation across organisms. (**A**) Correlation between mRNA expression level and exon number within CDS regions across different species. No obvious correlation between exon numbers with transcript levels in worm and fly, suggesting lack of the EJC-mediated mRNA stability regulation. (**B**) Exon lengths distribution across different species. Dot and bar represent median and interquartile range. Wilcoxon rank sum test of indicated groups vs. human exon length. The average internal exon length of worm and fly in general is much longer than mouse/human (p < 2.24e-16). Lower organisms such as drosophila or worm do not have YTHDF2 and thus do not appear to possess the EJC-mediated mRNA stability regulation. These data suggest that through evolution, the correlation between exon-intron architecture and mRNA stability holds from fish to human but not fly and worm. (**C**) Metagene plot of m^{6}A peaks across different species and barplot of the difference in density of m^{6}A peaks between CDS regions and those around stop codon regions as shown in the *top* illustration. When comparing methylation around the stop codon to methylation within the coding sequence (CDS), we found that m^{6}A methylation within the CDS in flies is less suppressed compared to that in human, mouse, and zebrafish. This observation might be due to longer internal exons found in flies compared to those in human, mouse, or zebrafish.

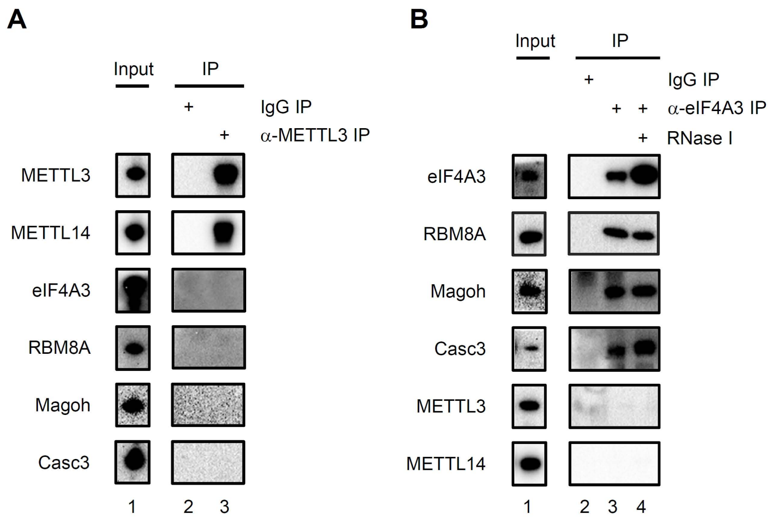

Fig. S24. EJCs and METTL3/METTL14 do not co-immunoprecipitate. (A) Western blots of EJCs and METTL3/METTL14 proteins from total cell extracts (input) or upon immunoprecipitation with IgG or α−METTL3. **(B)** Western blots of EJCs and METTL3/METTL14 proteins from total cell extracts (input) or upon immunoprecipitation with IgG or α−EIF4A3, with or without RNase I digestion.

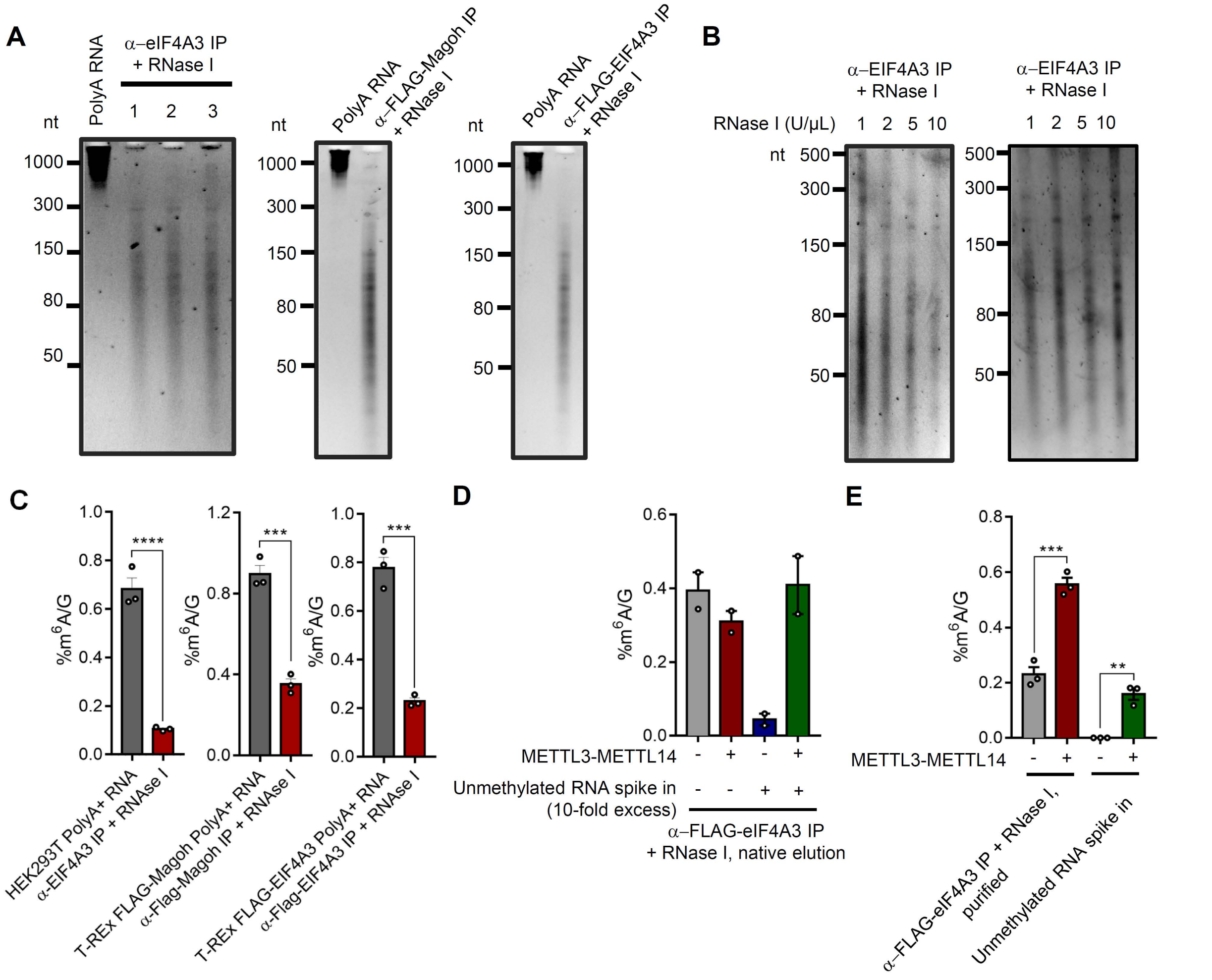

Fig. S25. EJCs protect long RNA footprints from cellular m^{6}A deposition and in vitro m^{6}A deposition. (A) Length distributions of bulk polyadenylated RNA and RNase I-resistant EJC footprints. Length distribution of polyadenylated RNA (lane 1) and EJC footprints obtained from α–EIF4A3 immunoprecipitation and RNase I digestion in HEK293T cells (lane 2-4) (left). Length distribution of polyadenylated RNA (lane 1) and EJC footprints obtained from α–FLAG immunoprecipitation and RNase I digestion (lane 2) in TREx cells expressing FLAG-tagged Magoh (middle). Length distribution of polyadenylated RNA (lane 1) and EJC footprints obtained from α–FLAG immunoprecipitation and RNase I digestion (lane 2) in TREx cells expressing FLAG-tagged EIF4A3 (right). **(B)** Length distributions of bulk polyadenylated RNA and RNase I-resistant EJC footprints under varying RNase concentrations. Equal reaction volumes loaded in each lane (left), loaded reaction volumes adjusted to equalize intensity of signal in each lane (right). Consistent with previous reports, we observed the presence of long EJC-protected RNA footprints up to 150 nucleotides in length. **(C)** LC-MS/MS measurement of m^{6}A levels of bulk polyadenylated RNA and RNase I-resistant EJC footprints. m^{6}A levels of polyadenylated RNA and EJC footprints obtained from α–EIF4A3 immunoprecipitation and RNase I digestion in HEK293T cells (left). m^{6}A levels of polyadenylated RNA and EJC footprints obtained from α–FLAG immunoprecipitation and RNase I digestion in TREx cells expressing FLAG-tagged Magoh (middle). m^{6}A levels of polyadenylated RNA and EJC footprints obtained from α–FLAG immunoprecipitation and RNase I digestion in TREx cells expressing FLAG-tagged EIF4A3

(right). Three biological replicates, mean ± SEM, two-tailed T-test, ****P = 0.0002, ***P < 0.0005. **(D)** LC-MS/MS measurement of m^{6}A levels of RNase I-resistant EJC footprints obtained by native elution of RNA-protein complexes and subsequent *in vitro* methylation with recombinant METTL3-METTL14. RNA-protein complexes were obtained by α−FLAG immunoprecipitation and RNase I digestion in TREx cells expressing FLAG-tagged EIF4A3, and then competitively eluted with FLAG peptide in methylation reaction buffer. For the right two bars, unmethylated RNA (in vitro transcribed MPm^{6}A library RNA) was spiked into the reactions in 10-fold excess to the amount of eluted EJC-bound RNA. n= 2, mean ± SEM, two-tailed T-test, *P = 0.045 **(E)** LC-MS/MS measurement of m^{6}A levels of Trizol-purified, deproteinized RNase I-resistant EJC footprints subjected to in vitro methylation with recombinant METTL3-METTL14 with the same reaction conditions used in (D).n = 3, mean ± SEM, two-tailed T-test, ***P = 0.0008, **P = 0.0016.

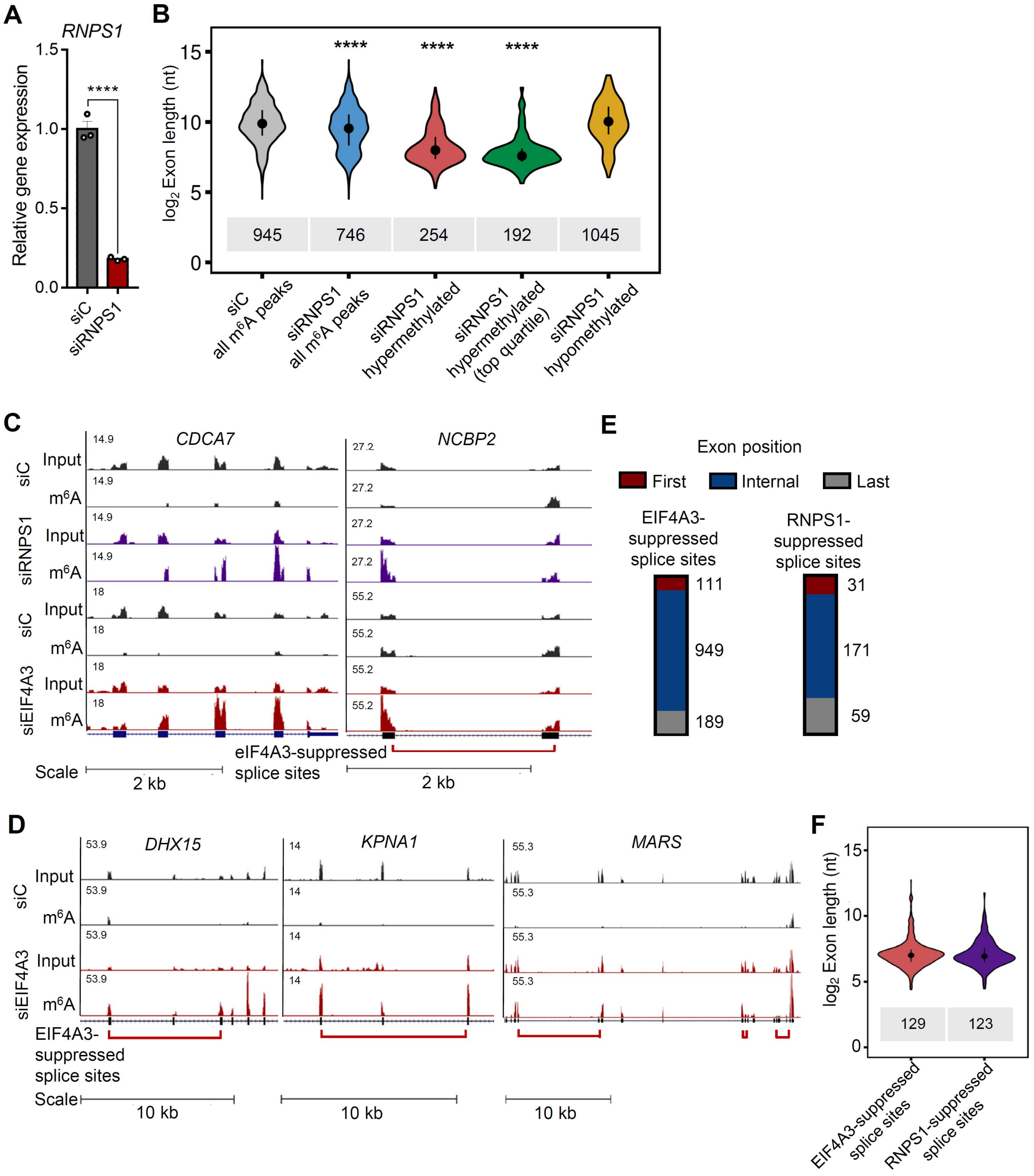

Fig. S26. EJC peripheral factor RNPS1 preferentially suppresses m^{6}A methylation within average-length internal in mRNA coding sequences. **(A)** *RNPS1* knockdown efficiency, normalized to siC, mean ± SEM, two-tailed t-test, *****P* < 0.0001. Three biological replicates **(B)** Exon lengths of all m^{6}A peaks residing within internal exons in control KD and *RNPS1* KD, and m^{6}A hypermethylated and hypomethylated regions within internal exons upon *RNPS1* KD in HeLa cells. Dot and bar represent median and interquartile range, Wilcoxon rank sum test of indicated group *vs.* siC all m^{6}A peaks, *****P* < 2.2e-16. **(C)** Input and m^{6}A-IP read coverage at *CDCA7* and *NCBP2* upon *RNPS1* KD, *EIF4A3* KD, and non-targeting control KD in HeLa cells. **(D)** Input and m^{6}A-IP read coverage at *DHX15*, *KPNA1* and *MARS* upon *EIF4A3* KD and non-targeting control KD in HeLa cells. Red bracket indicates an EIF4A3-suppressed splice variant, with ends of bracket indicating the suppressed splice junctions. **(E)** Number of EIF4A3-suppressed and RNPS1-suppressed splice sites that unambiguously reside within first, internal or last exons. **(F)** Exon

lengths of EIF4A3-suppressed (median = 129 nt) and RNPS1-suppressed splice sites (median = 123 nt) that reside in internal exons. Dot and bar represent median and interquartile range.

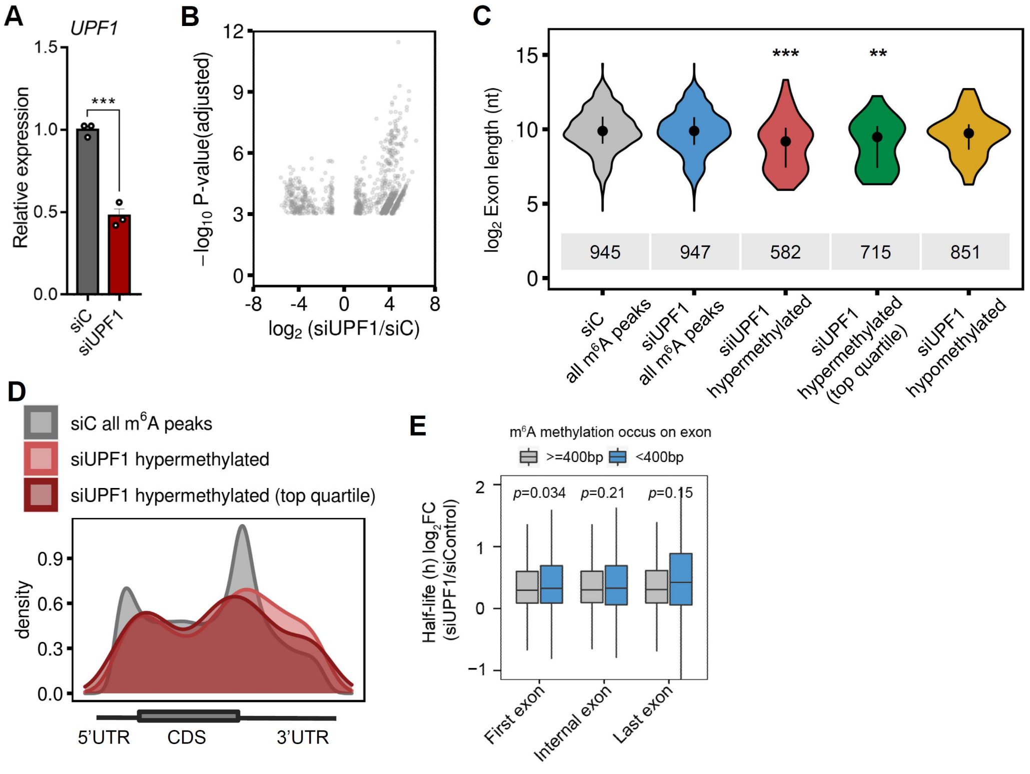

Fig. S27. Nonsense-mediated decay factor UPF1 does not preferentially suppress m^{6}A methylation within internal exons close to average length in mRNA coding sequences. (A) *UPF1* knockdown efficiency, normalized to siC, mean ± SEM, two-tailed T-test, ***P < 0.001. Three biological replicates **(B)** Differentially methylated regions upon *UPF1* KD in HeLa cells (FDR<.1, |log$_2$FC|>1), three biological replicates. **(C)** Exon lengths of all m^{6}A peaks within internal exons in control and upon *UPF1* KD, and exon lengths of m^{6}A hypermethylated and hypomethylated regions within internal exons upon *UPF1* KD. Dot and bar represent median and interquartile range, Wilcoxon rank sum test of specified group vs. siC all m^{6}A peaks, ***P = 1.8e-7, **P = .0065. **(D)** Metagenes of all m^{6}A peaks in control KD, significantly m^{6}A hypermethylated regions, and the top quartile of significantly m^{6}A hypermethylated regions upon *UPF1* KD. **(E)** Boxplot showing fold-changes of mRNA half-life upon *UPF1* KD in HepG2 cells. m^{6}A-modified mRNAs (GSE90642) were categorized according to the length of their first, internal, and last exons, respectively. *P* values obtained from Wilcoxon rank sum test are shown.

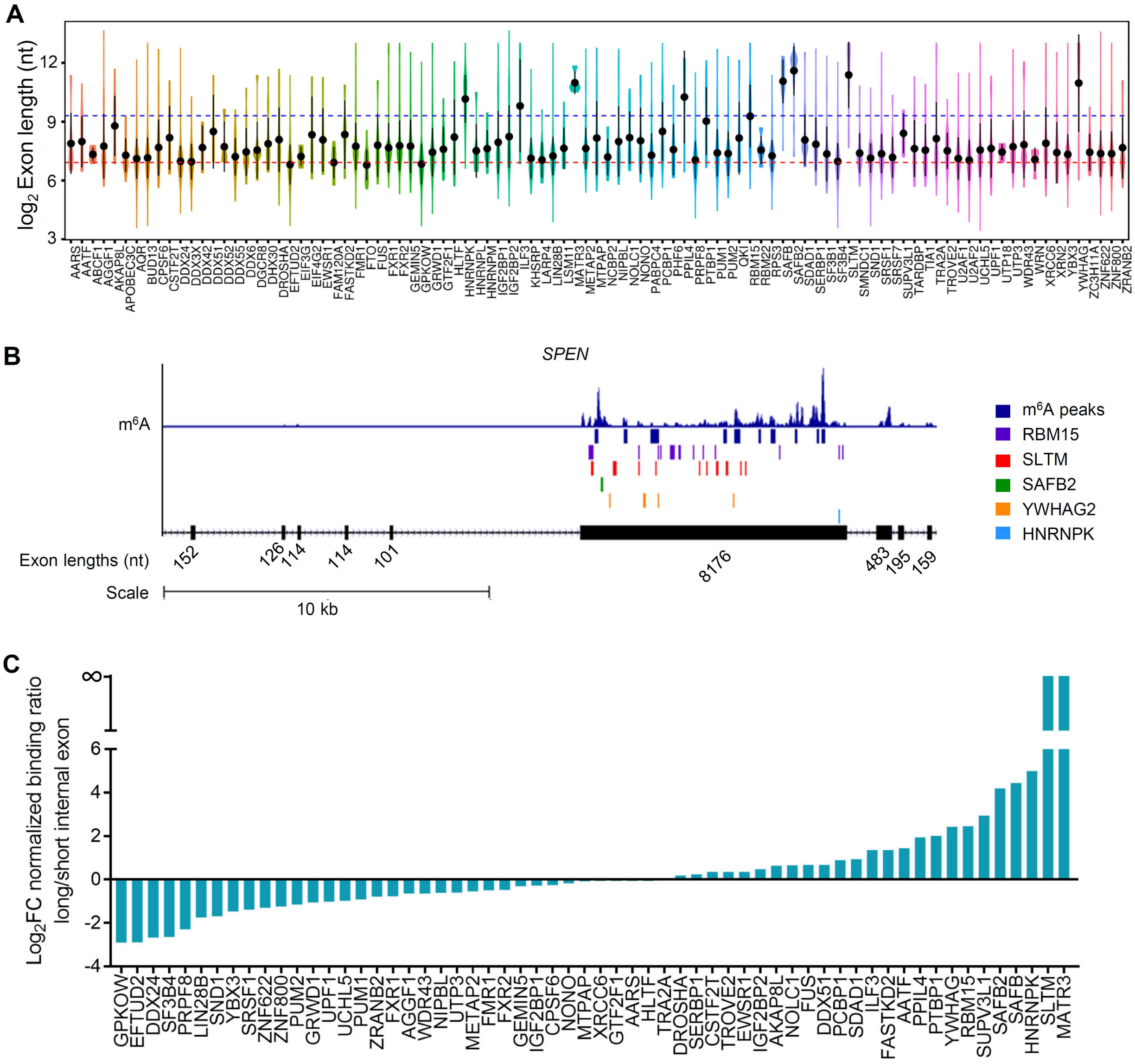

Fig. S28. A subset of RBPs preferentially bind long internal exons. (A) Lengths of internal exons bound by 97 RBPs in K562 cells. Red line indicates the median exon size of all expressed internal exons in K562 cells (122 nt), blue line indicates the median exon size for internal exons bound by RBM15, a m^{6}A methyltransferase accessory subunit (626 nt). Dot and bar represent mean ± SD. **(B)** m^{6}A methylation and RBP binding sites on SPEN mRNA. **(C)** Log$_2$ fold changes in normalized binding ratio of RBPs between long and short internal exons. RBPs that are added on before EJC deposition would not be expected to prefer long exons. Indeed, we find that RBPs like EFTUD2, SF3B4, which are spliceosome components, prefer to bind short internal exons once normalized for number of nucleotides/exon.

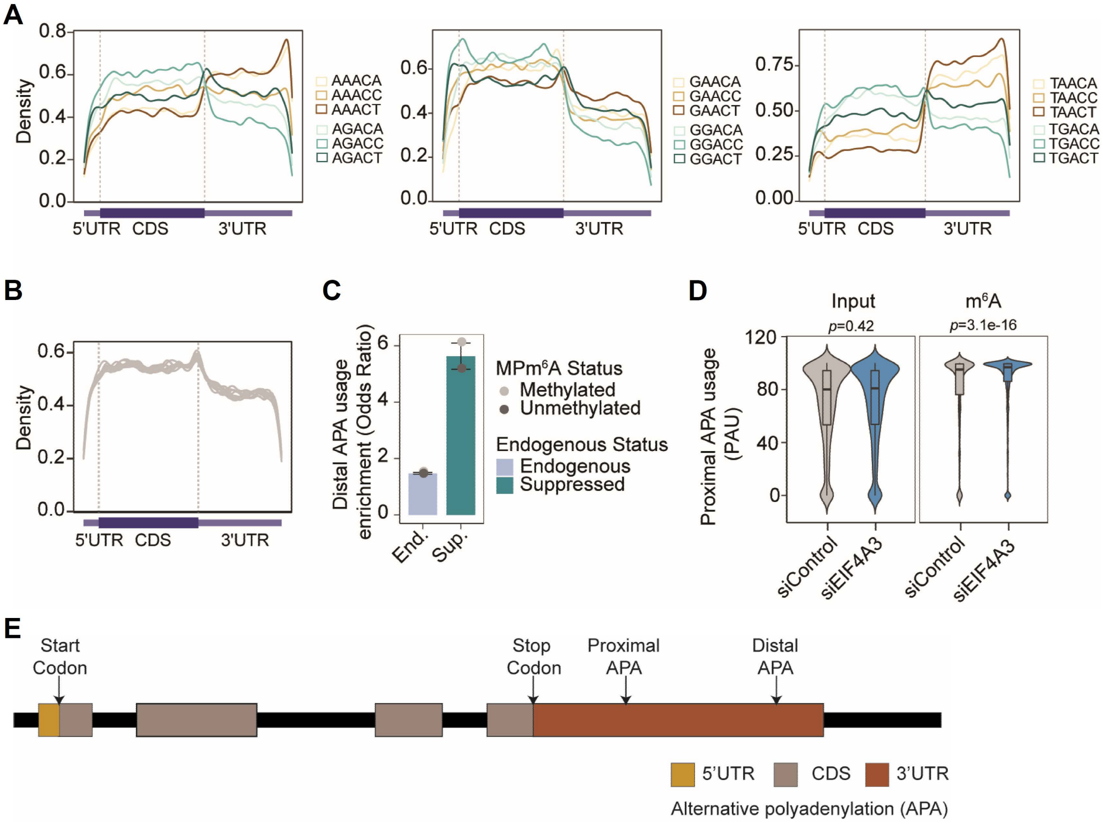

Fig. S29. Alternative polyadenylation (APA) may function as an m^{6}A suppressor in the 3'UTR. (**A**) Metagene plot of each DRACH motif in the human transcriptome. (**B**) Metagene plot of DRACH motifs sampling without replacement ten times from human transcriptome. Sampling without replacement was performed with weights for each DRACH according to their frequency in m^{6}A modified DARCH sites obtained from miCLIP in the HEK293 cell (GSE63753). (**C**) The enrichment of distal APA usage of the endogenous locations of endogenously methylated and unmethylated MPm^{6}A sequences that were methylated or unmethylated in the MPm^{6}A assay. The distal APA usage was quantified by QAPA package (*79*). The enrichment score was quantified by odds ratio in Fisher Exact test. (**D**) Proximal APA usage (PAU) in Input and m^{6}A-IP samples upon *EIF4A3* KD in HeLa cells. The proximal APA usage was quantified by QAPA package (*79*). Dot and bar represent median and interquartile range. *P* values obtained from Wilcoxon rank sum test are shown. (**E**) Diagram showing 3′UTR APA. Isoforms with alternative 3′ UTRs result from the use of different PASs in the 3′UTR. The two shown PASs are the proximal PAS and the distal PAS.

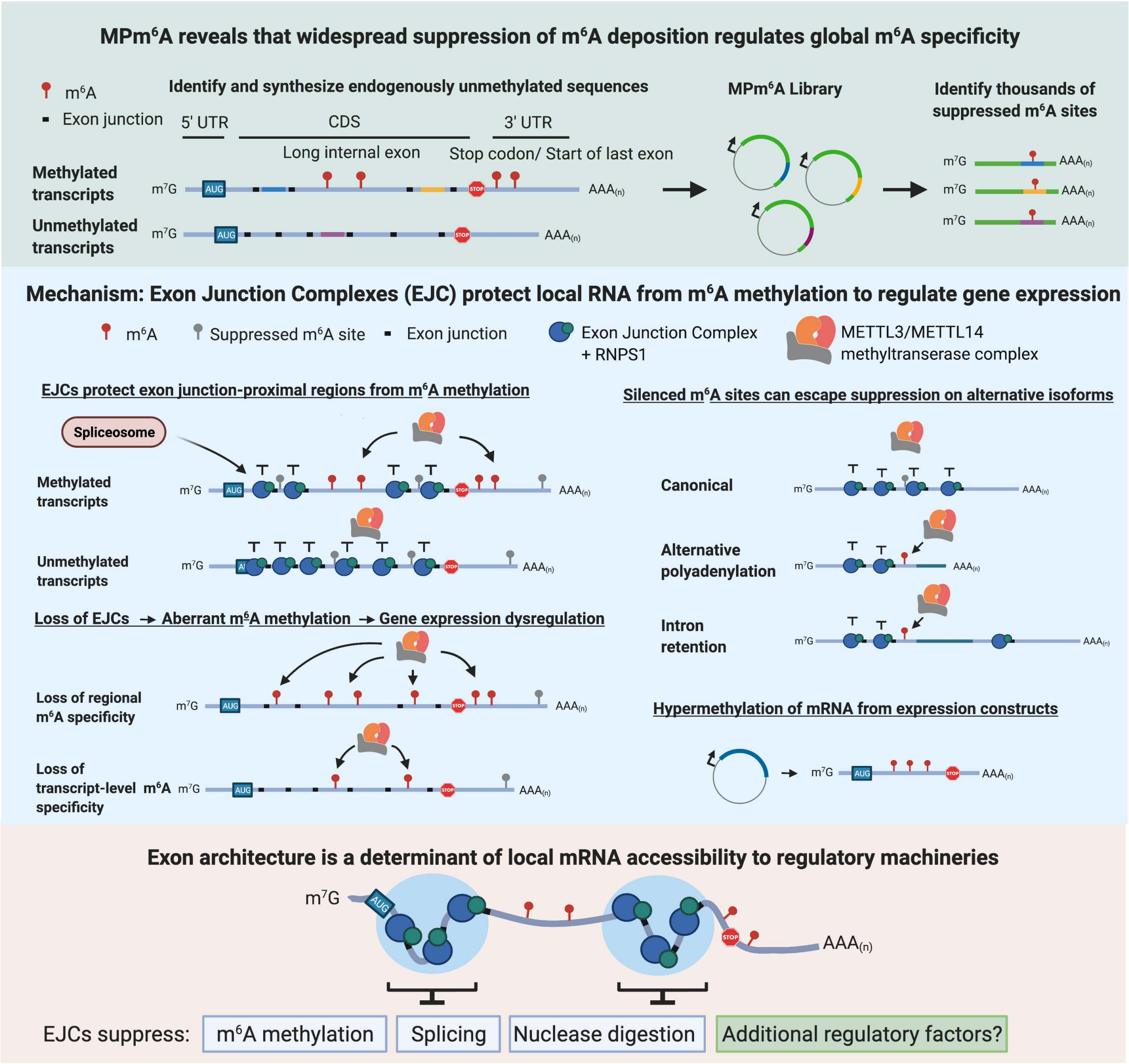

Fig. S30. MPm^{6}A identifies EJCs as m^{6}A suppressors. Schematic model describing use of MPm^{6}A to discover that widespread suppression of m^{6}A controls global m^{6}A epitranscriptome specificity, and mechanism by which pre-mRNA splicing and subsequent exon junction complex deposition suppress local m^{6}A methylation. Transcripts with altered exon architecture due to alternative pre-mRNA processing or expression from unspliced expression constructs can display altered m^{6}A patterns due to altered/absent EJC deposition by spliceosomes. Schematic model depicting exon architecture as a broad determinant of local mRNA accessibility to RNA regulatory machineries *via* packaging of exon-junction proximal RNA by RNPS1-associated EJCs and related binding proteins.

Table S1 to S6

Table S1. Knockdown efficiency of *EIF4A3, RBM8A, RNPS1, UPF1* in m^6A-meRIP-seq experiments.

Table S2. EIF4A3-suppressed and RNPS1-suppressed splice sites in proximity to EIF4A3-suppressed m^6A regions.

Table S3. Tissue m^6A peaks that contain EJC-suppressed m^6A regions and span exon-intron boundaries.

Table S4. Retained intron tissue m^6A peaks that contain EJC-suppressed m^6A regions.

Table S5. m^6A-QTL-associated m^6A peaks that contain EJC-suppressed m^6A regions.

Table S6. Oligonucleotide sequences, antibodies, and other reagents used in this study.

References and Notes

1. M. Frye, B. T. Harada, M. Behm, C. He, RNA modifications modulate gene expression during development. *Science* **361**, 1346–1349 (2018). doi:10.1126/science.aau1646 Medline

2. W. V. Gilbert, T. A. Bell, C. Schaening, Messenger RNA modifications: Form, distribution, and function. *Science* **352**, 1408–1412 (2016). doi:10.1126/science.aad8711 Medline

3. I. A. Roundtree, M. E. Evans, T. Pan, C. He, Dynamic RNA modifications in gene expression regulation. *Cell* **169**, 1187–1200 (2017). doi:10.1016/j.cell.2017.05.045 Medline

4. P. C. He, C. He, m^6A RNA methylation: From mechanisms to therapeutic potential. *EMBO J.* **40**, e105977 (2021). doi:10.15252/embj.2020105977 Medline

5. D. Dominissini, S. Moshitch-Moshkovitz, S. Schwartz, M. Salmon-Divon, L. Ungar, S. Osenberg, K. Cesarkas, J. Jacob-Hirsch, N. Amariglio, M. Kupiec, R. Sorek, G. Rechavi, Topology of the human and mouse m^6A RNA methylomes revealed by m^6A-seq. *Nature* **485**, 201–206 (2012). doi:10.1038/nature11112 Medline

6. S. Ke, E. A. Alemu, C. Mertens, E. C. Gantman, J. J. Fak, A. Mele, B. Haripal, I. Zucker-Scharff, M. J. Moore, C. Y. Park, C. B. Vågbø, A. Kusśnierczyk, A. Klungland, J. E. Darnell Jr., R. B. Darnell, A majority of m^6A residues are in the last exons, allowing the potential for 3′ UTR regulation. *Genes Dev.* **29**, 2037–2053 (2015). doi:10.1101/gad.269415.115 Medline

7. I. Barbieri, K. Tzelepis, L. Pandolfini, J. Shi, G. Millán-Zambrano, S. C. Robson, D. Aspris, V. Migliori, A. J. Bannister, N. Han, E. De Braekeleer, H. Ponstingl, A. Hendrick, C. R. Vakoc, G. S. Vassiliou, T. Kouzarides, Promoter-bound METTL3 maintains myeloid leukaemia by m^6A-dependent translation control. *Nature* **552**, 126–131 (2017). doi:10.1038/nature24678 Medline

8. A. Bertero, S. Brown, P. Madrigal, A. Osnato, D. Ortmann, L. Yiangou, J. Kadiwala, N. C. Hubner, I. R. de Los Mozos, C. Sadée, A.-S. Lenaerts, S. Nakanoh, R. Grandy, E. Farnell, J. Ule, H. G. Stunnenberg, S. Mendjan, L. Vallier, The SMAD2/3 interactome reveals that TGFβ controls m^6A mRNA methylation in pluripotency. *Nature* **555**, 256–259 (2018). doi:10.1038/nature25784 Medline

9. L. Fish, A. Navickas, B. Culbertson, Y. Xu, H. C. B. Nguyen, S. Zhang, M. Hochman, R. Okimoto, B. D. Dill, H. Molina, H. S. Najafabadi, C. Alarcón, D. Ruggero, H. Goodarzi, Nuclear TARBP2 drives oncogenic dysregulation of RNA splicing and decay. *Mol. Cell* **75**, 967–981.e9 (2019). doi:10.1016/j.molcel.2019.06.001 Medline

10. B. Slobodin, R. Han, V. Calderone, J. A. F. O. Vrielink, F. Loayza-Puch, R. Elkon, R. Agami, Transcription impacts the efficiency of mRNA translation via co-transcriptional N^6-adenosine methylation. *Cell* **169**, 326–337.e12 (2017). doi:10.1016/j.cell.2017.03.031 Medline

11. H. Huang, H. Weng, K. Zhou, T. Wu, B. S. Zhao, M. Sun, Z. Chen, X. Deng, G. Xiao, F. Auer, L. Klemm, H. Wu, Z. Zuo, X. Qin, Y. Dong, Y. Zhou, H. Qin, S. Tao, J. Du, J. Liu, Z. Lu, H. Yin, A. Mesquita, C. L. Yuan, Y.-C. Hu, W. Sun, R. Su, L. Dong, C. Shen, C. Li, Y. Qing, X. Jiang, X. Wu, M. Sun, J.-L. Guan, L. Qu, M. Wei, M. Müschen, G. Huang, C. He, J. Yang, J. Chen, Histone H3 trimethylation at lysine 36 guides m^6A RNA

modification co-transcriptionally. *Nature* **567**, 414–419 (2019). doi:10.1038/s41586-019-1016-7 Medline

12. Z. Zhang, K. Luo, Z. Zou, M. Qiu, J. Tian, L. Sieh, H. Shi, Y. Zou, G. Wang, J. Morrison, A. C. Zhu, M. Qiao, Z. Li, M. Stephens, X. He, C. He, Genetic analyses support the contribution of mRNA N^6-methyladenosine (m^6A) modification to human disease heritability. *Nat. Genet.* **52**, 939–949 (2020). doi:10.1038/s41588-020-0644-z Medline

13. E. L. Van Nostrand, P. Freese, G. A. Pratt, X. Wang, X. Wei, R. Xiao, S. M. Blue, J.-Y. Chen, N. A. L. Cody, D. Dominguez, S. Olson, B. Sundararaman, L. Zhan, C. Bazile, L. P. B. Bouvrette, J. Bergalet, M. O. Duff, K. E. Garcia, C. Gelboin-Burkhart, M. Hochman, N. J. Lambert, H. Li, M. P. McGurk, T. B. Nguyen, T. Palden, I. Rabano, S. Sathe, R. Stanton, A. Su, R. Wang, B. A. Yee, B. Zhou, A. L. Louie, S. Aigner, X.-D. Fu, E. Lécuyer, C. B. Burge, B. R. Graveley, G. W. Yeo, A large-scale binding and functional map of human RNA-binding proteins. *Nature* **583**, 711–719 (2020). doi:10.1038/s41586-020-2077-3 Medline

14. H. Le Hir, J. Saulière, Z. Wang, The exon junction complex as a node of post-transcriptional networks. *Nat. Rev. Mol. Cell Biol.* **17**, 41–54 (2016). doi:10.1038/nrm.2015.7 Medline

15. V. Boehm, N. H. Gehring, Exon junction complexes: Supervising the gene expression assembly line. *Trends Genet.* **32**, 724–735 (2016). doi:10.1016/j.tig.2016.09.003 Medline

16. V. Boehm, T. Britto-Borges, A.-L. Steckelberg, K. K. Singh, J. V. Gerbracht, E. Gueney, L. Blazquez, J. Altmüller, C. Dieterich, N. H. Gehring, Exon junction complexes suppress spurious splice sites to safeguard transcriptome integrity. *Mol. Cell* **72**, 482–495.e7 (2018). doi:10.1016/j.molcel.2018.08.030 Medline

17. L. Blazquez, W. Emmett, R. Faraway, J. M. B. Pineda, S. Bajew, A. Gohr, N. Haberman, C. R. Sibley, R. K. Bradley, M. Irimia, J. Ule, Exon junction complex shapes the transcriptome by repressing recursive splicing. *Mol. Cell* **72**, 496–509.e9 (2018). doi:10.1016/j.molcel.2018.09.033 Medline

18. G. Singh, A. Kucukural, C. Cenik, J. D. Leszyk, S. A. Shaffer, Z. Weng, M. J. Moore, The cellular EJC interactome reveals higher-order mRNP structure and an EJC-SR protein nexus. *Cell* **151**, 750–764 (2012). doi:10.1016/j.cell.2012.10.007 Medline

19. W.-C. Lee, B.-H. Hou, C.-Y. Hou, S.-M. Tsao, P. Kao, H.-M. Chen, Widespread exon junction complex footprints in the RNA degradome mark mRNA degradation before steady state translation. *Plant Cell* **32**, 904–922 (2020). doi:10.1105/tpc.19.00666 Medline

20. T. Ø. Tange, T. Shibuya, M. S. Jurica, M. J. Moore, Biochemical analysis of the EJC reveals two new factors and a stable tetrameric protein core. *RNA* **11**, 1869–1883 (2005). doi:10.1261/rna.2155905 Medline

21. Y. Xiao, Y. Wang, Q. Tang, L. Wei, X. Zhang, G. Jia, An elongation- and ligation-based qPCR amplification method for the radiolabeling-free detection of locus-specific N^6-methyladenosine modification. *Angew. Chem. Int. Ed.* **57**, 15995–16000 (2018). doi:10.1002/anie.201807942 Medline

22. W. Tang, D. Wang, L. Shao, X. Liu, J. Zheng, Y. Xue, X. Ruan, C. Yang, L. Liu, J. Ma, Z. Li, Y. Liu, LINC00680 and TTN-AS1 stabilized by EIF4A3 promoted malignant

biological behaviors of glioblastoma cells. *Mol. Ther. Nucleic Acids* **19**, 905–921 (2020). doi:10.1016/j.omtn.2019.10.043 Medline

23. X. Wang, Z. Lu, A. Gomez, G. C. Hon, Y. Yue, D. Han, Y. Fu, M. Parisien, Q. Dai, G. Jia, B. Ren, T. Pan, C. He, *N*^{6}-methyladenosine-dependent regulation of messenger RNA stability. *Nature* **505**, 117–120 (2014). doi:10.1038/nature12730 Medline

24. H. Du, Y. Zhao, J. He, Y. Zhang, H. Xi, M. Liu, J. Ma, L. Wu, YTHDF2 destabilizes m^{6}A-containing RNA through direct recruitment of the CCR4-NOT deadenylase complex. *Nat. Commun.* **7**, 12626 (2016). doi:10.1038/ncomms12626 Medline

25. H. Mao, J. J. McMahon, Y.-H. Tsai, Z. Wang, D. L. Silver, Haploinsufficiency for Core Exon Junction Complex Components Disrupts Embryonic Neurogenesis and Causes p53-Mediated Microcephaly. *PLOS Genet.* **12**, e1006282 (2016). doi:10.1371/journal.pgen.1006282 Medline

26. H. Huang, H. Weng, W. Sun, X. Qin, H. Shi, H. Wu, B. S. Zhao, A. Mesquita, C. Liu, C. L. Yuan, Y.-C. Hu, S. Hüttelmaier, J. R. Skibbe, R. Su, X. Deng, L. Dong, M. Sun, C. Li, S. Nachtergaele, Y. Wang, C. Hu, K. Ferchen, K. D. Greis, X. Jiang, M. Wei, L. Qu, J.-L. Guan, C. He, J. Yang, J. Chen, Recognition of RNA *N*^{6}-methyladenosine by IGF2BP proteins enhances mRNA stability and translation. *Nat. Cell Biol.* **20**, 285–295 (2018). doi:10.1038/s41556-018-0045-z Medline

27. X. Wang, B. S. Zhao, I. A. Roundtree, Z. Lu, D. Han, H. Ma, X. Weng, K. Chen, H. Shi, C. He, *N*^{6}-methyladenosine modulates messenger RNA translation efficiency. *Cell* **161**, 1388–1399 (2015). doi:10.1016/j.cell.2015.05.014 Medline

28. J. Liu, K. Li, J. Cai, M. Zhang, X. Zhang, X. Xiong, H. Meng, X. Xu, Z. Huang, J. Peng, J. Fan, C. Yi, Landscape and regulation of m^{6}A and m^{6}A$_m$ methylome across human and mouse tissues. *Mol. Cell* **77**, 426–440.e6 (2020). doi:10.1016/j.molcel.2019.09.032 Medline

29. J. W. Mabin, L. A. Woodward, R. D. Patton, Z. Yi, M. Jia, V. H. Wysocki, R. Bundschuh, G. Singh, The exon junction complex undergoes a compositional switch that alters mRNP structure and nonsense-mediated mRNA decay activity. *Cell Rep.* **25**, 2431–2446.e7 (2018). doi:10.1016/j.celrep.2018.11.046 Medline

30. M. Metkar, H. Ozadam, B. R. Lajoie, M. Imakaev, L. A. Mirny, J. Dekker, M. J. Moore, Higher-order organization principles of pre-translational mRNPs. *Mol. Cell* **72**, 715–726.e3 (2018). doi:10.1016/j.molcel.2018.09.012 Medline

31. H. Le Hir, E. Izaurralde, L. E. Maquat, M. J. Moore, The spliceosome deposits multiple proteins 20-24 nucleotides upstream of mRNA exon-exon junctions. *EMBO J.* **19**, 6860–6869 (2000). doi:10.1093/emboj/19.24.6860 Medline

32. J. Liu, X. Dou, C. Chen, C. Chen, C. Liu, M. M. Xu, S. Zhao, B. Shen, Y. Gao, D. Han, C. He, *N*^{6}-methyladenosine of chromosome-associated regulatory RNA regulates chromatin state and transcription. *Science* **367**, 580–586 (2020). doi:10.1126/science.aay6018 Medline

33. J. Wei, X. Yu, L. Yang, X. Liu, B. Gao, B. Huang, X. Dou, J. Liu, Z. Zou, X.-L. Cui, L.-S. Zhang, X. Zhao, Q. Liu, P. C. He, C. Sepich-Poore, N. Zhong, W. Liu, Y. Li, X. Kou, Y. Zhao, Y. Wu, X. Cheng, C. Chen, Y. An, X. Dong, H. Wang, Q. Shu, Z. Hao, T. Duan, Y.-Y. He, X. Li, S. Gao, Y. Gao, C. He, FTO mediates LINE1 m^{6}A demethylation and

chromatin regulation in mESCs and mouse development. *Science* **376**, 968–973 (2022). doi:10.1126/science.abe9582 Medline

34. P. C. He, X. Dou, Custom scripts associated with "Exon architecture controls mRNA m^{6}A suppression and gene expression", Zenodo (2023); https://doi.org/10.5281/zenodo.7541415.

35. C. C. Uphoff, H. G. Drexler, in *Cancer Cell Culture: Methods and Protocols*, S. P. Langdon, Ed., vol. 88 of *Methods in Molecular Medicine* (Humana Press, 2004), pp. 319–326.

36. Y. Yue, J. Liu, X. Cui, J. Cao, G. Luo, Z. Zhang, T. Cheng, M. Gao, X. Shu, H. Ma, F. Wang, X. Wang, B. Shen, Y. Wang, X. Feng, C. He, J. Liu, VIRMA mediates preferential m^{6}A mRNA methylation in 3'UTR and near stop codon and associates with alternative polyadenylation. *Cell Discov.* **4**, 10 (2018). doi:10.1038/s41421-018-0019-0 Medline

37. Y. Zhou, P. Zeng, Y.-H. Li, Z. Zhang, Q. Cui, SRAMP: Prediction of mammalian N^6-methyladenosine (m^{6}A) sites based on sequence-derived features. *Nucleic Acids Res.* **44**, e91 (2016). doi:10.1093/nar/gkw104 Medline

38. J. Ernst, P. Kheradpour, T. S. Mikkelsen, N. Shoresh, L. D. Ward, C. B. Epstein, X. Zhang, L. Wang, R. Issner, M. Coyne, M. Ku, T. Durham, M. Kellis, B. E. Bernstein, Mapping and analysis of chromatin state dynamics in nine human cell types. *Nature* **473**, 43–49 (2011). doi:10.1038/nature09906 Medline

39. D. Kim, B. Langmead, S. L. Salzberg, HISAT: A fast spliced aligner with low memory requirements. *Nat. Methods* **12**, 357–360 (2015). doi:10.1038/nmeth.3317 Medline

40. Z. Zhang, Q. Zhan, M. Eckert, A. Zhu, A. Chryplewicz, D. F. De Jesus, D. Ren, R. N. Kulkarni, E. Lengyel, C. He, M. Chen, RADAR: Differential analysis of MeRIP-seq data with a random effect model. *Genome Biol.* **20**, 294 (2019). doi:10.1186/s13059-019-1915-9 Medline

41. G. Yu, L.-G. Wang, Y. Han, Q.-Y. He, clusterProfiler: An R package for comparing biological themes among gene clusters. *OMICS* **16**, 284–287 (2012). doi:10.1089/omi.2011.0118 Medline

42. W. J. Kent, C. W. Sugnet, T. S. Furey, K. M. Roskin, T. H. Pringle, A. M. Zahler, D. Haussler, The human genome browser at UCSC. *Genome Res.* **12**, 996–1006 (2002). doi:10.1101/gr.229102 Medline

43. A. M. Bolger, M. Lohse, B. Usadel, Trimmomatic: A flexible trimmer for Illumina sequence data. *Bioinformatics* **30**, 2114–2120 (2014). doi:10.1093/bioinformatics/btu170 Medline

44. S. Anders, P. T. Pyl, W. Huber, HTSeq—A Python framework to work with high-throughput sequencing data. *Bioinformatics* **31**, 166–169 (2015). doi:10.1093/bioinformatics/btu638 Medline

45. B. Langmead, C. Trapnell, M. Pop, S. L. Salzberg, Ultrafast and memory-efficient alignment of short DNA sequences to the human genome. *Genome Biol.* **10**, R25 (2009). doi:10.1186/gb-2009-10-3-r25 Medline

46. M. I. Love, W. Huber, S. Anders, Moderated estimation of fold change and dispersion for RNA-seq data with DESeq2. *Genome Biol.* **15**, 550 (2014). doi:10.1186/s13059-014-0550-8 Medline

47. M. Martin, Cutadapt removes adapter sequences from high-throughput sequencing reads. *EMBnet. J.* **17**, 10 (2011). doi:10.14806/ej.17.1.200

48. A. Dobin, C. A. Davis, F. Schlesinger, J. Drenkow, C. Zaleski, S. Jha, P. Batut, M. Chaisson, T. R. Gingeras, STAR: Ultrafast universal RNA-seq aligner. *Bioinformatics* **29**, 15–21 (2013). doi:10.1093/bioinformatics/bts635 Medline

49. Y. Liao, G. K. Smyth, W. Shi, featureCounts: An efficient general purpose program for assigning sequence reads to genomic features. *Bioinformatics* **30**, 923–930 (2014). doi:10.1093/bioinformatics/btt656 Medline

50. N. T. Ingolia, S. Ghaemmaghami, J. R. S. Newman, J. S. Weissman, Genome-wide analysis in vivo of translation with nucleotide resolution using ribosome profiling. *Science* **324**, 218–223 (2009). doi:10.1126/science.1168978 Medline

51. T. Buschmann, L. V. Bystrykh, Levenshtein error-correcting barcodes for multiplexed DNA sequencing. *BMC Bioinformatics* **14**, 272 (2013). doi:10.1186/1471-2105-14-272 Medline

52. V. Agarwal, G. W. Bell, J.-W. Nam, D. P. Bartel, Predicting effective microRNA target sites in mammalian mRNAs. *eLife* **4**, e05005 (2015). doi:10.7554/eLife.05005 Medline

53. S. Ke, A. Pandya-Jones, Y. Saito, J. J. Fak, C. B. Vågbø, S. Geula, J. H. Hanna, D. L. Black, J. E. Darnell Jr., R. B. Darnell, m^6A mRNA modifications are deposited in nascent pre-mRNA and are not required for splicing but do specify cytoplasmic turnover. *Genes Dev.* **31**, 990–1006 (2017). doi:10.1101/gad.301036.117 Medline

54. F. Yan, A. Al-Kali, Z. Zhang, J. Liu, J. Pang, N. Zhao, C. He, M. R. Litzow, S. Liu, A dynamic N^6-methyladenosine methylome regulates intrinsic and acquired resistance to tyrosine kinase inhibitors. *Cell Res.* **28**, 1062–1076 (2018). doi:10.1038/s41422-018-0097-4 Medline

55. R. Middleton, D. Gao, A. Thomas, B. Singh, A. Au, J. J.-L. Wong, A. Bomane, B. Cosson, E. Eyras, J. E. J. Rasko, W. Ritchie, IRFinder: Assessing the impact of intron retention on mammalian gene expression. *Genome Biol.* **18**, 51 (2017). doi:10.1186/s13059-017-1184-4 Medline

56. C. J. Herrmann, R. Schmidt, A. Kanitz, P. Artimo, A. J. Gruber, M. Zavolan, PolyASite 2.0: A consolidated atlas of polyadenylation sites from 3′ end sequencing. *Nucleic Acids Res.* **48**, D174–D179 (2020). doi:10.1093/nar/gkz918 Medline

57. A. G. Baltz, M. Munschauer, B. Schwanhäusser, A. Vasile, Y. Murakawa, M. Schueler, N. Youngs, D. Penfold-Brown, K. Drew, M. Milek, E. Wyler, R. Bonneau, M. Selbach, C. Dieterich, M. Landthaler, The mRNA-bound proteome and its global occupancy profile on protein-coding transcripts. *Mol. Cell* **46**, 674–690 (2012). doi:10.1016/j.molcel.2012.05.021 Medline

58. M. Bartosovic, H. C. Molares, P. Gregorova, D. Hrossova, G. Kudla, S. Vanacova, N6-methyladenosine demethylase FTO targets pre-mRNAs and regulates alternative splicing and 3′-end processing. *Nucleic Acids Res.* **45**, 11356–11370 (2017). doi:10.1093/nar/gkx778 Medline

59. A. Busch, K. J. Hertel, HEXEvent: A database of Human EXon splicing Events. *Nucleic Acids Res.* **41**, D118–D124 (2013). doi:10.1093/nar/gks969 Medline

60. N. L. Bray, H. Pimentel, P. Melsted, L. Pachter, Near-optimal probabilistic RNA-seq quantification. *Nat. Biotechnol.* **34**, 525–527 (2016). doi:10.1038/nbt.3519 Medline

61. A. Louloupi, E. Ntini, T. Conrad, U. A. V. Ørom, Transient N-6-methyladenosine transcriptome sequencing reveals a regulatory role of m6A in splicing efficiency. *Cell Rep.* **23**, 3429–3437 (2018). doi:10.1016/j.celrep.2018.05.077 Medline

62. K. I. Zhou, H. Shi, R. Lyu, A. C. Wylder, Ż. Matuszek, J. N. Pan, C. He, M. Parisien, T. Pan, Regulation of co-transcriptional pre-mRNA splicing by m^6A through the low-complexity protein hnRNPG. *Mol. Cell* **76**, 70–81.e9 (2019). doi:10.1016/j.molcel.2019.07.005 Medline

63. W. Xiao, S. Adhikari, U. Dahal, Y.-S. Chen, Y.-J. Hao, B.-F. Sun, H.-Y. Sun, A. Li, X.-L. Ping, W.-Y. Lai, X. Wang, H.-L. Ma, C.-M. Huang, Y. Yang, N. Huang, G.-B. Jiang, H.-L. Wang, Q. Zhou, X.-J. Wang, Y.-L. Zhao, Y.-G. Yang, Nuclear m^6A reader YTHDC1 regulates mRNA splicing. *Mol. Cell* **61**, 507–519 (2016). doi:10.1016/j.molcel.2016.01.012 Medline

64. N. Viphakone, I. Sudbery, L. Griffith, C. G. Heath, D. Sims, S. A. Wilson, Co-transcriptional loading of RNA export factors shapes the human transcriptome. *Mol. Cell* **75**, 310–323.e8 (2019). doi:10.1016/j.molcel.2019.04.034 Medline

65. K. M. Neugebauer, Nascent RNA and the Coordination of Splicing with Transcription. *Cold Spring Harb. Perspect. Biol.* **11**, a032227 (2019). doi:10.1101/cshperspect.a032227 Medline

66. J. Akhtar, N. Kreim, F. Marini, G. Mohana, D. Brüne, H. Binder, J.-Y. Roignant, Promoter-proximal pausing mediated by the exon junction complex regulates splicing. *Nat. Commun.* **10**, 521 (2019). doi:10.1038/s41467-019-08381-0 Medline

67. D. L. Silver, D. E. Watkins-Chow, K. C. Schreck, T. J. Pierfelice, D. M. Larson, A. J. Burnetti, H.-J. Liaw, K. Myung, C. A. Walsh, N. Gaiano, W. J. Pavan, The exon junction complex component *Magoh* controls brain size by regulating neural stem cell division. *Nat. Neurosci.* **13**, 551–558 (2010). doi:10.1038/nn.2527 Medline

68. Z. Wang, V. Murigneux, H. Le Hir, Transcriptome-wide modulation of splicing by the exon junction complex. *Genome Biol.* **15**, 551 (2014). doi:10.1186/s13059-014-0551-7 Medline

69. C.-C. Lu, C.-C. Lee, C.-T. Tseng, W.-Y. Tarn, Y14 governs p53 expression and modulates DNA damage sensitivity. *Sci. Rep.* **7**, 45558 (2017). doi:10.1038/srep45558 Medline

70. E. Sendinc, D. Valle-Garcia, A. Jiao, Y. Shi, Analysis of m6A RNA methylation in *Caenorhabditis elegans*. *Cell Discov.* **6**, 47 (2020). doi:10.1038/s41421-020-00186-6 Medline

71. L. Kan, S. Ott, B. Joseph, E. S. Park, W. Dai, R. E. Kleiner, A. Claridge-Chang, E. C. Lai, A neural m^6A/Ythdf pathway is required for learning and memory in *Drosophila*. *Nat. Commun.* **12**, 1458 (2021). doi:10.1038/s41467-021-21537-1 Medline

72. G. Jia, Y. Fu, X. Zhao, Q. Dai, G. Zheng, Y. Yang, C. Yi, T. Lindahl, T. Pan, Y.-G. Yang, C. He, *N*6-methyladenosine in nuclear RNA is a major substrate of the obesity-associated FTO. *Nat. Chem. Biol.* **7**, 885–887 (2011). doi:10.1038/nchembio.687 Medline

73. J. Wei, F. Liu, Z. Lu, Q. Fei, Y. Ai, P. C. He, H. Shi, X. Cui, R. Su, A. Klungland, G. Jia, J. Chen, C. He, Differential m^6A, m^6A_m, and m^1A Demethylation Mediated by FTO in the Cell Nucleus and Cytoplasm. *Mol. Cell* **71**, 973–985.e5 (2018). doi:10.1016/j.molcel.2018.08.011 Medline

74. R. Su, L. Dong, C. Li, S. Nachtergaele, M. Wunderlich, Y. Qing, X. Deng, Y. Wang, X. Weng, C. Hu, M. Yu, J. Skibbe, Q. Dai, D. Zou, T. Wu, K. Yu, H. Weng, H. Huang, K. Ferchen, X. Qin, B. Zhang, J. Qi, A. T. Sasaki, D. R. Plas, J. E. Bradner, M. Wei, G. Marcucci, X. Jiang, J. C. Mulloy, J. Jin, C. He, J. Chen, R-2HG Exhibits Anti-tumor Activity by Targeting FTO/m^6A/MYC/CEBPA Signaling. *Cell* **172**, 90–105.e23 (2018). doi:10.1016/j.cell.2017.11.031 Medline

75. G. Zheng, J. A. Dahl, Y. Niu, P. Fedorcsak, C.-M. Huang, C. J. Li, C. B. Vågbø, Y. Shi, W.-L. Wang, S.-H. Song, Z. Lu, R. P. G. Bosmans, Q. Dai, Y.-J. Hao, X. Yang, W.-M. Zhao, W.-M. Tong, X.-J. Wang, F. Bogdan, K. Furu, Y. Fu, G. Jia, X. Zhao, J. Liu, H. E. Krokan, A. Klungland, Y.-G. Yang, C. He, ALKBH5 is a mammalian RNA demethylase that impacts RNA metabolism and mouse fertility. *Mol. Cell* **49**, 18–29 (2013). doi:10.1016/j.molcel.2012.10.015 Medline

76. S. Zhang, B. S. Zhao, A. Zhou, K. Lin, S. Zheng, Z. Lu, Y. Chen, E. P. Sulman, K. Xie, O. Bögler, S. Majumder, C. He, S. Huang, m^6A Demethylase ALKBH5 Maintains Tumorigenicity of Glioblastoma Stem-like Cells by Sustaining FOXM1 Expression and Cell Proliferation Program. *Cancer Cell* **31**, 591–606.e6 (2017). doi:10.1016/j.ccell.2017.02.013 Medline

77. B. Molinie, J. Wang, K. S. Lim, R. Hillebrand, Z.-X. Lu, N. Van Wittenberghe, B. D. Howard, K. Daneshvar, A. C. Mullen, P. Dedon, Y. Xing, C. C. Giallourakis, m^6A-LAIC-seq reveals the census and complexity of the m^6A epitranscriptome. *Nat. Methods* **13**, 692–698 (2016). doi:10.1038/nmeth.3898 Medline

78. L. Hu, S. Liu, Y. Peng, R. Ge, R. Su, C. Senevirathne, B. T. Harada, Q. Dai, J. Wei, L. Zhang, Z. Hao, L. Luo, H. Wang, Y. Wang, M. Luo, M. Chen, J. Chen, C. He, m^6A RNA modifications are measured at single-base resolution across the mammalian transcriptome. *Nat. Biotechnol.* **40**, 1210–1219 (2022). doi:10.1038/s41587-022-01243-z Medline

79. K. C. H. Ha, B. J. Blencowe, Q. Morris, QAPA: A new method for the systematic analysis of alternative polyadenylation from RNA-seq data. *Genome Biol.* **19**, 45 (2018). doi:10.1186/s13059-018-1414-4 Medline
