# Supplementary material for: Exon architecture controls mRNA m6A suppression and gene expression
Source: Science. Author manuscript; Available in PMC 2024 Feb 17. (PMC9990141; doi:10.1126/science.abj9090)
Supplement: Sup. Table Captions [file NIHMS1874593-supplement-Sup__Table_Captions.pdf]

Table S1 to S6

Table S1. Knockdown efficiency of *EIF4A3, RBM8A, RNPS1, UPF1* in m^6A-meRIP-seq experiments.

Table S2. EIF4A3-suppressed and RNPS1-suppressed splice sites in proximity to EIF4A3-suppressed m^6A regions.

Table S3. Tissue m^6A peaks that contain EJC-suppressed m^6A regions and span exon-intron boundaries.

Table S4. Retained intron tissue m^6A peaks that contain EJC-suppressed m^6A regions.

Table S5. m^6A-QTL-associated m^6A peaks that contain EJC-suppressed m^6A regions.

Table S6. Oligonucleotide sequences, antibodies, and other reagents used in this study.
