# Supplementary material for: Exon architecture controls mRNA m6A suppression and gene expression
Source: Science. Author manuscript; Available in PMC 2024 Feb 17. (PMC9990141; doi:10.1126/science.abj9090)
Supplement: mdar reprod. checklist [file NIHMS1874593-supplement-mdar_reprod__checklist.pdf]

Materials Design Analysis Reporting (MDAR)
Checklist for Authors

The MDAR framework establishes a minimum set of requirements in transparent reporting applicable to studies in the life sciences (see Statement of Task: doi:10.31222/osf.io/9sm4x.). The MDAR checklist is a tool for authors, editors and others seeking to adopt the MDAR framework for transparent reporting in manuscripts and other outputs.   Please refer to the MDAR Elaboration Document for additional context for the MDAR framework.

Materials

Antibodies	Yes (indicate where provided: page no/section/legend)	n/a
For commercial reagents, provide supplier name, catalogue number and RRID, if available.	Table S7	

Cell materials	Yes (indicate where provided: page no/section/legend)	n/a
**Cell lines:** Provide species information, strain. Provide accession number in repository **OR** supplier name, catalog number, clone number, **OR** RRID	Provided in the method section Cell lines.	
**Primary cultures:** Provide species, strain, sex of origin, genetic modification status.		x

Experimental animals	Yes (indicate where provided: page no/section/legend)	n/a
**Laboratory animals:** Provide species, strain, sex, age, genetic modification status. Provide accession number in repository **OR** supplier name, catalog number, clone number, **OR** RRID		x
**Animal observed in or captured from the field:** Provide species, sex and age where possible		x
**Model organisms:** Provide Accession number in repository (where relevant) **OR** RRID		x

Plants and microbes	Yes (indicate where provided: page no/section/legend)	n/a
**Plants:** provide species and strain, unique accession number if available, and source (including location for collected wild specimens)		x
**Microbes:** provide species and strain, unique accession number if available, and source		x

Human research participants	Yes (indicate where provided: page no/section/legend)	n/a
Identify authority granting ethics approval (IRB or equivalent committee(s), provide reference number for approval.		x
Provide statement confirming informed consent obtained from study participants.		x
Report on age and sex for all study participants.		x

Design

Study protocol	Yes (indicate where provided: page no/section/legend)	n/a
For clinical trials, provide the trial registration number **OR** cite DOI in manuscript.		x

Laboratory protocol	Yes (indicate where provided: page no/section/legend)	n/a
Provide DOI or other citation details if detailed step-by-step protocols are available.	Provided and cited in relevant methods sections.	

Experimental study design (statistics details)	Yes (indicate where provided: page no/section/legend)	n/a
State whether and how the following have been done, **or** if they were not carried out.		
Sample size determination		x
Randomisation		x
Blinding		x
Inclusion/exclusion criteria		x

Sample definition and in-laboratory replication	Yes (indicate where provided: page no/section/legend)	n/a
State number of times the experiment was replicated in laboratory	Provided in figure legends, and in method section Statistics and reproducibility	
Define whether data describe technical or biological replicates	Provided in figure legends, and in method section Statistics and reproducibility	

Ethics	Yes (indicate where provided: page no/section/legend)	n/a
Studies involving human participants: State details of authority granting ethics approval (IRB or equivalent committee(s), provide reference number for approval.		x
Studies involving experimental animals: State details of authority granting ethics approval (IRB or equivalent committee(s), provide reference number for approval.		x
Studies involving specimen and field samples: State if relevant permits obtained, provide details of authority approving study; if none were required, explain why.		x

Dual Use Research of Concern (DURC)	Yes (indicate where provided: page no/section/legend)	n/a
If study is subject to dual use research of concern, state the authority granting approval and reference number for the regulatory approval		x

Analysis

Attrition	Yes (indicate where provided: page no/section/legend)	n/a
State if sample or data point from the analysis is excluded, and whether the criteria for exclusion were determined and specified in advance.		x

Statistics	Yes (indicate where provided: page no/section/legend)	n/a
Describe statistical tests used and justify choice of tests.	Provided in figure legends	

Data Availability	Yes (indicate where provided: page no/section/legend)	n/a
State whether newly created datasets are available, including protocols for access or restriction on access.	Yes. All relevant sequencing data within this study have been deposited in the NCBI Gene Expression Omnibus (GEO) and are accessible through GEO series accession number GSE162199. Also, see section **Data and materials availability**.	
If data are publicly available, provide accession number in repository or DOI or URL.	NCBI Gene Expression Omnibus (GEO , GSE162199	
If publicly available data are reused, provide accession number in repository or DOI or URL, where possible.	Datasets used are indicated in the relevant figure legends, and cited in references.	

Code Availability	Yes (indicate where provided: page no/section/legend)	n/a
For all newly generated code and software essential for replicating the main findings of the study:	See **Data and materials availability**, and the methods sections corresponding to each specific analysis.	
State whether the code or software is available.	Yes, see **Data and materials availability**	
If code is publicly available, provide accession number in repository, or DOI or URL.	The software used are described and cited in the method sections corresponding to each specific analysis.	

Reporting

Adherence to community standards	Yes (indicate where provided: page no/section/legend)	n/a
MDAR framework recommends adoption of discipline-specific guidelines, established and endorsed through community initiatives. Journals have their own policy about requiring specific guidelines and recommendations to complement MDAR.		x
State if relevant guidelines (eg., ICMJE, MIBBI, ARRIVE) have been followed, and whether a checklist (eg., CONSORT, PRISMA, ARRIVE) is provided with the manuscript.		x
